# Identification of epigenetic interactions between miRNA and DNA methylation associated with gene expression as potential prognostic markers in bladder cancer

**DOI:** 10.1186/s12920-017-0269-y

**Published:** 2017-05-24

**Authors:** Manu Shivakumar, Younghee Lee, Lisa Bang, Tullika Garg, Kyung-Ah Sohn, Dokyoon Kim

**Affiliations:** 1 Biomedical & Translational Informatics Institute, Geisinger Health System, Danville, PA USA; 2 Department of Biomedical Informatics, University of Utah School of Medicine, Salt Lake City, UT USA; 3 Mowad Urology Department, Geisinger Health System, Danville, PA USA; 4 Department of Software and Computer Engineering, Ajou University, Suwon, South Korea; 5 The Huck Institutes of the Life Sciences, Pennsylvania State University, University Park, PA USA

**Keywords:** Interaction between miRNA and methylation, Integrative analysis, Bladder cancer, TCGA

## Abstract

**Background:**

One of the fundamental challenges in cancer is to detect the regulators of gene expression changes during cancer progression. Through transcriptional silencing of critical cancer-related genes, epigenetic change such as DNA methylation plays a crucial role in cancer. In addition, miRNA, another major component of epigenome, is also a regulator at the post-transcriptional levels that modulate transcriptome changes. However, a mechanistic role of synergistic interactions between DNA methylation and miRNA as epigenetic regulators on transcriptomic changes and its association with clinical outcomes such as survival have remained largely unexplored in cancer.

**Methods:**

In this study, we propose an integrative framework to identify epigenetic interactions between methylation and miRNA associated with transcriptomic changes. To test the utility of the proposed framework, the bladder cancer data set, including DNA methylation, miRNA expression, and gene expression data, from The Cancer Genome Atlas (TCGA) was analyzed for this study.

**Results:**

First, we found 120 genes associated with interactions between the two epigenomic components. Then, 11 significant epigenetic interactions between miRNA and methylation, which target *E2F3*, *CCND1*, *UTP6*, *CDADC1*, *SLC35E3*, *METRNL*, *TPCN2*, *NACC2*, *VGLL4*, and *PTEN*, were found to be associated with survival. To this end, exploration of TCGA bladder cancer data identified epigenetic interactions that are associated with survival as potential prognostic markers in bladder cancer.

**Conclusions:**

Given the importance and prevalence of these interactions of epigenetic events in bladder cancer it is timely to understand further how different epigenetic components interact and influence each other.

**Electronic supplementary material:**

The online version of this article (doi:10.1186/s12920-017-0269-y) contains supplementary material, which is available to authorized users.

## Background

Precision medicine, an emerging approach for disease prevention and treatment strategies based on patients’ environmental and genomic variabilities, is moving toward a new era of future medicine [1]. Since cancer is a disease of the genome, cancer genomics aims to improve personalized medicine through the advanced sequencing technology and analysis of patient tumors to discover new genetic alterations associated with specific cancers. To support advances in developing more effective ways to diagnosis, treat, and prevent cancer, a comprehensive understanding of the underlying genetic architectures that drive different cancers is needed.

One of the fundamental challenges in cancer is to detect the regulators of gene expression changes during cancer progression. Through transcriptional silencing of critical cancer-related genes, epigenetic change such as DNA methylation plays a crucial role in cancer [2]. Cytosine methylation of CpG islands are likely to occur in promoter regions located close to the start of transcription, and hypermethylation in the promoter regions is negatively associated with the mRNA level [3]. For example, the hypermethylation of tumor suppressor genes, which is associated with their inhibition of transcription, is recognized as one of the key features of cancer pathogenesis [4]. On the contrary, CpG methylations in gene body regions are likely to be positively associated with transcript level [3]. In addition to DNA methylation, miRNA, another major component of epigenome, is also a regulator at the post-transcriptional levels that modulate transcriptome changes [5]. miRNAs regulate many cancer-related genes associated with different biological processes such as proliferation, apoptosis, development, and tumorigenesis [6–8]. However, a mechanistic role of synergistic interactions between DNA methylation and miRNA as epigenetic regulators on transcriptomic changes and its association with clinical outcomes such as survival have remained largely unexplored in cancer.

Patients’ variability in multi-omics data, including somatic mutation, copy number alteration (CNA), DNA methylation, miRNA, gene, and protein expression, should be captured simultaneously since cancer is an extremely heterogeneous disease. Large-scale collaborative initiatives such as The Cancer Genome Atlas (TCGA) and The International Cancer Genome Consortium (ICGC) have been generating multi-omics data, mostly using the advanced sequencing technologies, as well as patients’ clinical data. These collaborative initiatives have provided unprecedented opportunities to deepen our understanding of complex mechanisms of cancer for advancing precision medicine [9, 10]. Since different types of genomic data sets are regarded as partially independent from and partially complementary to others, there has been an ever-increasing demand for the development of data integration methodologies [11–13]. Therefore, many data integration methods have been developed to improve prediction of cancer clinical outcomes [14–23].

Previously, we developed a novel graph-based framework that integrates multi-omics data and inter-relationship between omic features to better predict cancer clinical outcomes [21]. Notably, a prediction model showed the great improvement when combining inter-relationship between miRNA and DNA methylation data [21]. The previous study suggested that there might be possible synergistic regulatory mechanisms between miRNA and methylation within the epigenome of cancer-related genes, and further these epigenetic interactions could be associated with clinical outcomes such as survival. Thus, integrating miRNA, DNA methylation and gene expression profiles can aid in extracting new biological knowledge by drawing associations between epigenetic interactions and clinical outcomes in cancer. In this study, we propose an integrative framework to identify epigenetic interactions between methylation and miRNA associated with transcriptomic changes and further detect epigenetic interactions significantly associated with prognosis. To test the utility of the proposed framework, urothelial carcinoma of the bladder data set from TCGA was analyzed for this study. Thus far, no molecularly targeted agents have been approved for treatment of the disease [24].

## Methods

### Data

We obtained the bladder cancer dataset from The Cancer Genome Atlas (TCGA) data portal (https://gdc.cancer.gov/). Data for 403 patients were available with complete RNA-Seq, DNA methylation, miRNA-Seq, and clinical datasets. Demographic characteristics are presented in Table 1. Four data matrices were constructed for each clinical data set with rows indexed by TCGA patient ID and columns using the following metrics: RSEM (RNA-Seq by Expectation Maximization) normalized count (RNA-Seq), beta values (DNA methylation) and reads per million miRNA mapped (miRNA-Seq). Methylation probes with null values and gene expression values containing more than 50% zero values were removed. Then, methylation, RNA-Seq and miRNA-Seq data were log-transformed with base 2. More details about the data can be found here [24].

**Table 1:** Demographic characteristics

Clinical variables	Clinical values (*N* = 403)
Sex (Male/Female)	297/106
Age (Mean/Std)	68.1/10.6
Race (Asian/Black/White/NA)	44/23/320/16
Histological subtype (Non-papillary/Papillary/NA)	270/128/5
Stage (I, II/III, IV/NA)	132/170/1
Smoking status (Smoker/Non-smoker/NA)	109/281/13

After quality control steps, the methylation, miRNA and gene expression profiles contained 382,570 probes, 1,046 miRNAs and 12,657 genes, respectively (Table 2). Clinical data in XML format were downloaded to aggregate the clinical information of the corresponding patient cohort (*N* = 403). The XML file was filtered using the variables ‘days to follow up’, ‘vital status’, ‘days to death’, and ‘days to birth’. To calculate the overall survival, ‘days to death’ was used for expired patients and ‘days to last follow up’ for surviving patients. Since the XML data contained many versions of follow up information, the latest version was used to get ‘days to last follow up’. One sample with a discrepancy in ‘days to death’ was removed.

**Table 2:** TCGA bladder cancer data types used for the analysis

Data type	Platform	Number of features^a^
DNA methylation	Infinium HM450 BeadChip	382,570 probes
miRNA expression	Illumina HiSeq miRNA-Seq	1,046 miRNAs
Gene expression	Illumina HiSeq RNA-Seq	12,657 genes

^a^This is the number of features after QC

### Extracting relationship between methylations, miRNAs and genes

The methylation probes in the TCGA data were mapped to the nearest gene using the open source Illumina methylation platform annotation file. To reduce many false positives of miRNA-target gene interactions, the relationships between miRNAs and their target genes were obtained using miRTarBase, which is a manually collected database of miRNA-target interactions experimentally validated by reporter assay, western blot, microarray and next generation sequencing experiments [25]. Relationships between miRNAs and target genes are many-to-many; thus miRTarBase with 410,621 entries mapped 1,046 miRNAs to 12,657 genes, resulting in 722,812 combinations consisting of 7,922 genes, 146,653 methylation probes and 416 miRNAs.

### Identifying interactions between miRNA and methylation associated with gene expression

Linear regression models were used to determine the significance of the interaction between miRNA and methylation above and beyond the additive effects of each variable alone on gene expression variability. To achieve this goal, we performed a likelihood ratio test (LRT) between the full and reduced model for 722,812 combinations. The full model tested the effects of miRNA + methylation + miRNA*methylation (interaction term) on gene expression. The reduced model tested only the effects of miRNA + methylation. For both models, we adjusted for sex and age. LRT is a well-established statistical test to examine whether the observed difference in model fit is statistically significant. Bonferroni correction was applied to correcting for multiple LRT p-values.

### Overall survival analysis

To identify interactions between miRNA and methylation that are significantly associated with prognosis, Kaplan-Meier overall survival analysis was performed. We graphed the methylation with miRNA expression profiles and classified patients’ profiles into nine subgroups based on three quantiles of miRNA and three quantiles of methylation profiles. We then ran survival analysis for the two extreme subgroups – the first subgroup from the lowest quantiles of miRNA and methylation profiles, and the second subgroup from the top quantiles of both profiles.

### Analysis of differential gene expression

We tested the difference in gene expression levels between subgroups defined based on quantiles of miRNA and methylation. Student’s *T*-test was performed to check significant differential gene expression levels between the subgroup from the lowest quantiles of miRNA and methylation and the subgroup from the top quantiles of the data set. ANOVA was used to test the significance of differential expression levels among four different subgroups, including low methylation & low miRNA (LL), low methylation & high miRNA (LH), high methylation & low miRNA (HL), and high methylation & high miRNA (HH) levels.

## Results

### Identification of interactions between miRNA and methylation associated mRNA levels

Out of 722,812 combinations of miRNA, methylation and genes, 227 interactions between miRNA and methylation were significantly associated with gene expression level (Bonferroni-corrected LRT *p* < 0.05) (Additional file 1: Table S1). The obtained combinations contained 120 genes, 200 methylation probes, 76 miRNAs, respectively. A pathway over-representation analysis using ConsensusPathDB (CPDB) [26] showed that 120 genes were significantly over-represented in 23 Reactome pathways (FDR *q* < 0.05), including many cancer-related pathways such as PI3K/AKT activation, oncogene induced senescence, repression of WNT target genes, etc. (Table 3). To examine histology-specific interactions between miRNA and methylation, patients were divided into two subgroups, papillary and non-papillary groups. Then, we reran the framework to identify subtype-specific epigenetic interactions associated with gene expression levels (Additional file 1: Table S2 and S3). Thirteen genes and 21 genes were observed as papillary-specific and non-papillary-specific genes associated with epigenetic interactions, respectively (Additional file 1: Figure S1, Table S2 and S3).

**Table 3:** Pathway over-representation analysis of 120 genes associated with interactions between miRNA and methylation

Reactome pathway name	*p*-value	FDR *q*-value
Oncogene induced senescence	0.0002	0.019
Deactivation of the beta-catenin transactivating complex	0.0002	0.019
Pre-NOTCH transcription and translation	0.0004	0.023
Oxidative stress induced senescence	0.001	0.025
NCAM1 interactions	0.001	0.025
PIP3 activates AKT signaling	0.002	0.025
PI-3 K cascade:FGFR1	0.002	0.025
PI-3 K cascade:FGFR2	0.002	0.025
PI-3 K cascade:FGFR3	0.002	0.025
PI-3 K cascade:FGFR4	0.002	0.025
PI3K events in ERBB4 signaling	0.002	0.025
PI3K events in ERBB2 signaling	0.002	0.025
Pre-NOTCH expression and processing	0.002	0.025
Cellular senescence	0.002	0.025
PI3K/AKT activation	0.002	0.025
GAB1 signalosome	0.002	0.025
Role of LAT2/NTAL/LAB on calcium mobilization	0.003	0.027
repression of WNT target genes	0.005	0.044
Downstream signaling events of B Cell Receptor (BCR)	0.005	0.047
AKT phosphorylates targets in the cytosol	0.006	0.048
Negative regulation of the PI3K/AKT network	0.006	0.048
Cyclin D associated events in G1	0.007	0.048
G1 phase	0.007	0.048

### Epigenetic interactions associated with survival outcome

**Fig. 1:**
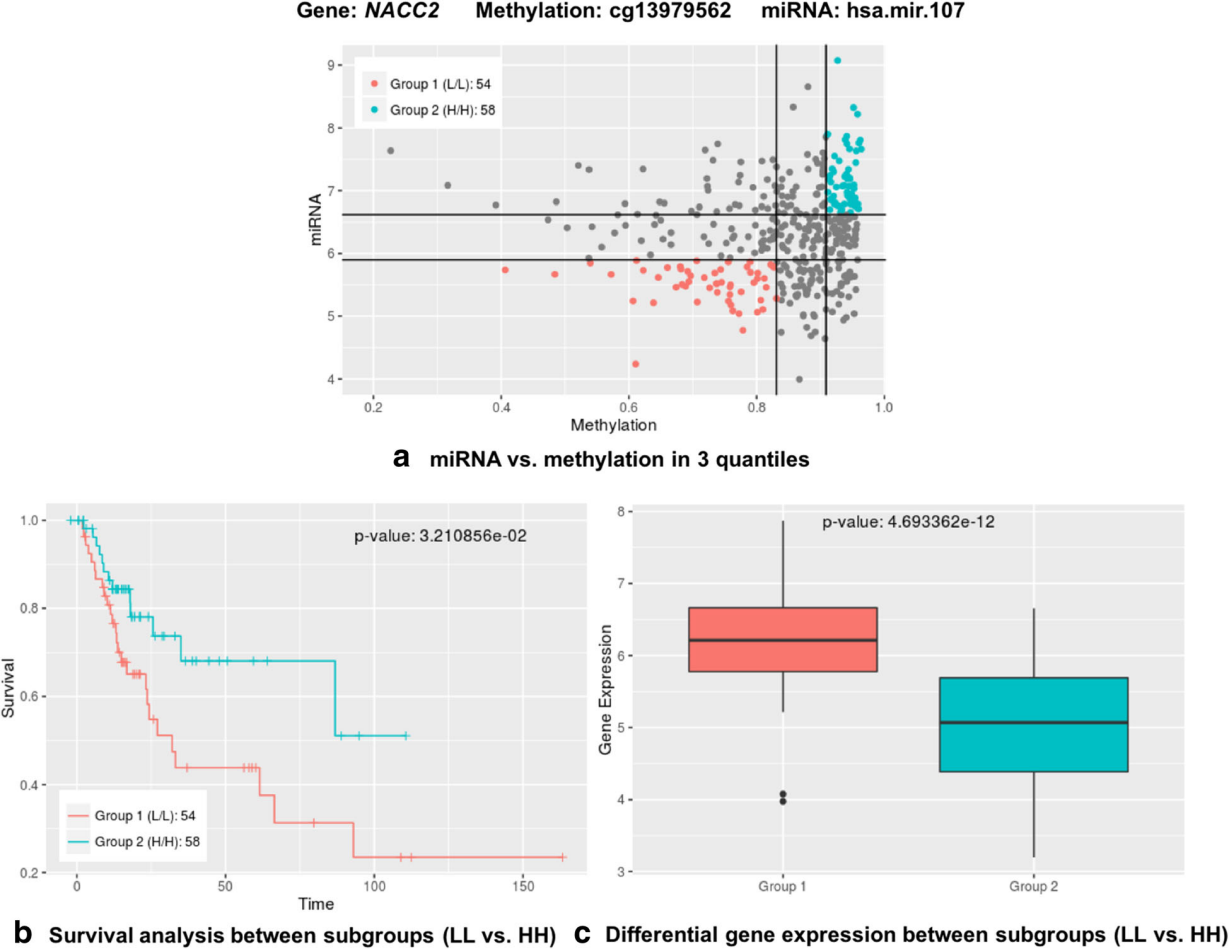
An example result of significant epigenetic interactions between miRNA and methylation on *NACC2* associated with survival outcome. **a** Patients were divided into 9 subgroups based on three quantiles of cg13979562 and hsa-miR-107. **b** Overall survival analysis was performed to show the relative survival in each of the L/L (*N* = 54) and H/H (*N* = 58) subgroups on *NACC2*. **c** Levels of gene expression of *NACC2* between two subgroups (L/L vs. H/H) were significantly different (*p* = 4.69E-12)

To determine whether any of the 227 significant combinations has a significant effect on survival of the patients, an overall survival analysis was performed. Each pair of miRNA and methylation probe from 227 combinations was scatter-plotted, then patients were divided into nine subgroups based on three quantiles of each data set. For example, an interaction between cg13979562 methylation probe and hsa-miR-107 miRNA was significantly associated with gene expression level of *NACC2*. Out of nine subgroups based on three quantiles of levels of methylation probe intensity at cg13979562 and expression of hsa-miR-107, two groups were chosen to be tested whether survival outcome was significantly different: Group 1 (L/L) having low levels of methylation and low levels of miRNA, and Group 2 (H/H) having high levels of methylation and high levels of miRNA (Fig. 1 (a)). Survival analysis showed that L/L (*N* = 54) and H/H (*N* = 58) epigenetic subgroups acting on *NACC2* significantly differ in prognosis (*p* = 3.21e-02), meaning that patients with higher methylation probe intensity at cg13979562 and higher level of hsa-miR-107 expression were associated with a good prognosis (Fig. 1 (b)). Among 227 combinations of gene, miRNA and methylation, 11 combinations acting upon ten genes were found to be significantly associated with survival (*p* < 0.05): *E2F3*, *CCND1*, *UTP6*, *CDADC1*, *SL35E3*, *METRNL*, *TPCN2*, *NACC2*, *VGLL4*, and *PTEN* (Table 4 and Additional file 1: Figure S2). Methylation (on two different probes) of METRNL along with the presence of miR-107 was found to be a significant epigenetic interaction impacting survival outcome. To assess whether these interactions are more significant than expected by chance, methylation and miRNA were randomly selected 1,000 times per gene to generate background data set, then, LRT was performed. These 11 epigenetic interactions were still more significant than expected by chance (Additional file 1: Table S2).

**Table 4:** Summary of overall survival analysis results

Gene	Methylation	miRNA	Bonferroni-corrected LRT *p*-value	*p*-value from survival analysis
E2F3	cg21803390	hsa-miR-217	8.31E-08	2.17E-02
CCND1	cg03040489	hsa-miR-944	3.81E-05	1.08E-02
UTP6	cg13453082	hsa-miR-1254	2.44E-04	4.54E-02
CDADC1	cg17226947	hsa-miR-107	7.37E-04	1.03E-02
SLC35E3	cg02006977	hsa-miR-940	1.08E-03	3.58E-02
METRNL	cg01502876	hsa-miR-107	1.49E-03	1.50E-02
TPCN2	cg10490196	hsa-miR-1976	1.69E-03	3.56E-02
NACC2	cg13979562	hsa-miR-107	1.92E-03	3.21E-02
VGLL4	cg25619837	hsa-miR-3662	9.72E-03	1.89E-02
METRNL	cg03155999	hsa-miR-107	1.15E-02	1.19E-02
PTEN	cg166686761	hsa-miR-543	4.97E-02	4.53E-02

### Differential gene expression between subgroups defined by epigenetic interactions

**Fig. 2:**
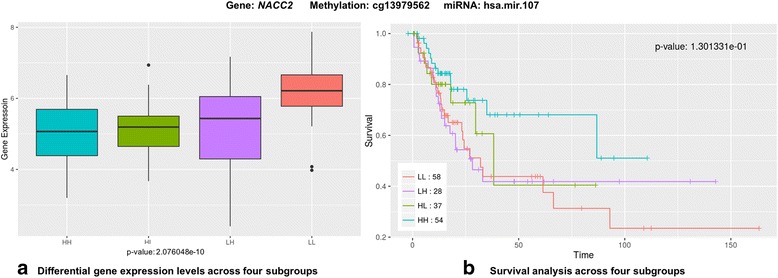
Relative contribution of epigenetic controls on *NACC2*. **a** Significance of differential expression levels among four different subgroups, including low methylation & low miRNA (LL), low methylation & high miRNA (LH), high methylation & low miRNA (HL), and high methylation & high miRNA (HH) levels. **b** Overall survival analysis for 4 subgroups

To test for differential levels of gene expression between subgroups defined by epigenetic interactions, a *T*-test was performed for the 11 combinations that were significantly associated with survival (Additional file 1: Figure S3). For example, Fig. 1c shows the boxplot of gene expression levels between the low epigenetic control cohort (L/L) and the high epigenetic control cohort (H/H) for *NACC2* at cg13979562 and hsa-miR-107 (*p* = 4.49E-12). Epigenetic control led to lower expression levels of *E2F3*, *CCND1*, *CDADC1*, *SLC35E3*, *NACC2*, and *VGLL4*, and the epigenetic control of four genes, *CCND1*, *CDADC1*, *NACC2*, and *VGLL4*, was found to be associated with a worse survival outcome (Additional file 1: Figure S2 and S3). For *UTP6*, *METRNL*, *TPCN2*, and *PTEN*, high epigenetic control was associated with higher or unchanged expression levels of the target genes (Additional file 1: Figure S3). Of these, high epigenetic control led to a worse survival outcome for UTP6 and PTEN (Additional file 1: Figure S2). In addition, we tested the significance of differential expression levels among four different subgroups, including low methylation & low miRNA (LL), low methylation & high miRNA (LH), high methylation & low miRNA (HL), and high methylation & high miRNA (HH) levels. Levels of gene expression of *CDADC1*, *E2F3*, *METRNL*, *NACC2*, *VGLL4* across four subtypes were significantly different (*p* < 0.05) and there were consistent patterns that levels of gene expression decreased gradually based on the levels of epigenetic controls, whereas the gene expression levels of *PTEN* across four subtypes showed the opposite pattern (Additional file 1: Figure S4). Notably, even though *NACC2* mRNA levels were significantly lower in HH, HL, and LH subgroups than in LL subgroups, HH subgroup was only associated with good prognosis, indicating that this signal was achieved by only interactions between miRNA and methylation, not by miRNA or methylation alone (Fig. 2). Other results of overall survival analysis for comparing four subgroups can be found here (Additional file 1: Figure S5).

### Discussion

**Fig. 3:**
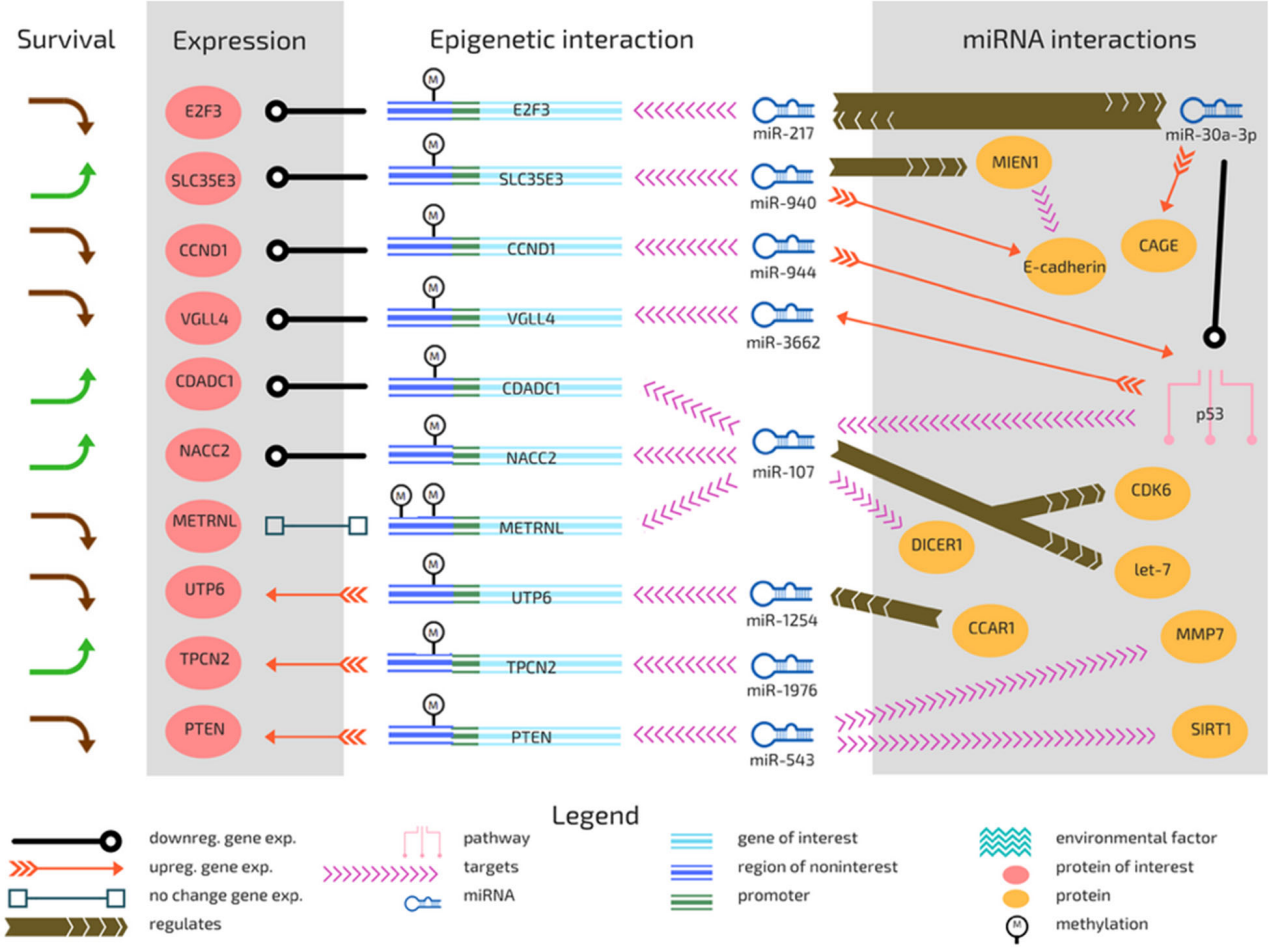
A global view of differential expression patterns emerging from epigenetic control in bladder cancer

DNA methylation downregulates gene expression by inhibiting binding of transcription factors to DNA. Downstream, miRNA silencing, mediated by RNA polymerase II, also works to downregulate gene expression by regulating the processing of mRNA transcripts. We expected miRNA activity targeting methylated genes to decrease expression and worsen survival outcome. We found that a common interaction was one in which there was, as expected, lower gene expression with the presence of epigenetic control, and this phenomenon would tend to lead to a worse survival outcome. Canonical CGI methylation is associated with gene expression silencing, but our results seem to support the previous finding that cancer cells seem to activate CGI methylation of hypomethylated genes which were previously lowly expressed in normal tissues; hypermethylation did not increase the expression of the corresponding genes in cancer cells, but transcription factors were overexpressed [27]. In some cases, the target gene maintained same or higher expression even with microRNA and methylation (*UTP6*, *METRNL*, *TPCN2*, and *PTEN*); for all these except *TPCN2*, this led to a worse survival outcome (Fig. 3). Out of the 120 genes we found ten genes to be associated with the interaction between miRNA silencing and methylation, the following epigenetic interactions were shown to be especially associated with survival outcome in bladder cancer even though expression patterns largely did not show epigenetically-induced simple downregulation.

### Differential miRNA expression patterns in cancer – tumor-suppressing effect

Decreased expression of miR-217 was found to be significantly associated with large tumor size and advanced clinical stage [28], and miR-217 directly suppressed *E2F3* and thereby inhibited invasion of hepatocellular carcinoma [29]. MiR-217 was also found to regulate and be regulated by miR-30a-3p, which suppresses p53 [30]. Previously, introduction of synthetic miR-107 suppressed growth of human non-small cell lung cancer cell lines [31] and high levels of miR-107 were associated with a better survival outcome in gastric cancer [32]. MiR-107 was found to target DICER1 and thereby regulate tumor invasion and metastasis (Fig. 3) [33]. Mutations in DICER1 lead to an abnormally short Dicer protein that is unable to aid in the production of miRNA; Dicer acts as an oncogene or tumor suppressor in varying contexts, including varied roles in bladder cancer (Fig. 3) [34]. MIR-940 levels were found to be the highest in invasive and advanced bladder cancer [35] and has previously been found to inhibit the migratory and invasive potential of cells and increase E-cadherin expression by regulating MIEN1. MiR-940 is highly expressed in immortalized normal cells compared to cancer cells and plays a role in mesenchymal-to-epithelial transition (MET) [36]. MiR-543 is known to target SIRT1 in gastric cancer [37], and miR-543-mediated targeting of SIRT1 is known to alleviate insulin resistance [38]. MMP7 (an oncogene) is also targeted by miR-543 in ovarian cancer; downregulation of miR-543 promotes cancer invasion [39]. The function of miR-1976 is poorly characterized although it was identified as aberrantly expressed in lymphoblastic leukemia [40].

### Differential miRNA expression patterns in cancer – high levels of expression in cancer

MiR-944 is overexpressed in human cervical cancer cells [41]. MiR-944 is located in the intron of the TP63 gene but has its own promoter; however, miR-944 biogenesis is markedly increased by the binding of a TP63 gene product, ΔNp63 protein. Moreover, miR-944 upregulates p53 expression [42]. In making the case for distinct subtypes of bladder cancer, basal and luminal, Choi et al. found that TP63 knockdown (and inferred from that, lessened miR-944) deceased basal pathway gene expression and also increased PPAR pathway gene expression, associated with luminal-type carcinomas. The evidence suggests a complex pathway for the interaction of miR-944 and p63 in encouraging the development of primary basal MIBC and perhaps discouraging the luminal type of MIBC. Plasma-borne levels of miR-944 and miR-3662 has been suggested as possible biomarkers for lung cancer [43]. In hepatocellular carcinoma, miR-3662 was also found to be upregulated by p53 (Fig. 3) [44]. MiR-1254 was suggested as a serum-based miRNA biomarker for early-stage lung cancer [45] and in contrast to canonical miRNAs, its biogenesis is independent of DROSHA [46]; miR-1254 expression enhancement may re-sensitize tamoxifen-resistant breast cancer cells to tamoxifen [47].

### Downregulation of expression of target with high epigenetic control

The pattern of epigenetic control leading to lower expression held for E2F3, CCND1, CDADC1, SLC35E3, NACC2, and VGLL4, and for some of these (CCND1, CDADC1, NACC2, VGLL4), the epigenetic control was found to be associated with a worse survival outcome. CDADC1 is a domain of cytidine and dCMP deaminase, also called NYD-SP15. CDADC1 was found to dynamically shuttle between nucleus and cytoplasm and overexpression of CDADC1 was found to reduce cell growth and block G1 to S phase transition in the cell cycle [48]. It was also dysregulated in liver cancer [49] and involved in regulating testicular development [50]. High epigenetic control of CDADC1 was associated with lower expression and with a worse survival outcome; we posit that lower expression of CDADC1 would encourage cell cycle deregulation (including less breakdown of cytidine, a component of RNA) and thereby worsen survival outcome. NACC2, also known as RBB, is a transcription repressor that is an important regulator of the p53 pathway: NACC2 inhibits the expression of MDM2, which stabilizes p53 expression [51]. VGLL4 is a Hippo pathway member and acts as a YAP agonist [52]; it is said to function as a tumor suppressor in gastric cancer [53], lung cancer [54] and was also included in a smoking cessation quit-success genotype score calculation [55]. As esophageal squamous cell carcinoma progresses, VGLL4 expression is downregulated [56].

CCND1 gene amplification was previously found to be correlated to histopathological tumor characteristics, cancer-specific survival and response to chemotherapy in bladder cancer [57, 58]. CCND1 protein regulates the cell cycle during the G(1)/S transition, is a substrate for SMAD1, and also phosphorylates and inhibits members of the RB protein family, including RB1. When RB1 is phosphorylated, E2F, a transcription factor, disassociates from the RB/E2F complex and allows the E2F target genes to be transcribed. Our model also implicated epigenetic control of a component of E2F, E2F3, as associated with a better survival outcome. MiRNA targeting (miR-577) of E2F3 was found to inhibit gastric cancer cell progression [59], and miRNA targeting of E2F3 and CCND1 (miR-449b) was found to inhibit the proliferation of SW1116 colon cancer stem cells [60].

In a study of metastatic urothelial carcinoma, significant copy number amplifications were found in E2F3 (30% vs. 7% amplification) and CCND1 [61]. For E2F3 and CCND1, we found that the interaction of methylation of the target gene (E2F3/CCND1) and presence of a targeting miRNA was associated with lower expression of the target gene; high epigenetic control was associated with a worse survival outcome for CCND1, but a better survival outcome for E2F3.

For SLC35E3, the lowered expression resulting from the interaction between miRNA targeting and methylation of target, as for E2F3, led to a better survival outcome. SLC35E3, also called Bladder Cancer-Overexpressed gene 1 (BLOV1), acts in transmembrane transport and belongs to the drug/metabolite transporter protein superfamily (Luscombe, MJ, A novel gene which is overexpressed in advanced bladder cancer May 1999, EMBL/GenBank/DDBJ databases). SLC35E3 was also bioinformatically identified as a potential secreted or transmembrane protein [62].

### Maintenance or increase of expression of target with high epigenetic control

For UTP6, METRNL, TPCN2, and PTEN, high epigenetic control was associated with higher expression of the target gene. For UTP6 and PTEN, high epigenetic control led to a worse survival outcome. UTP6 (HCA66) is required for both centriole duplication and ribosome synthesis [63]. Haploinsufficiency-derived UTP6 under-expression in neurofibromatosis type 1 (NF1) cells resulted in those cells being less susceptible to apoptosis [64]. UTP6 was also implicated as element of the interactome of the human histone deacetylase family [65]. PTEN has been shown to be hypermethylated in ovarian cancer cell lines and also highly regulated at the translational level [66], and aberrantly expressed in many forms of cancer [67]. In MIBC squamous epithelia, the expression of PTEN was reduced or lost, and mTOR expression was negatively correlated with PTEN expression only in urothelial squamous cell carcinoma, not schistosomal bladder squamous cell carcinoma [68].

METRNL expression was reduced in the high methylation/high miR-107 (H/H) cohort as compared to the low methylation/low miR-107 (L/L) group for one methylation probe (cg01502876) and unchanged for the other methylation probe (cg03155999), suggesting that methylation of METRNL in cancer will reduce its expression in some cases (Fig. 3). We found that the cohort of patients with high levels of miR-107 and high methylation of its target METRNL at both probes had a better survival outcome. METRNL promotes glucose tolerance and energy expenditure, and blocking METRNL in vivo causes reduces thermogenic gene response and may play a role in tissue inflammation [69]. TPCN2 encodes an NAADP (nicotinic acid adenine dinucleotide phosphate)-induced two-pore Ca(2+) ion channel which is ubiquitously expressed but has elevated expression in liver and kidney and operates as a sensor of both luminal pH and Ca(2+) [70]. TPCN2 is highly sensitive to pH, and is localized intracellularly to endolysosomal organelles [71]. The fact that TPCN2 is induced by nicotinic acid raises questions about potentially novel mechanisms that may underlie well-established link between bladder cancer and smoking.

## Conclusions

In this study, we proposed a novel approach to identify epigenetic interactions between methylation and miRNA associated with prognosis in bladder cancer. We identified 11 significant epigenetic interactions associated with survival (Table 4). In particular, a higher epigenetic control group (HH) on *NACC2* was only associated with good prognosis compared to other subgroups (HL, LH, and LL) (Fig. 2). This suggests that these epigenetic interactions might be new prognostic markers that can be detected by only integrating both methylation and miRNA data, not by miRNA or methylation alone. In addition, a global view of inter-plays of miRNA, methylation, and gene expression could aid in extracting new biological knowledge (Fig. 3). The interactions with miRNAs as targeter and targetee belie potential feedback loops in dictating survival outcome. Intriguingly, double epigenetic control in some cases leads to no change or a slight increase in expression. For UTP6 and PTEN, the presence of epigenetic control does not lead to a change in expression of the target but does lead to a worse survival outcome. For current study, we captured the interactions between miRNA and methylation. However, genomic changes, such as somatic mutations or CNA, could substantially affect the transcriptomic changes and could induce a certain level of bias in estimating the interactions between other epigenetic factors. To better understand the role of epigenetic interactions on gene expression levels, we will improve the current approach to incorporate other omics data as well as a future work.

To this end, exploration of TCGA bladder cancer data identified epigenetic interactions that are associated with survival as a potential prognostic marker. Given the importance and prevalence of these interactions of epigenetics events in bladder cancer it is timely to understand further how different epigenetic components interact and influence each other. Thus, cancer patient’s variability in molecular signatures based on these epigenetic interactions in bladder cancer may lead to better prognostic/treatment strategies for improved precision medicine. Our results warrant further investigation in a larger independent cohort.

## Additional file

Additional file 1: Supplementary information. **Table S1.** Significant epigenetic interactions between miRNA and methylation associated with target genes. **Table S2.** Significant epigenetic interactions between miRNA and methylation associated with target genes for papillary subtype. **Table S3.** Significant epigenetic interactions between miRNA and methylation associated with target genes for non-papillary subtype. **Table S4.** Summary of overall survival analysis results. **Figure S1.** Venn Diagram of Significant target genes for papillary, non-papillary subtypes. **Figure S2.** Survival analysis between two subgroups (LL and HH). **Figure S3.** Gene expression boxplot for two subgroups (LL and HH). **Figure S4.** Gene expression boxplot for four subgroups (LL, LH, HL, and HH). **Figure S5.** Survival analysis across four subgroups (LL, LH, HL, and HH). (DOCX 2368 kb)
