# Supplementary material for: Identification of epigenetic interactions between miRNA and DNA methylation associated with gene expression as potential prognostic markers in bladder cancer
Source: BMC Med Genomics. 2017 May 24;10(Suppl 1):30. doi: 10.1186/s12920-017-0269-y (PMC5461531; doi:10.1186/s12920-017-0269-y)

**Supplementary Information**

**Table S1.** Significant epigenetic interactions between miRNA and methylation associated with target genes.

| **Gene** | **Methylation** | **miRNA** | **LRT p-value** | **Bonferroni corrected p-value** |
| --- | --- | --- | --- | --- |
| BCL3 | cg11459773 | hsa.mir.3936 | 1.65E-15 | 1.19E-09 |
| USP40 | cg05689415 | hsa.mir.484 | 3.86E-15 | 2.79E-09 |
| E2F3 | cg21803390 | hsa.mir.765 | 1.28E-14 | 9.24E-09 |
| NCAN | cg04314308 | hsa.mir.1286 | 1.31E-14 | 9.49E-09 |
| SLC7A1 | cg03937082 | hsa.mir.3919 | 3.09E-14 | 2.23E-08 |
| BCL11B | cg07015803 | hsa.mir.944 | 5.24E-14 | 3.79E-08 |
| C8orf33 | cg01040181 | hsa.mir.3159 | 1.02E-13 | 7.39E-08 |
| E2F3 | cg21803390 | hsa.mir.217 | 1.15E-13 | 8.31E-08 |
| BCL3 | cg01220257 | hsa.mir.3936 | 1.54E-13 | 1.12E-07 |
| TXNL1 | cg18319916 | hsa.mir.320e | 2.77E-13 | 2.0E-07 |
| CDADC1 | cg18587973 | hsa.mir.107 | 9.90E-13 | 7.16E-07 |
| ECH1 | cg07662676 | hsa.mir.320a | 1.11E-12 | 8.02E-07 |
| SLC35E3 | cg02006977 | hsa.mir.3929 | 1.16E-12 | 8.35E-07 |
| NCAN | cg06952310 | hsa.mir.1286 | 1.33E-12 | 9.60E-07 |
| TXNL1 | cg23374762 | hsa.mir.320e | 1.37E-12 | 9.90E-07 |
| PFKP | cg10710457 | hsa.mir.3609 | 2.15E-12 | 1.56E-06 |
| PFKP | cg14068301 | hsa.mir.520b | 3.96E-12 | 2.86E-06 |
| CDKAL1 | cg19926519 | hsa.mir.1276 | 4.92E-12 | 3.55E-06 |
| SLC35E3 | cg02006977 | hsa.mir.3176 | 8.64E-12 | 6.24E-06 |
| SLC12A7 | cg02350636 | hsa.mir.1291 | 8.77E-12 | 6.34E-06 |
| ELAVL4 | cg18242139 | hsa.mir.375 | 1.00E-11 | 7.26E-06 |
| C8orf33 | cg11270233 | hsa.mir.3159 | 1.24E-11 | 8.94E-06 |
| MYO1C | cg05843943 | hsa.mir.3170 | 1.24E-11 | 8.96E-06 |
| ZNF669 | cg06545762 | hsa.mir.1976 | 2.70E-11 | 1.95E-05 |
| C2orf82 | cg13047596 | hsa.mir.3195 | 3.49E-11 | 2.52E-05 |
| MYO1C | cg20929407 | hsa.mir.3170 | 3.97E-11 | 2.87E-05 |
| HIC2 | cg02209075 | hsa.mir.577 | 4.05E-11 | 2.93E-05 |
| UNC5A | cg08322194 | hsa.mir.320a | 4.42E-11 | 3.20E-05 |
| CCND1 | cg03040489 | hsa.mir.944 | 5.28E-11 | 3.81E-05 |
| TXNL1 | cg18319916 | hsa.mir.588 | 5.83E-11 | 4.21E-05 |
| SLC7A1 | cg26117398 | hsa.mir.3919 | 1.04E-10 | 7.54E-05 |
| BCL11B | cg16452866 | hsa.mir.944 | 1.12E-10 | 8.09E-05 |
| MDM2 | cg08547457 | hsa.mir.3662 | 1.71E-10 | 0.000123 |
| MYO1C | cg13588265 | hsa.mir.3198 | 1.74E-10 | 0.000126 |
| BCL3 | cg21606696 | hsa.mir.3936 | 1.77E-10 | 0.000128 |
| SLC12A7 | cg18196463 | hsa.mir.1291 | 1.85E-10 | 0.000134 |
| SLC12A7 | cg22673380 | hsa.mir.1291 | 2.01E-10 | 0.000145 |
| SLC35E3 | cg02006977 | hsa.mir.3652 | 2.12E-10 | 0.000153 |
| METTL6 | cg26710055 | hsa.mir.765 | 2.51E-10 | 0.000181 |
| NIPBL | cg08374525 | hsa.mir.3936 | 2.71E-10 | 0.000196 |
| PFKP | cg02579140 | hsa.mir.520b | 2.88E-10 | 0.000208 |
| GATA6 | cg16204205 | hsa.mir.944 | 2.96E-10 | 0.000214 |
| UTP6 | cg13453082 | hsa.mir.1254 | 3.38E-10 | 0.000244 |
| ANP32E | cg13458596 | hsa.mir.3929 | 3.50E-10 | 0.000253 |
| ZNF154 | cg01268824 | hsa.mir.548y | 3.58E-10 | 0.000259 |
| ZNF555 | cg23671719 | hsa.mir.3929 | 3.63E-10 | 0.000262 |
| SLC35E3 | cg02006977 | hsa.mir.665 | 3.66E-10 | 0.000265 |
| TACR2 | cg23339629 | hsa.mir.1976 | 4.43E-10 | 0.00032 |
| ECH1 | cg02012442 | hsa.mir.320a | 5.25E-10 | 0.000379 |
| TRIM68 | cg23532985 | hsa.mir.1256 | 7.86E-10 | 0.000568 |
| DDHD1 | cg13654573 | hsa.mir.934 | 8.28E-10 | 0.000598 |
| RNASEH2B | cg01060358 | hsa.mir.940 | 8.35E-10 | 0.000603 |
| ERLIN2 | cg09056309 | hsa.mir.326 | 8.78E-10 | 0.000635 |
| HIC2 | cg02209075 | hsa.mir.4310 | 1E-09 | 0.000698 |
| PFKP | cg09875661 | hsa.mir.520b | 1E-09 | 0.000701 |
| CDADC1 | cg17226947 | hsa.mir.107 | 1E-09 | 0.000737 |
| LONRF2 | cg26150922 | hsa.mir.484 | 1.06E-09 | 0.000768 |
| ESPNL | cg02290684 | hsa.mir.375 | 1.15E-09 | 0.000833 |
| ZNF555 | cg23100979 | hsa.mir.3929 | 1.22E-09 | 0.000883 |
| DCTN6 | cg03746590 | hsa.mir.3929 | 1.24E-09 | 0.000894 |
| LRIG1 | cg04560545 | hsa.mir.588 | 1.30E-09 | 0.000942 |
| TRIP10 | cg25510018 | hsa.mir.3609 | 1.34E-09 | 0.000968 |
| SLC25A15 | cg06026880 | hsa.mir.375 | 1.37E-09 | 0.000993 |
| HOXB3 | cg10585948 | hsa.mir.375 | 1.38E-09 | 0.000998 |
| IGF1R | cg15375596 | hsa.mir.1294 | 1.41E-09 | 0.001016 |
| MAPKAPK2 | cg25748647 | hsa.mir.1231 | 1.49E-09 | 0.001074 |
| SLC35E3 | cg02006977 | hsa.mir.940 | 1.50E-09 | 0.001084 |
| TLE3 | cg12349571 | hsa.mir.765 | 1.51E-09 | 0.001091 |
| ERBB2 | cg22018815 | hsa.mir.375 | 1.62E-09 | 0.001169 |
| TES | cg01574564 | hsa.mir.641 | 1.64E-09 | 0.001188 |
| RNASEH2B | cg12175143 | hsa.mir.940 | 1.75E-09 | 0.001263 |
| ZNF555 | cg23100979 | hsa.mir.665 | 1.79E-09 | 0.001294 |
| SHPK | cg21817720 | hsa.mir.943 | 1.84E-09 | 0.001328 |
| HOXB3 | cg13280788 | hsa.mir.375 | 1.91E-09 | 0.001382 |
| MYPN | cg13609017 | hsa.mir.520e | 1.93E-09 | 0.001393 |
| CDKAL1 | cg07905141 | hsa.mir.940 | 1.95E-09 | 0.001407 |
| BCL3 | cg19587318 | hsa.mir.3936 | 2E-09 | 0.001414 |
| BCL3 | cg01220257 | hsa.mir.3115 | 2E-09 | 0.001456 |
| VGLL4 | cg23193320 | hsa.mir.3662 | 2E-09 | 0.001472 |
| METRNL | cg01502876 | hsa.mir.107 | 2.06E-09 | 0.001487 |
| ZNF48 | cg01304182 | hsa.mir.484 | 2.08E-09 | 0.001506 |
| TPCN2 | cg10490196 | hsa.mir.1976 | 2.33E-09 | 0.001686 |
| RIMS4 | cg15207742 | hsa.mir.4254 | 2.64E-09 | 0.001908 |
| NACC2 | cg13979562 | hsa.mir.107 | 2.65E-09 | 0.001916 |
| SLC12A7 | cg01551729 | hsa.mir.527 | 2.71E-09 | 0.001962 |
| RAB11FIP3 | cg07740599 | hsa.mir.484 | 3.08E-09 | 0.002224 |
| KCTD10 | cg09412069 | hsa.mir.375 | 3.20E-09 | 0.00231 |
| ESPNL | cg05099493 | hsa.mir.375 | 3.21E-09 | 0.002322 |
| EHMT1 | cg14156842 | hsa.mir.484 | 3.33E-09 | 0.002409 |
| SLC7A1 | cg02756849 | hsa.mir.3919 | 3.46E-09 | 0.002499 |
| ECHDC1 | cg13817182 | hsa.mir.3174 | 3.51E-09 | 0.002536 |
| PFKP | cg09875661 | hsa.mir.3609 | 3.67E-09 | 0.002653 |
| CDKAL1 | cg07905141 | hsa.mir.1276 | 3.77E-09 | 0.002723 |
| TXNL1 | cg09130402 | hsa.mir.588 | 3.78E-09 | 0.002733 |
| DYRK2 | cg19968199 | hsa.mir.147b | 3.90E-09 | 0.002818 |
| CDK6 | cg07266431 | hsa.mir.449a | 3.93E-09 | 0.002842 |
| NHLRC3 | cg12415720 | hsa.mir.1258 | 4E-09 | 0.002904 |
| STARD3 | cg00871133 | hsa.mir.940 | 4.17E-09 | 0.003011 |
| ANP32E | cg13458596 | hsa.mir.665 | 4.48E-09 | 0.003236 |
| HS6ST1 | cg00221357 | hsa.mir.3658 | 4.50E-09 | 0.003256 |
| ZNF555 | cg23671719 | hsa.mir.665 | 4.90E-09 | 0.003539 |
| SSBP4 | cg07527607 | hsa.mir.3662 | 5.07E-09 | 0.003668 |
| TBX3 | cg13930596 | hsa.mir.137 | 5.23E-09 | 0.003778 |
| DYRK2 | cg19968199 | hsa.mir.3662 | 5.72E-09 | 0.004134 |
| CDADC1 | cg24523585 | hsa.mir.107 | 5.77E-09 | 0.004174 |
| ZBTB38 | cg27288595 | hsa.mir.559 | 6E-09 | 0.004327 |
| MYO1C | cg10431512 | hsa.mir.3198 | 6E-09 | 0.004338 |
| DPY19L1 | cg25557260 | hsa.mir.375 | 6.14E-09 | 0.004437 |
| CACNA1C | cg12046677 | hsa.mir.2110 | 6.28E-09 | 0.004539 |
| PFKP | cg02345060 | hsa.mir.3609 | 6.61E-09 | 0.004781 |
| SLC12A7 | cg07567497 | hsa.mir.1291 | 6.65E-09 | 0.004807 |
| THBS2 | cg05969567 | hsa.mir.3170 | 6.89E-09 | 0.004977 |
| SLC35E3 | cg15993521 | hsa.mir.665 | 7.10E-09 | 0.005129 |
| TNRC6A | cg27078729 | hsa.mir.23c | 7.13E-09 | 0.005154 |
| LRPAP1 | cg25296745 | hsa.mir.3609 | 7.23E-09 | 0.005229 |
| NACC2 | cg02493986 | hsa.mir.107 | 7.27E-09 | 0.005253 |
| ERBB2 | cg05512684 | hsa.mir.375 | 7.42E-09 | 0.005361 |
| RNF185 | cg24245352 | hsa.mir.3185 | 7.48E-09 | 0.005406 |
| CCDC6 | cg08096168 | hsa.mir.3646 | 7.68E-09 | 0.005554 |
| E2F3 | cg21803390 | hsa.mir.607 | 8.08E-09 | 0.00584 |
| SLC35E3 | cg16615842 | hsa.mir.665 | 8.25E-09 | 0.005965 |
| BCL3 | cg21606696 | hsa.mir.3115 | 8.75E-09 | 0.006325 |
| ZNF154 | cg26465391 | hsa.mir.548y | 9.44E-09 | 0.006821 |
| PFKP | cg14068301 | hsa.mir.520e | 1.04E-08 | 0.0075 |
| POLR3D | cg08316073 | hsa.mir.3183 | 1.07E-08 | 0.007756 |
| INTU | cg22357337 | hsa.mir.3941 | 1.08E-08 | 0.007809 |
| FAM83G | cg11281396 | hsa.mir.548s | 1.12E-08 | 0.008063 |
| PITX1 | cg02037307 | hsa.mir.320a | 1.14E-08 | 0.00823 |
| STX1B | cg25033993 | hsa.mir.4254 | 1.25E-08 | 0.009058 |
| HOXB3 | cg24761525 | hsa.mir.375 | 1.26E-08 | 0.009139 |
| DOCK7 | cg01097102 | hsa.mir.612 | 1.26E-08 | 0.009141 |
| CLEC16A | cg04434701 | hsa.mir.484 | 1.28E-08 | 0.009266 |
| ZNF154 | cg12506930 | hsa.mir.548y | 1.29E-08 | 0.009358 |
| CACNB2 | cg07372450 | hsa.mir.2110 | 1.31E-08 | 0.009463 |
| CDKAL1 | cg07914385 | hsa.mir.4284 | 1.34E-08 | 0.009712 |
| VGLL4 | cg25619837 | hsa.mir.3662 | 1.35E-08 | 0.009724 |
| SEC16A | cg01275932 | hsa.mir.484 | 1.37E-08 | 0.009886 |
| ERBB2 | cg26615017 | hsa.mir.375 | 1.39E-08 | 0.010039 |
| PFKP | cg20133200 | hsa.mir.520e | 1.46E-08 | 0.010524 |
| AKT2 | cg26862316 | hsa.mir.137 | 1.5E-08 | 0.010858 |
| ZNF555 | cg08069425 | hsa.mir.3929 | 1.53E-08 | 0.011078 |
| SLC35E3 | cg16615842 | hsa.mir.3652 | 1.56E-08 | 0.011289 |
| TXNL1 | cg06765434 | hsa.mir.320e | 1.57E-08 | 0.011383 |
| DIP2C | cg07236840 | hsa.mir.375 | 1.59E-08 | 0.01146 |
| METRNL | cg03155999 | hsa.mir.107 | 1.59E-08 | 0.011514 |
| RNF185 | cg12504284 | hsa.mir.3185 | 1.6E-08 | 0.011564 |
| WRN | cg18996590 | hsa.mir.592 | 1.62E-08 | 0.011695 |
| PFKP | cg20133200 | hsa.mir.520b | 1.65E-08 | 0.011939 |
| HOXB3 | cg21474786 | hsa.mir.375 | 1.69E-08 | 0.01218 |
| E2F3 | cg23167585 | hsa.mir.1260b | 1.69E-08 | 0.012251 |
| PFKP | cg15319824 | hsa.mir.520b | 1.76E-08 | 0.012711 |
| RIMS3 | cg24517738 | hsa.mir.375 | 1.77E-08 | 0.012797 |
| SLC12A7 | cg17733824 | hsa.mir.527 | 1.79E-08 | 0.01293 |
| SLC12A7 | cg25528709 | hsa.mir.1291 | 1.8E-08 | 0.013008 |
| CAMKK2 | cg04615865 | hsa.mir.3198 | 1.83E-08 | 0.013214 |
| MYO1C | cg12989085 | hsa.mir.3198 | 1.85E-08 | 0.013403 |
| ZNF555 | cg21355100 | hsa.mir.665 | 1.89E-08 | 0.013687 |
| CDKAL1 | cg18868372 | hsa.mir.940 | 1.9E-08 | 0.013699 |
| HOXB3 | cg04800503 | hsa.mir.375 | 1.97E-08 | 0.014275 |
| POLR3D | cg06021753 | hsa.mir.3651 | 1.99E-08 | 0.014413 |
| DYRK2 | cg19968199 | hsa.mir.3941 | 2.07E-08 | 0.014934 |
| RGS19 | cg00759427 | hsa.mir.3175 | 2.09E-08 | 0.015084 |
| ELAVL4 | cg26301690 | hsa.mir.375 | 2.12E-08 | 0.015294 |
| AZIN1 | cg16998490 | hsa.mir.643 | 2.16E-08 | 0.015649 |
| BCL3 | cg25644380 | hsa.mir.3936 | 2.23E-08 | 0.016098 |
| PFKP | cg02579140 | hsa.mir.520e | 2.23E-08 | 0.01612 |
| ZNRF2 | cg07003744 | hsa.mir.507 | 2.25E-08 | 0.016275 |
| PLXNA3 | cg09673331 | hsa.mir.612 | 2.32E-08 | 0.016739 |
| GATA6 | cg20921890 | hsa.mir.944 | 2.33E-08 | 0.016835 |
| QRSL1 | cg21190634 | hsa.mir.4284 | 2.33E-08 | 0.016851 |
| SLC7A1 | cg00953201 | hsa.mir.3919 | 2.34E-08 | 0.016911 |
| BCL2 | cg14544831 | hsa.mir.375 | 2.45E-08 | 0.01774 |
| ZNF48 | cg00711896 | hsa.mir.484 | 2.49E-08 | 0.01801 |
| BCL3 | cg17883035 | hsa.mir.3936 | 2.58E-08 | 0.018667 |
| KLF2 | cg18473733 | hsa.mir.940 | 2.63E-08 | 0.019042 |
| E2F3 | cg15585670 | hsa.mir.577 | 2.65E-08 | 0.019188 |
| SEMA7A | cg27157619 | hsa.mir.577 | 2.69E-08 | 0.01942 |
| DIP2C | cg27081107 | hsa.mir.375 | 2.72E-08 | 0.019696 |
| RIMS4 | cg12343182 | hsa.mir.4254 | 2.77E-08 | 0.020011 |
| QSOX2 | cg14249808 | hsa.mir.602 | 2.8E-08 | 0.020256 |
| SLC12A7 | cg24059022 | hsa.mir.1291 | 2.82E-08 | 0.020374 |
| SLC12A7 | cg18756954 | hsa.mir.1291 | 2.87E-08 | 0.020722 |
| IGF1R | cg20388729 | hsa.mir.940 | 3.03E-08 | 0.021904 |
| MAP2K4 | cg18156845 | hsa.mir.559 | 3.11E-08 | 0.022463 |
| NCAN | cg25814383 | hsa.mir.1286 | 3.11E-08 | 0.022491 |
| NFIX | cg08616111 | hsa.mir.3648 | 3.12E-08 | 0.022521 |
| STEAP2 | cg27367526 | hsa.mir.940 | 3.13E-08 | 0.022641 |
| NEK9 | cg08310116 | hsa.mir.940 | 3.14E-08 | 0.022675 |
| CDKAL1 | cg23560765 | hsa.mir.1276 | 3.26E-08 | 0.023583 |
| E2F3 | cg27250759 | hsa.mir.1260b | 3.31E-08 | 0.023957 |
| DLEU1 | cg17004212 | hsa.mir.940 | 3.51E-08 | 0.025399 |
| DIP2C | cg10796899 | hsa.mir.375 | 3.51E-08 | 0.025404 |
| BCL3 | cg11459773 | hsa.mir.3115 | 3.53E-08 | 0.025511 |
| HOXA3 | cg24360871 | hsa.mir.577 | 3.75E-08 | 0.027081 |
| ITPKB | cg05306109 | hsa.mir.133b | 3.76E-08 | 0.027168 |
| STRADB | cg04842541 | hsa.mir.1197 | 3.79E-08 | 0.027372 |
| FHIT | cg00506250 | hsa.mir.217 | 3.86E-08 | 0.027917 |
| HOXB3 | cg21399057 | hsa.mir.375 | 3.88E-08 | 0.028011 |
| RIN2 | cg26396492 | hsa.mir.429 | 3.92E-08 | 0.02833 |
| STRADB | cg24749970 | hsa.mir.1197 | 4E-08 | 0.028941 |
| FBXO46 | cg18429235 | hsa.mir.3115 | 4.05E-08 | 0.029295 |
| RASSF2 | cg16385758 | hsa.mir.429 | 4.56E-08 | 0.032995 |
| BASP1 | cg08771731 | hsa.mir.1255a | 4.58E-08 | 0.033114 |
| PTEN | cg16686761 | hsa.mir.492 | 4.69E-08 | 0.03393 |
| APP | cg23269692 | hsa.mir.1276 | 4.92E-08 | 0.035597 |
| TMED8 | cg14875498 | hsa.mir.375 | 4.93E-08 | 0.035654 |
| PARK2 | cg27630180 | hsa.mir.548s | 4.93E-08 | 0.035667 |
| ATP1A2 | cg05808709 | hsa.mir.484 | 5E-08 | 0.036137 |
| HOXB3 | cg12570134 | hsa.mir.520b | 5.03E-08 | 0.036344 |
| MLH1 | cg12790037 | hsa.mir.658 | 5.07E-08 | 0.036615 |
| STEAP2 | cg02206373 | hsa.mir.940 | 5.14E-08 | 0.03718 |
| BCL2L13 | cg23077364 | hsa.mir.449a | 5.29E-08 | 0.038247 |
| TCF7L2 | cg00831931 | hsa.mir.1305 | 5.42E-08 | 0.039205 |
| MYO1C | cg20929407 | hsa.mir.3198 | 5.53E-08 | 0.039971 |
| HNRNPD | cg01942445 | hsa.mir.2110 | 5.55E-08 | 0.040097 |
| SLC12A7 | cg16017429 | hsa.mir.1291 | 5.63E-08 | 0.040674 |
| ECHDC1 | cg00886812 | hsa.mir.3174 | 5.71E-08 | 0.041244 |
| PTEN | cg16686761 | hsa.mir.543 | 5.75E-08 | 0.041556 |
| MYO1C | cg13588265 | hsa.mir.3170 | 5.89E-08 | 0.042564 |
| HOXB3 | cg13293524 | hsa.mir.375 | 6.28E-08 | 0.045404 |
| SLC35E3 | cg02006977 | hsa.mir.466 | 6.31E-08 | 0.045611 |
| ITPKB | cg03199014 | hsa.mir.133b | 6.37E-08 | 0.046038 |
| C8orf33 | cg06443863 | hsa.mir.3159 | 6.49E-08 | 0.046919 |
| E2F3 | cg21803390 | hsa.mir.1260b | 6.57E-08 | 0.047454 |
| AAK1 | cg04694389 | hsa.mir.612 | 6.68E-08 | 0.048264 |
| XIAP | cg02877744 | hsa.mir.466 | 6.69E-08 | 0.048335 |
| TBX3 | cg25058261 | hsa.mir.137 | 6.88E-08 | 0.04975 |

**Table S2.** Significant epigenetic interactions between miRNA and methylation associated with target genes for papillary subtype.

| **Gene** | **Methylation** | **miRNA** | **LRT p-value** | **Bonferroni corrected p-value** |
| --- | --- | --- | --- | --- |
| ZNF669 | cg07945013 | hsa.mir.548s | 6.86E-13 | 4.96E-07 |
| PFKP | cg19416623 | hsa.mir.3609 | 1.11E-12 | 8.01E-07 |
| ZNF669 | cg07945013 | hsa.mir.600 | 1.27E-12 | 9.19E-07 |
| COL19A1* | cg15080430 | hsa.mir.581 | 1.91E-12 | 1.38E-06 |
| SLC7A1 | cg02756849 | hsa.mir.375 | 1.24E-11 | 9.00E-06 |
| ZNF669 | cg07945013 | hsa.mir.940 | 1.39E-11 | 1.00E-05 |
| BAHD1* | cg21127508 | hsa.mir.3125 | 1.56E-11 | 1.13E-05 |
| SLC7A1 | cg02756849 | hsa.mir.3916 | 5.81E-11 | 4.20E-05 |
| HS6ST1 | cg15920655 | hsa.mir.3658 | 7.60E-11 | 5.49E-05 |
| SLC12A7 | cg18848012 | hsa.mir.1291 | 8.02E-11 | 5.80E-05 |
| CCDC6 | cg15232971 | hsa.mir.3646 | 1.54E-10 | 0.000111 |
| ZNF669 | cg07945013 | hsa.mir.588 | 1.65E-10 | 0.000119 |
| CCDC6 | cg10515953 | hsa.mir.520e | 1.95E-10 | 0.000141 |
| HS6ST1 | cg05729804 | hsa.mir.3658 | 2.59E-10 | 0.000187 |
| HS6ST1 | cg02886528 | hsa.mir.3658 | 2.60E-10 | 0.000188 |
| TMCO1* | cg26272983 | hsa.mir.3945 | 3.46E-10 | 0.00025 |
| HS6ST1 | cg09375299 | hsa.mir.3658 | 5.97E-10 | 0.000431 |
| TMCO1* | cg24106943 | hsa.mir.940 | 6.47E-10 | 0.000468 |
| CCDC6 | cg10515953 | hsa.mir.3646 | 7.94E-10 | 0.000574 |
| SLC7A1 | cg02756849 | hsa.mir.3919 | 1.24E-09 | 0.000896 |
| CCDC6 | cg14316118 | hsa.mir.3646 | 1.28E-09 | 0.000923 |
| SLC1A5* | cg00782174 | hsa.mir.520e | 1.29E-09 | 0.000931 |
| HS6ST1 | cg08867399 | hsa.mir.3658 | 1.33E-09 | 0.000961 |
| TMCO1* | cg24409356 | hsa.mir.3945 | 1.33E-09 | 0.000963 |
| SLC1A5* | cg00782174 | hsa.mir.520b | 1.39E-09 | 0.001003 |
| PFKP | cg02579140 | hsa.mir.520b | 1.56E-09 | 0.001126 |
| TMCO1* | cg18174222 | hsa.mir.3652 | 1.74E-09 | 0.001261 |
| CCDC6 | cg07312556 | hsa.mir.520e | 2.62E-09 | 0.001897 |
| HS6ST1 | cg23429047 | hsa.mir.3658 | 2.79E-09 | 0.002017 |
| RPH3AL* | cg01760178 | hsa.mir.3170 | 2.92E-09 | 0.002111 |
| HNF1A* | cg14101638 | hsa.mir.320a | 3.08E-09 | 0.002226 |
| TMCO1* | cg24409356 | hsa.mir.940 | 3.25E-09 | 0.002351 |
| KRAS* | cg20199655 | hsa.mir.3125 | 3.78E-09 | 0.002733 |
| SLC12A7 | cg13299707 | hsa.mir.1291 | 3.84E-09 | 0.002776 |
| PRKACB* | cg23052793 | hsa.mir.520b | 4.02E-09 | 0.002908 |
| BAHD1* | cg02726739 | hsa.mir.3125 | 4.85E-09 | 0.003503 |
| RNASEH2B | cg08146111 | hsa.mir.3929 | 5.27E-09 | 0.003806 |
| TRIM13* | cg04603834 | hsa.mir.137 | 7.34E-09 | 0.005304 |
| PFKP | cg14068301 | hsa.mir.520b | 7.95E-09 | 0.005749 |
| ZNF415* | cg10332700 | hsa.mir.3941 | 8.42E-09 | 0.006089 |
| E2F3 | cg05617744 | hsa.mir.577 | 9.27E-09 | 0.006697 |
| TMCO1* | cg24106943 | hsa.mir.3945 | 9.49E-09 | 0.006862 |
| CCDC6 | cg10515953 | hsa.mir.520b | 9.78E-09 | 0.007072 |
| LIMCH1* | cg11076306 | hsa.mir.137 | 1.30E-08 | 0.00937 |
| HS6ST1 | cg07892413 | hsa.mir.3658 | 1.47E-08 | 0.010631 |
| SLC1A5* | cg00782174 | hsa.mir.498 | 1.67E-08 | 0.012076 |
| TMCO1* | cg19769164 | hsa.mir.3945 | 1.79E-08 | 0.012964 |
| HNRNPD | cg01942445 | hsa.mir.2110 | 1.84E-08 | 0.013292 |
| BAHD1* | cg18073240 | hsa.mir.3125 | 1.94E-08 | 0.014006 |
| CCDC6 | cg08096168 | hsa.mir.520b | 2.07E-08 | 0.014948 |
| PFKP | cg15319824 | hsa.mir.520b | 2.20E-08 | 0.015903 |
| FANCF | cg08082142 | hsa.mir.548s | 2.88E-08 | 0.020849 |
| CCDC6 | cg08096168 | hsa.mir.520e | 3.13E-08 | 0.022655 |
| HOXA3 | cg02000808 | hsa.mir.3131 | 3.20E-08 | 0.023155 |
| PFKP | cg09875661 | hsa.mir.3609 | 3.77E-08 | 0.027253 |
| TMCO1* | cg19769164 | hsa.mir.940 | 4.33E-08 | 0.031274 |
| CCDC6 | cg07312556 | hsa.mir.520b | 4.34E-08 | 0.031382 |
| KCNK12* | cg04431946 | hsa.mir.3650 | 4.71E-08 | 0.034021 |
| HNF1A* | cg01341572 | hsa.mir.320a | 5.31E-08 | 0.038405 |
| ARMCX6* | cg08464305 | hsa.mir.1262 | 5.45E-08 | 0.039364 |
| CCDC6 | cg15232971 | hsa.mir.520e | 5.58E-08 | 0.0403 |
| HOXA3 | cg24272697 | hsa.mir.3131 | 6.34E-08 | 0.045803 |
| RNASEH2B | cg22221131 | hsa.mir.3929 | 6.83E-08 | 0.04934 |

* Papillary subtype-specific genes compared to the non-papillary and all patient groups

**Table S3.** Significant epigenetic interactions between miRNA and methylation associated with target genes for non-papillary subtype.

| **Gene** | **Methylation** | **miRNA** | **LRT p-value** | **Bonferroni corrected p-value** |
| --- | --- | --- | --- | --- |
| BCL3 | cg11459773 | hsa.mir.3936 | 2.97E-15 | 2.15E-09 |
| USP40 | cg05689415 | hsa.mir.484 | 3.50E-14 | 2.53E-08 |
| C8orf33 | cg11270233 | hsa.mir.3159 | 3.77E-14 | 2.73E-08 |
| FAM83H* | cg08895928 | hsa.mir.3937 | 2.65E-13 | 1.92E-07 |
| BCL3 | cg01220257 | hsa.mir.3936 | 4.32E-13 | 3.12E-07 |
| NCAN | cg04314308 | hsa.mir.1286 | 7.52E-13 | 5.44E-07 |
| C8orf33 | cg01040181 | hsa.mir.3159 | 1.48E-12 | 1.07E-06 |
| TXNL1 | cg18319916 | hsa.mir.320e | 1.61E-12 | 1.17E-06 |
| BCL3 | cg26157948 | hsa.mir.3115 | 3.04E-12 | 2.20E-06 |
| TXNL1 | cg23374762 | hsa.mir.320e | 4.25E-12 | 3.07E-06 |
| E2F3 | cg21803390 | hsa.mir.765 | 8.77E-12 | 6.34E-06 |
| BCL3 | cg19587318 | hsa.mir.3115 | 2.69E-11 | 1.94E-05 |
| SLC35E3 | cg16615842 | hsa.mir.665 | 3.23E-11 | 2.33E-05 |
| ELAVL4 | cg18242139 | hsa.mir.375 | 3.38E-11 | 2.44E-05 |
| TPCN2 | cg10490196 | hsa.mir.449a | 5.08E-11 | 3.67E-05 |
| NCAN | cg06952310 | hsa.mir.1286 | 5.47E-11 | 3.95E-05 |
| BCL11B | cg07015803 | hsa.mir.944 | 6.58E-11 | 4.76E-05 |
| CDADC1 | cg17226947 | hsa.mir.107 | 8.92E-11 | 6.45E-05 |
| GATA6 | cg16204205 | hsa.mir.944 | 9.17E-11 | 6.63E-05 |
| BCL3 | cg19587318 | hsa.mir.3936 | 9.24E-11 | 6.68E-05 |
| SEC16A | cg01275932 | hsa.mir.484 | 1.34E-10 | 9.65E-05 |
| METTL6 | cg26710055 | hsa.mir.765 | 1.51E-10 | 0.000109 |
| RNASEH2B | cg26847093 | hsa.mir.3929 | 1.70E-10 | 0.000123 |
| C2orf82 | cg13047596 | hsa.mir.3195 | 1.72E-10 | 0.000124 |
| BCL3 | cg11459773 | hsa.mir.3115 | 1.89E-10 | 0.000137 |
| BCL3 | cg21606696 | hsa.mir.3936 | 2.09E-10 | 0.000151 |
| SHPK | cg21817720 | hsa.mir.943 | 2.18E-10 | 0.000158 |
| SHPK | cg12950012 | hsa.mir.943 | 2.87E-10 | 0.000207 |
| CDKAL1 | cg07914385 | hsa.mir.1276 | 3.04E-10 | 0.000219 |
| TRIM68 | cg23532985 | hsa.mir.1256 | 3.21E-10 | 0.000232 |
| TXNL1 | cg18319916 | hsa.mir.588 | 3.23E-10 | 0.000233 |
| KLHL26* | cg16705777 | hsa.mir.940 | 3.60E-10 | 0.00026 |
| DPY19L1 | cg25557260 | hsa.mir.375 | 3.91E-10 | 0.000283 |
| BCL3 | cg01220257 | hsa.mir.3115 | 4.16E-10 | 0.000301 |
| SHPK | cg17745562 | hsa.mir.943 | 4.48E-10 | 0.000323 |
| E2F3 | cg21803390 | hsa.mir.217 | 5.04E-10 | 0.000365 |
| LONRF2 | cg26150922 | hsa.mir.484 | 8.62E-10 | 0.000623 |
| BCL3 | cg21606696 | hsa.mir.3115 | 8.80E-10 | 0.000636 |
| MAP2K4 | cg18156845 | hsa.mir.559 | 1.00E-09 | 0.000723 |
| NEK9 | cg20071051 | hsa.mir.940 | 1.12E-09 | 0.00081 |
| MYO1C | cg20929407 | hsa.mir.3170 | 1.26E-09 | 0.000907 |
| RGS19 | cg00759427 | hsa.mir.3175 | 1.47E-09 | 0.001061 |
| HOXA3 | cg24360871 | hsa.mir.577 | 1.61E-09 | 0.001164 |
| CDKAL1 | cg19926519 | hsa.mir.1276 | 1.79E-09 | 0.001296 |
| SLC35E3 | cg16615842 | hsa.mir.3652 | 2.04E-09 | 0.001477 |
| TPCN2 | cg18053228 | hsa.mir.449a | 2.38E-09 | 0.001718 |
| ELAVL4 | cg26301690 | hsa.mir.578 | 2.54E-09 | 0.001835 |
| ERBB2 | cg22018815 | hsa.mir.375 | 2.95E-09 | 0.00213 |
| SLC12A7 | cg01551729 | hsa.mir.527 | 3.22E-09 | 0.002326 |
| F2* | cg26453360 | hsa.mir.3655 | 3.61E-09 | 0.002609 |
| R3HDM1* | cg08333804 | hsa.mir.320a | 3.69E-09 | 0.002665 |
| MYO1C | cg05843943 | hsa.mir.3170 | 3.80E-09 | 0.002745 |
| SLC12A7 | cg23091824 | hsa.mir.1291 | 4.81E-09 | 0.003474 |
| CDKAL1 | cg07914385 | hsa.mir.4284 | 4.87E-09 | 0.003522 |
| NDUFS1* | cg24330485 | hsa.mir.1976 | 5.02E-09 | 0.003631 |
| EHHADH* | cg19087039 | hsa.mir.1291 | 5.03E-09 | 0.003633 |
| BCL11B | cg16452866 | hsa.mir.944 | 5.38E-09 | 0.003892 |
| SLC12A7 | cg17733824 | hsa.mir.527 | 5.68E-09 | 0.004103 |
| MAST3* | cg02720566 | hsa.mir.940 | 5.73E-09 | 0.004144 |
| RIMS4 | cg15207742 | hsa.mir.4254 | 6.32E-09 | 0.004567 |
| BCL3 | cg26157948 | hsa.mir.3936 | 6.98E-09 | 0.005045 |
| HIC2 | cg02209075 | hsa.mir.577 | 7.44E-09 | 0.00538 |
| FANCF | cg00146096 | hsa.mir.548s | 7.83E-09 | 0.005658 |
| NEK9 | cg07098966 | hsa.mir.940 | 8.03E-09 | 0.005802 |
| SEC16A | cg13407274 | hsa.mir.484 | 8.39E-09 | 0.006066 |
| RNF185 | cg24245352 | hsa.mir.3185 | 8.63E-09 | 0.006241 |
| FNDC3A* | cg06474453 | hsa.mir.1303 | 1.05E-08 | 0.007603 |
| TXNRD1* | cg08065092 | hsa.mir.3611 | 1.06E-08 | 0.007673 |
| CDKAL1 | cg07905141 | hsa.mir.3929 | 1.07E-08 | 0.00773 |
| TXNL1 | cg09130402 | hsa.mir.588 | 1.08E-08 | 0.007814 |
| TMED8 | cg14875498 | hsa.mir.375 | 1.09E-08 | 0.007883 |
| ITPKB | cg20452738 | hsa.mir.133b | 1.12E-08 | 0.008073 |
| ABL1* | cg10228162 | hsa.mir.484 | 1.15E-08 | 0.008339 |
| ZNF48 | cg01304182 | hsa.mir.484 | 1.18E-08 | 0.00853 |
| ECHDC1 | cg13817182 | hsa.mir.3174 | 1.20E-08 | 0.008674 |
| TXNL1 | cg06765434 | hsa.mir.320e | 1.25E-08 | 0.009012 |
| CTNS* | cg17745562 | hsa.mir.1976 | 1.43E-08 | 0.010342 |
| NEK9 | cg09945151 | hsa.mir.940 | 1.55E-08 | 0.011233 |
| NEK9 | cg09944325 | hsa.mir.940 | 1.69E-08 | 0.012234 |
| SHPK | cg03861021 | hsa.mir.943 | 1.81E-08 | 0.013072 |
| ELAVL4 | cg25217583 | hsa.mir.578 | 1.81E-08 | 0.013077 |
| MYO1C | cg18594963 | hsa.mir.3170 | 1.88E-08 | 0.013593 |
| FAM83H* | cg06465371 | hsa.mir.3937 | 1.91E-08 | 0.013772 |
| CDKAL1 | cg00682125 | hsa.mir.3929 | 1.91E-08 | 0.013841 |
| ZNF555 | cg21355100 | hsa.mir.665 | 1.93E-08 | 0.013948 |
| ZNF48 | cg00711896 | hsa.mir.484 | 1.98E-08 | 0.014276 |
| FNDC3A* | cg06481158 | hsa.mir.1303 | 2.03E-08 | 0.014685 |
| E2F3 | cg21803390 | hsa.mir.607 | 2.10E-08 | 0.015188 |
| RNASEH2B | cg07818646 | hsa.mir.3929 | 2.16E-08 | 0.015636 |
| NEK9 | cg13494334 | hsa.mir.940 | 2.22E-08 | 0.016037 |
| FRS2* | cg04913666 | hsa.mir.3646 | 2.23E-08 | 0.016147 |
| ERBB2 | cg25582353 | hsa.mir.375 | 2.24E-08 | 0.016207 |
| E2F3 | cg21803390 | hsa.mir.1260b | 2.48E-08 | 0.017919 |
| SEC16A | cg00261144 | hsa.mir.484 | 2.54E-08 | 0.018378 |
| CAMKK2 | cg04615865 | hsa.mir.3198 | 2.61E-08 | 0.018885 |
| SLC35E3 | cg15993521 | hsa.mir.665 | 2.65E-08 | 0.019151 |
| DGKE* | cg10654010 | hsa.mir.1267 | 2.65E-08 | 0.019185 |
| MYO1C | cg13588265 | hsa.mir.3198 | 2.70E-08 | 0.01955 |
| AKT2 | cg26862316 | hsa.mir.137 | 2.71E-08 | 0.019566 |
| ERLIN2 | cg09056309 | hsa.mir.326 | 2.72E-08 | 0.019643 |
| CDADC1 | cg18587973 | hsa.mir.107 | 2.72E-08 | 0.019675 |
| TPCN2 | cg20256574 | hsa.mir.449a | 3.13E-08 | 0.022595 |
| BCL3 | cg17883035 | hsa.mir.3115 | 3.19E-08 | 0.023071 |
| BCL3 | cg26256192 | hsa.mir.3115 | 3.46E-08 | 0.024973 |
| NEK9 | cg14426084 | hsa.mir.940 | 3.55E-08 | 0.025668 |
| NIPBL | cg08374525 | hsa.mir.3936 | 3.69E-08 | 0.02664 |
| CAMKK2 | cg22032709 | hsa.mir.3198 | 3.69E-08 | 0.026661 |
| SRRM4* | cg11088135 | hsa.mir.3167 | 3.85E-08 | 0.027837 |
| FAM83H* | cg26039042 | hsa.mir.3937 | 3.93E-08 | 0.028423 |
| TACR2 | cg23339629 | hsa.mir.1976 | 4.16E-08 | 0.030084 |
| RIMS4 | cg12343182 | hsa.mir.4254 | 4.42E-08 | 0.031977 |
| PPM1K* | cg17127050 | hsa.mir.1976 | 4.85E-08 | 0.035042 |
| MRPL36* | cg26009970 | hsa.mir.555 | 4.88E-08 | 0.035296 |
| ELAVL4 | cg26301690 | hsa.mir.375 | 4.90E-08 | 0.03544 |
| KLHL26* | cg16705777 | hsa.mir.484 | 5.03E-08 | 0.036338 |
| TBCCD1* | cg13031679 | hsa.mir.564 | 5.10E-08 | 0.036898 |
| RAB11FIP3 | cg10357909 | hsa.mir.484 | 5.15E-08 | 0.037254 |
| CAND1* | cg02063925 | hsa.mir.548v | 5.42E-08 | 0.039154 |
| RNF185 | cg12504284 | hsa.mir.3185 | 6.03E-08 | 0.0436 |
| KLHDC8A* | cg13459217 | hsa.mir.640 | 6.13E-08 | 0.044284 |
| MYO1C | cg08464498 | hsa.mir.3170 | 6.15E-08 | 0.044436 |
| CHRNB2* | cg13393009 | hsa.mir.1291 | 6.20E-08 | 0.044826 |
| ELAVL4 | cg25217583 | hsa.mir.375 | 6.21E-08 | 0.044866 |
| SLC12A7 | cg02350636 | hsa.mir.1291 | 6.24E-08 | 0.04511 |
| TBX3 | cg13930596 | hsa.mir.137 | 6.60E-08 | 0.047676 |
| DDHD1 | cg13654573 | hsa.mir.934 | 6.72E-08 | 0.048585 |
| USP42* | cg04734175 | hsa.mir.3686 | 6.91E-08 | 0.049966 |

* Non-papillary subtype-specific genes compared to the papillary and all patient groups

**Table S4.** Summary of overall survival analysis results.

| **Gene** | **Methylation** | **miRNA** | **Bonferroni-corrected LRT p-value** | **Permutation p-value** |
| --- | --- | --- | --- | --- |
| E2F3 | cg21803390 | hsa.mir.217 | 8.31E-08 | 0.001 |
| CCND1 | cg03040489 | hsa.mir.944 | 3.81E-05 | 0.001 |
| UTP6 | cg13453082 | hsa.mir.1254 | 2.44E-04 | 0.001 |
| CDADC1 | cg17226947 | hsa.mir.107 | 7.37E-04 | 0.002 |
| SLC35E3 | cg02006977 | hsa.mir.940 | 1.08E-03 | 0.001 |
| METRNL | cg01502876 | hsa.mir.107 | 1.49E-03 | 0.001 |
| TPCN2 | cg10490196 | hsa.mir.1976 | 1.69E-03 | 0.001 |
| NACC2 | cg13979562 | hsa.mir.107 | 1.92E-03 | 0.001 |
| VGLL4 | cg25619837 | hsa.mir.3662 | 9.72E-03 | 0.001 |
| METRNL | cg03155999 | hsa.mir.107 | 1.15E-02 | 0.001 |
| PTEN | cg166686761 | hsa.mir.543 | 4.97E-02 | 0.001 |
| E2F3 | cg21803390 | hsa.mir.217 | 8.31E-08 | 0.001 |

**Figure S1.** Venn Diagram of Significant target genes for papillary, non-papillary subtypes.**
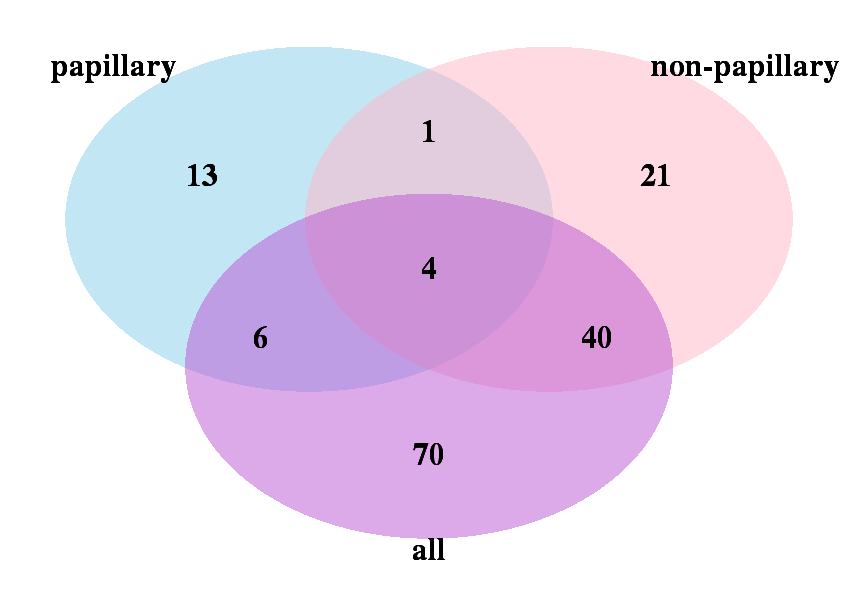
**

**Figure S2.** Survival analysis between two subgroups (LL and HH)

**
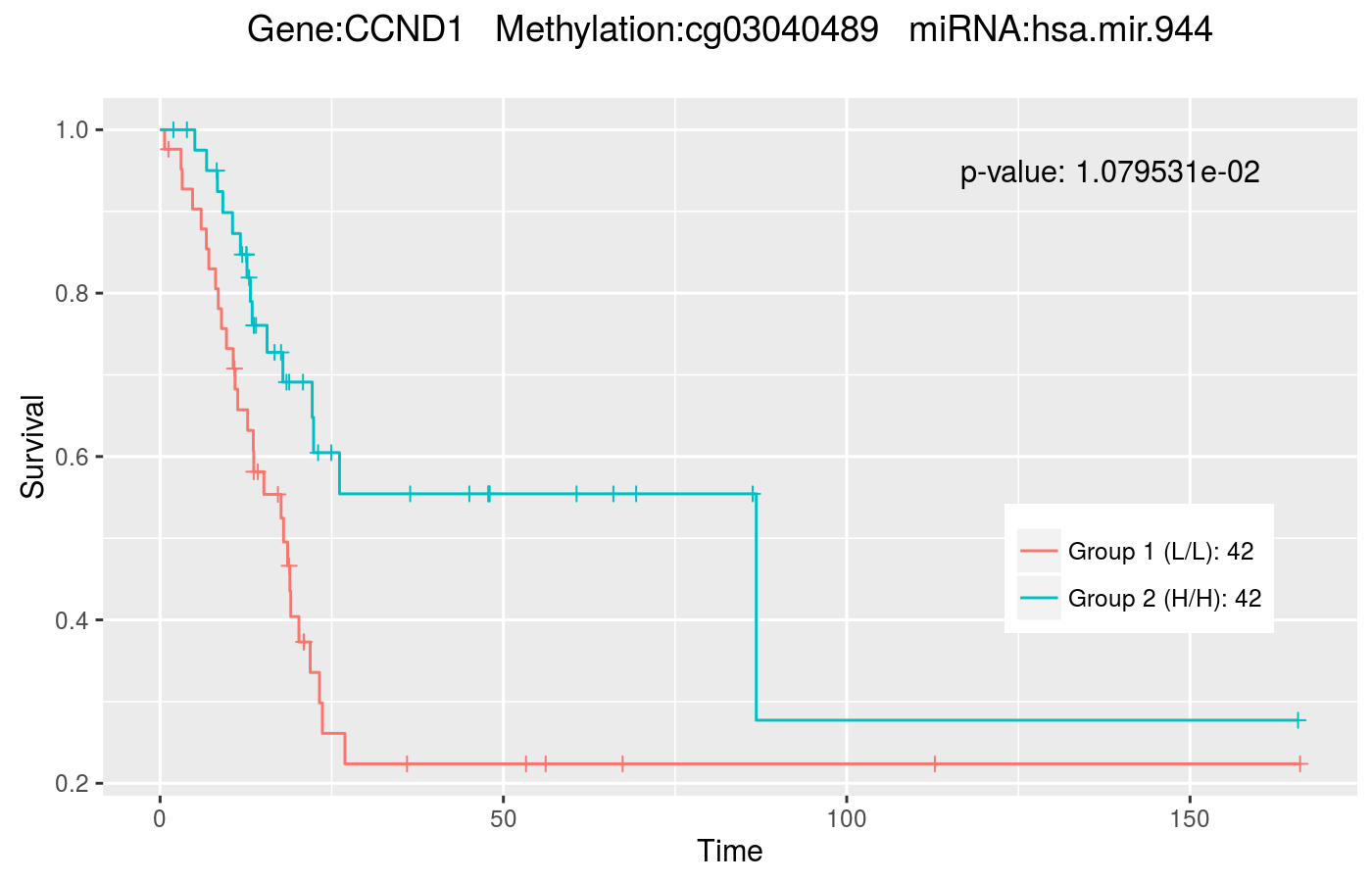

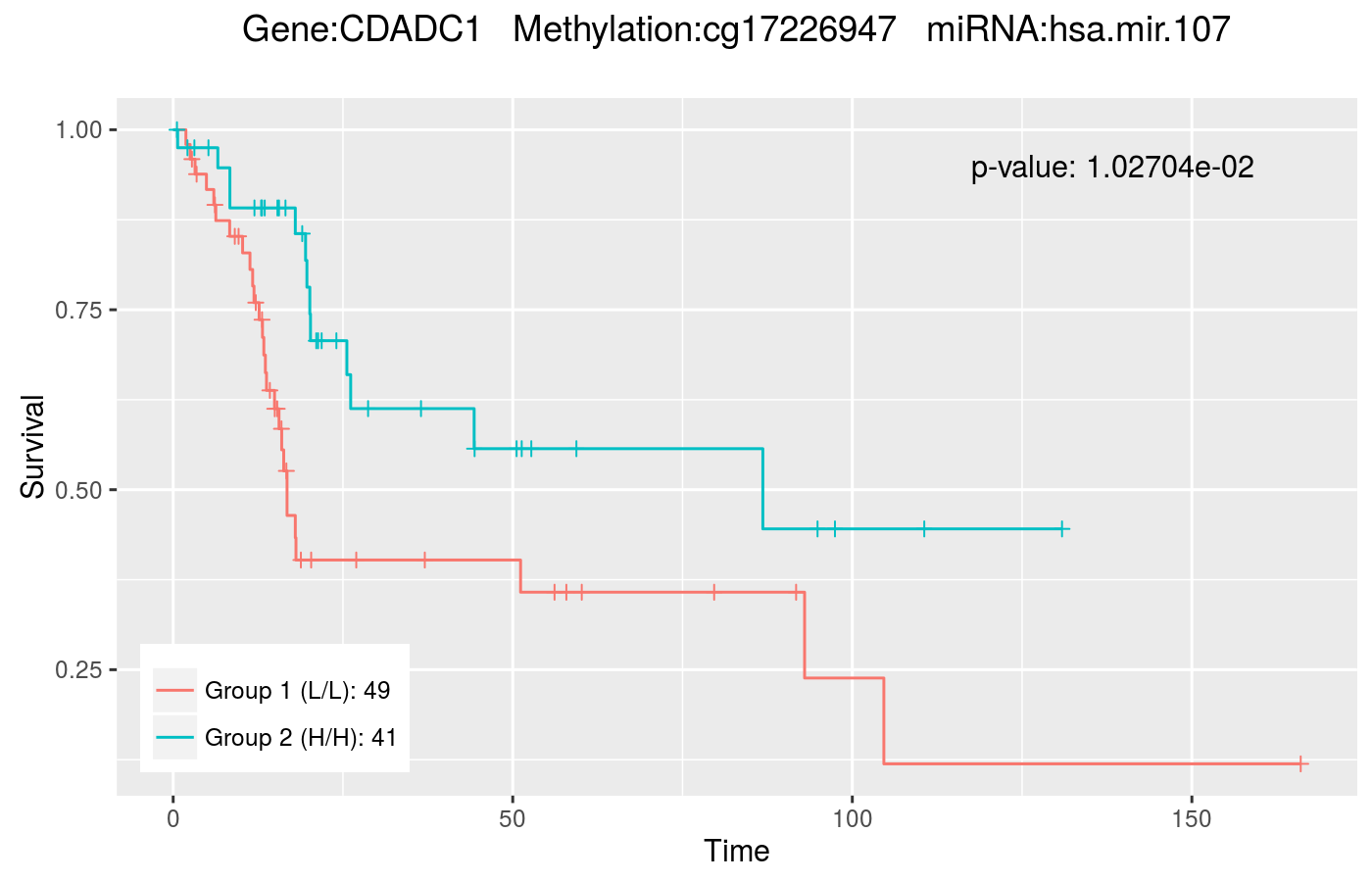

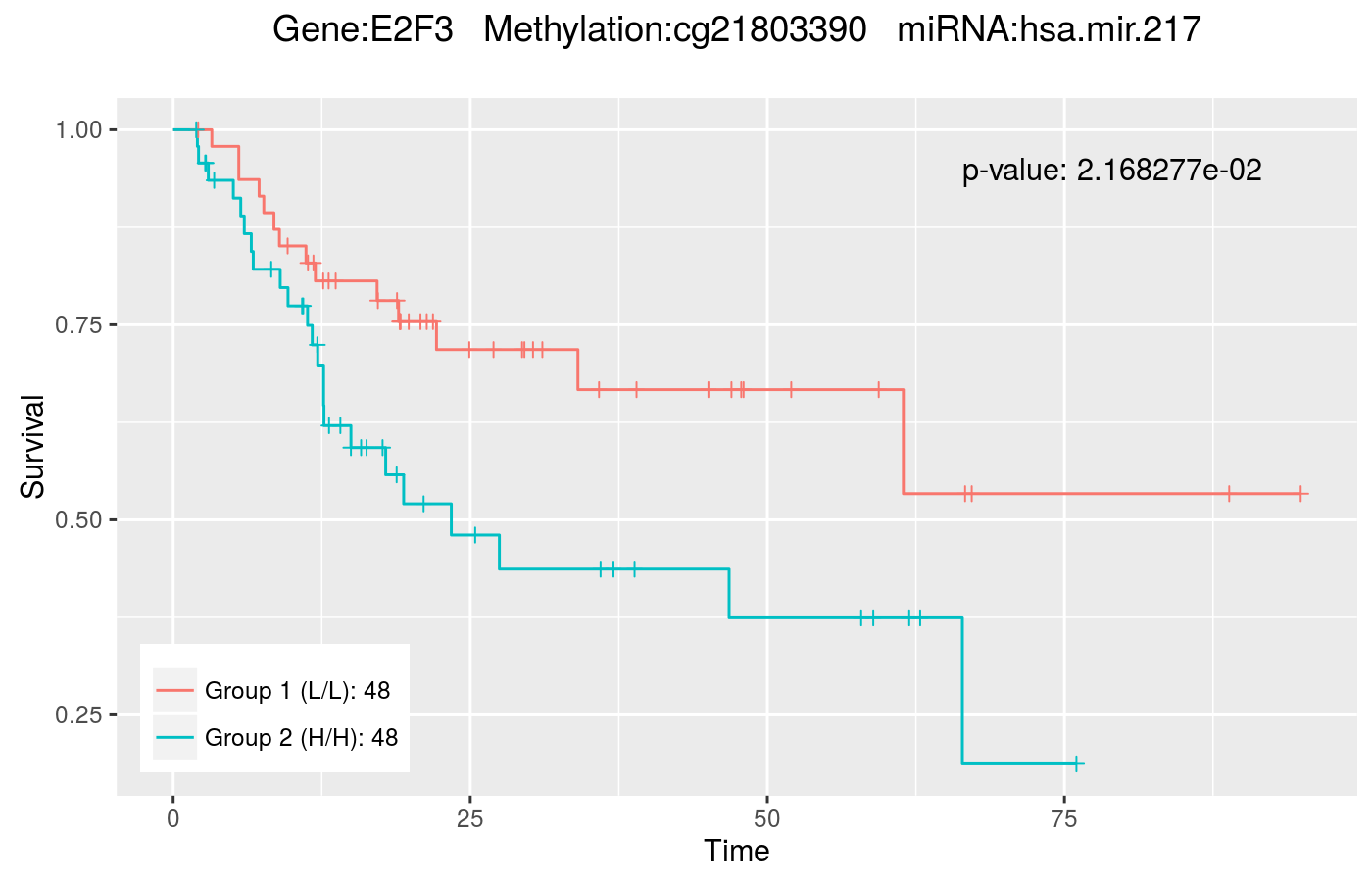

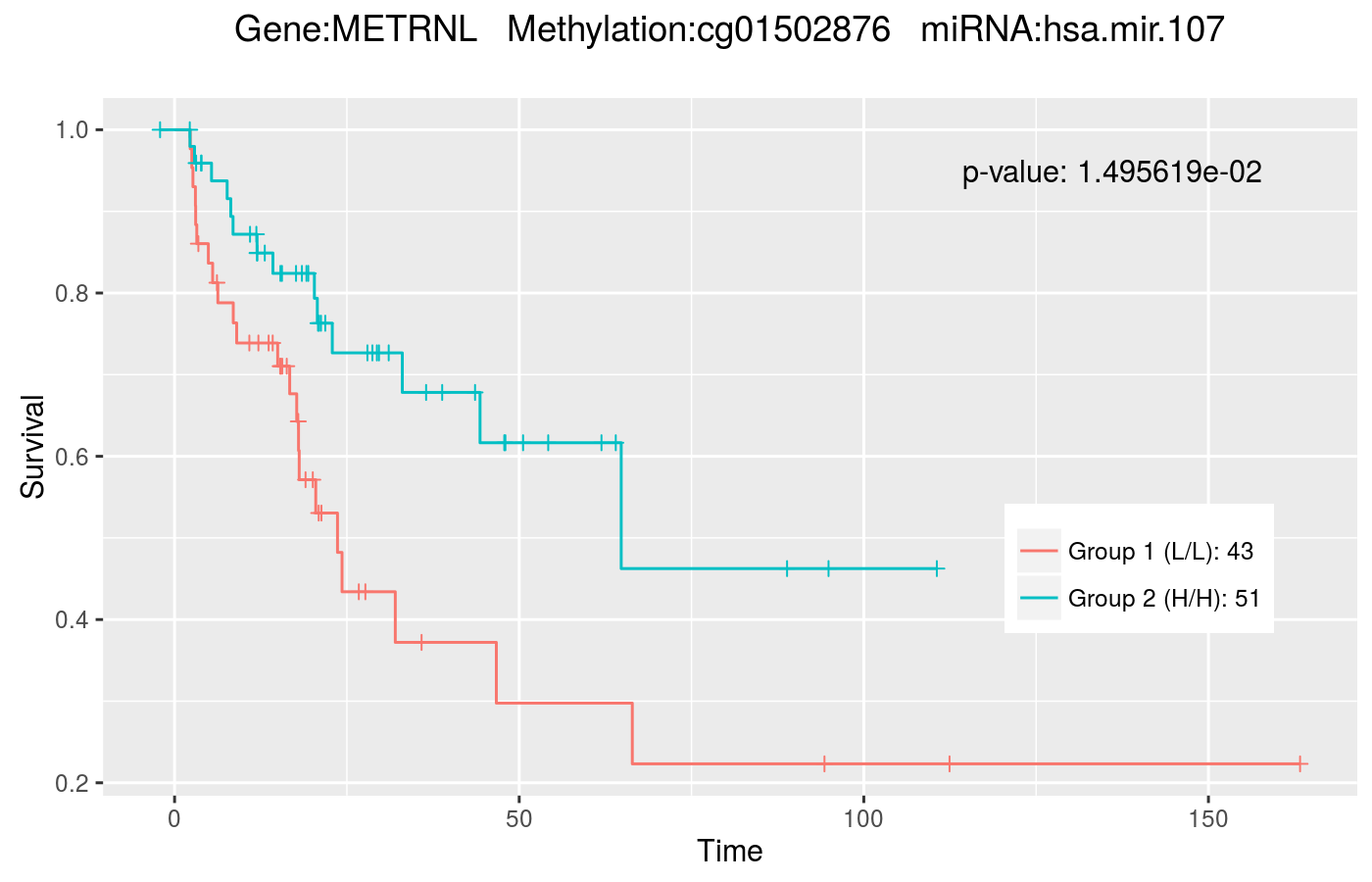

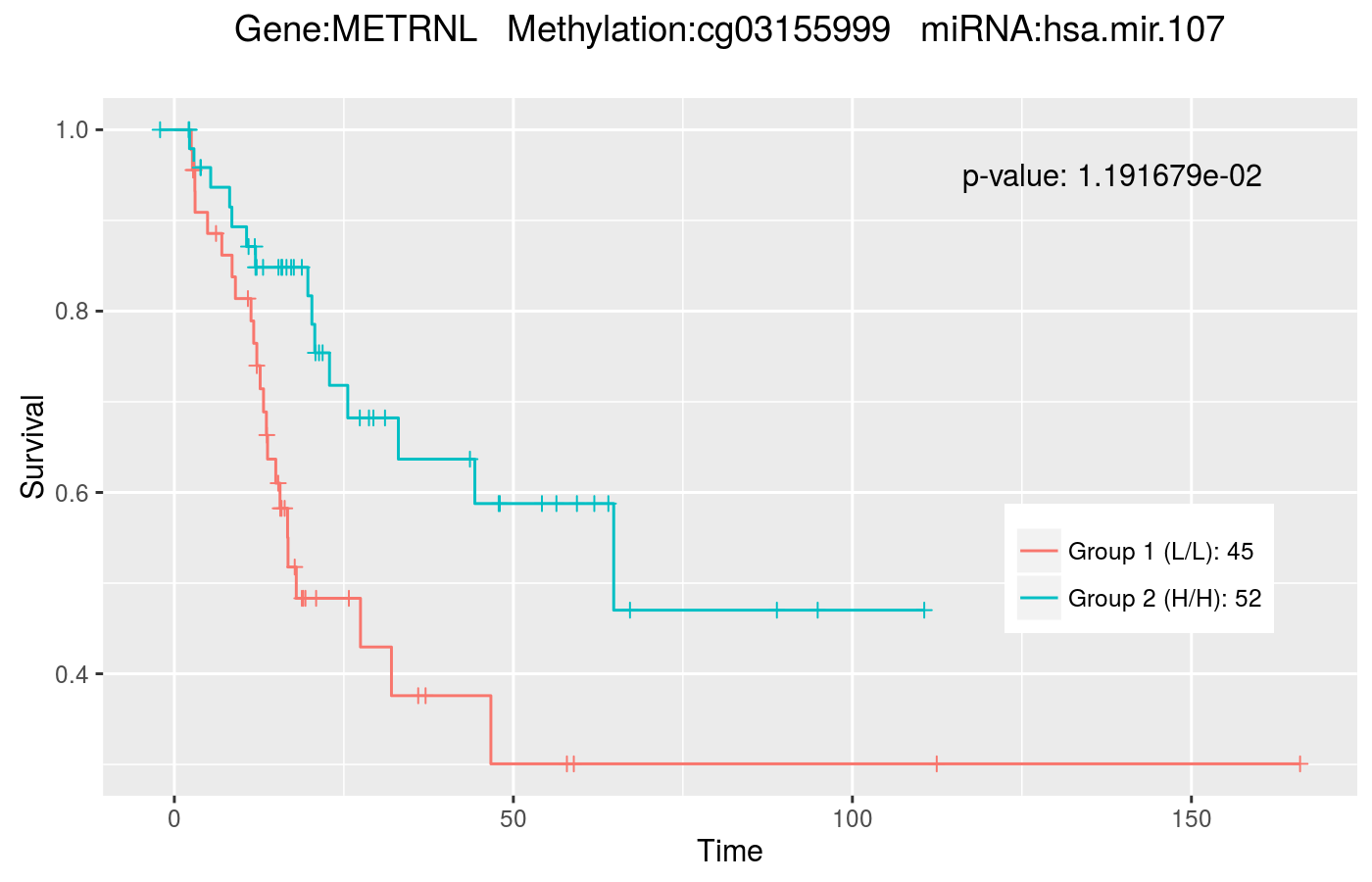

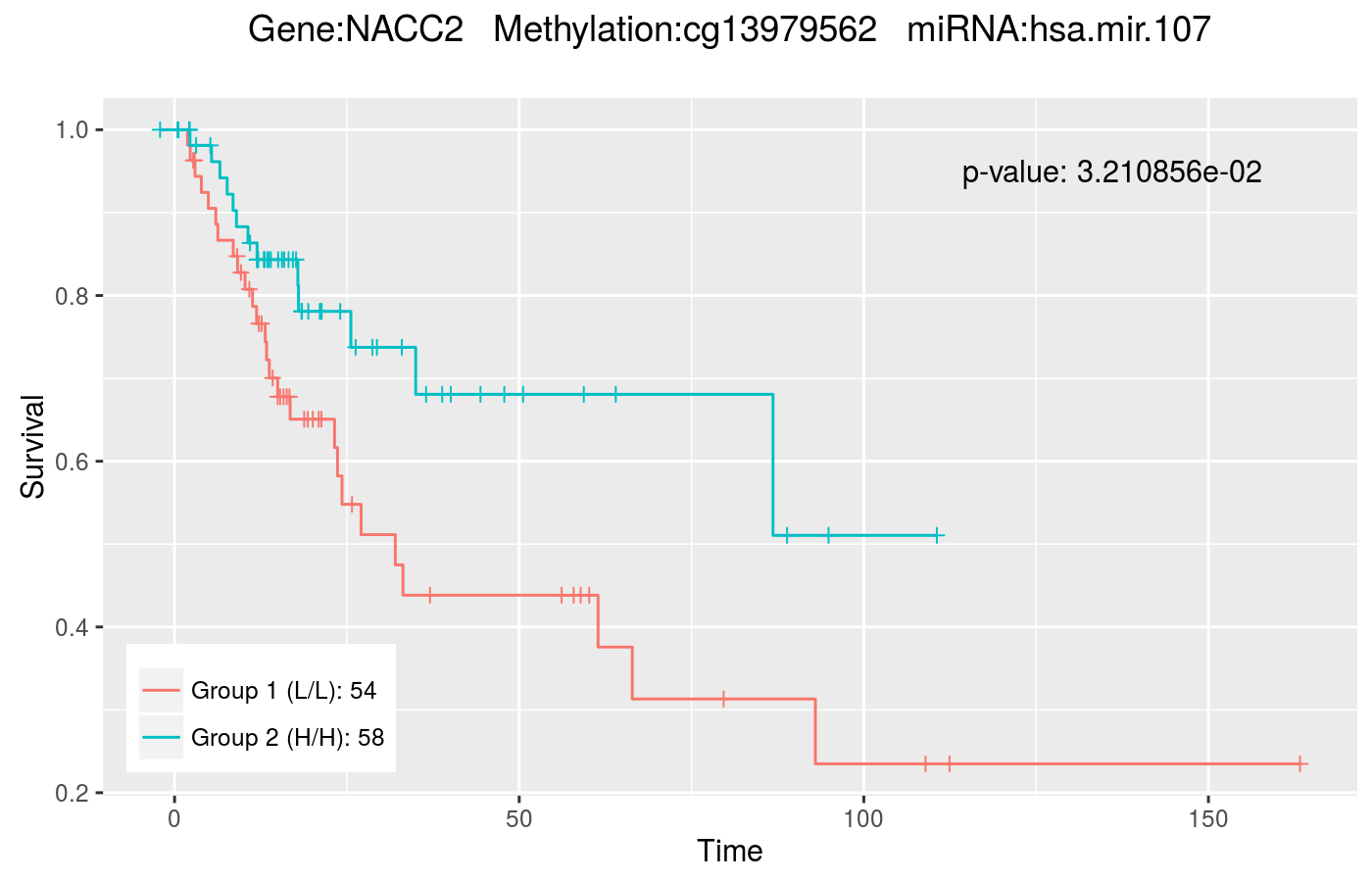

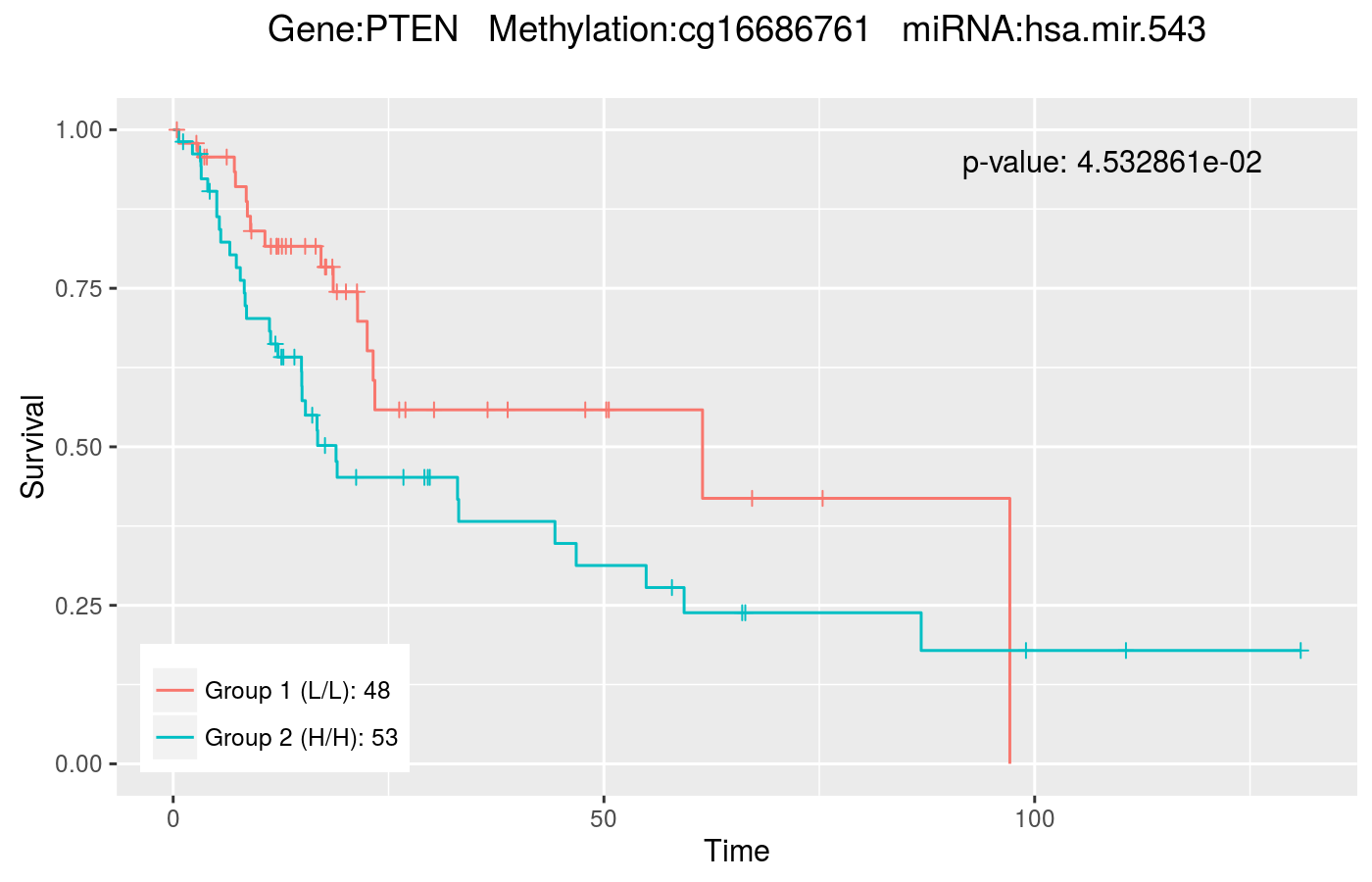

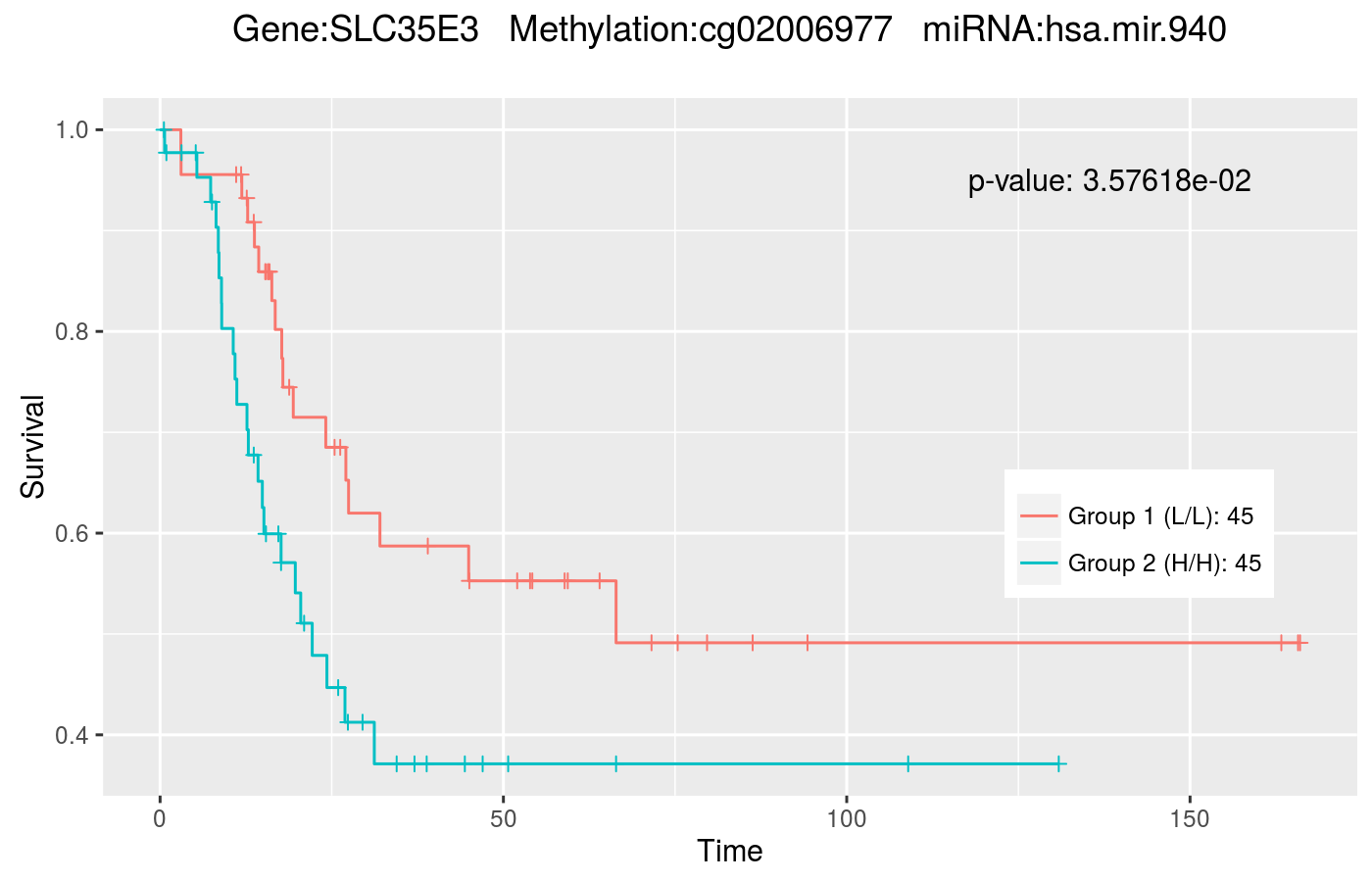

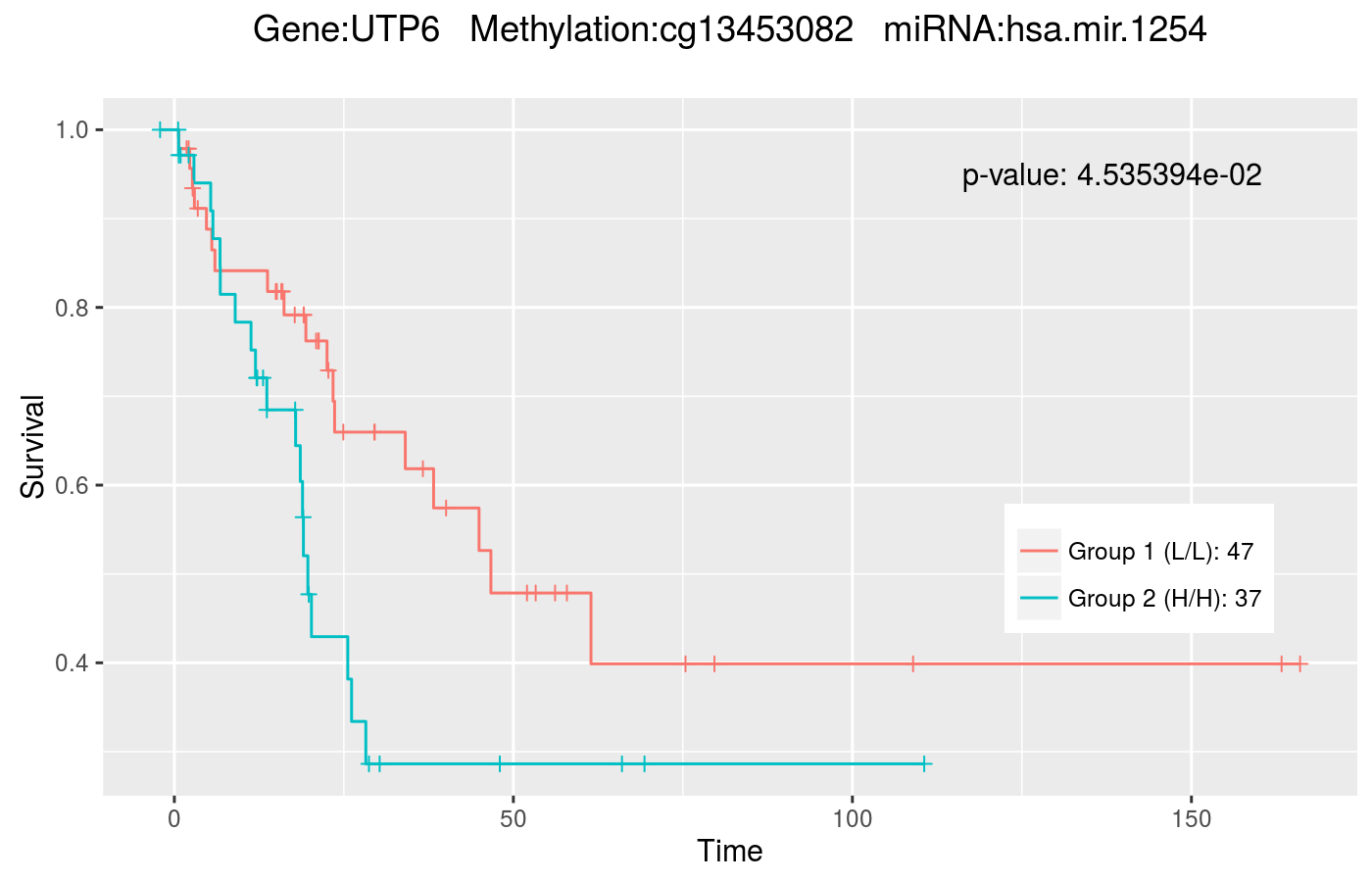

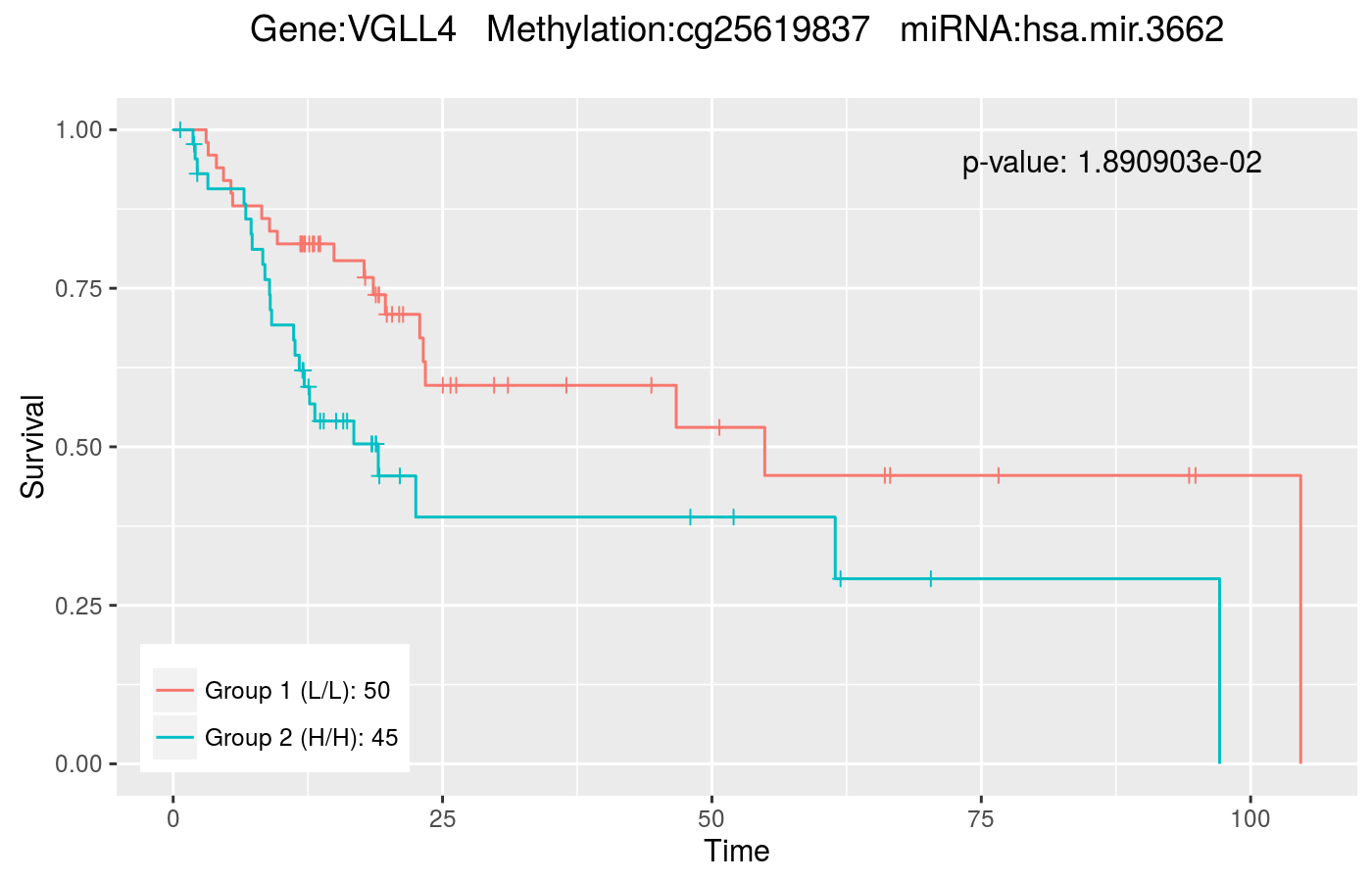
**
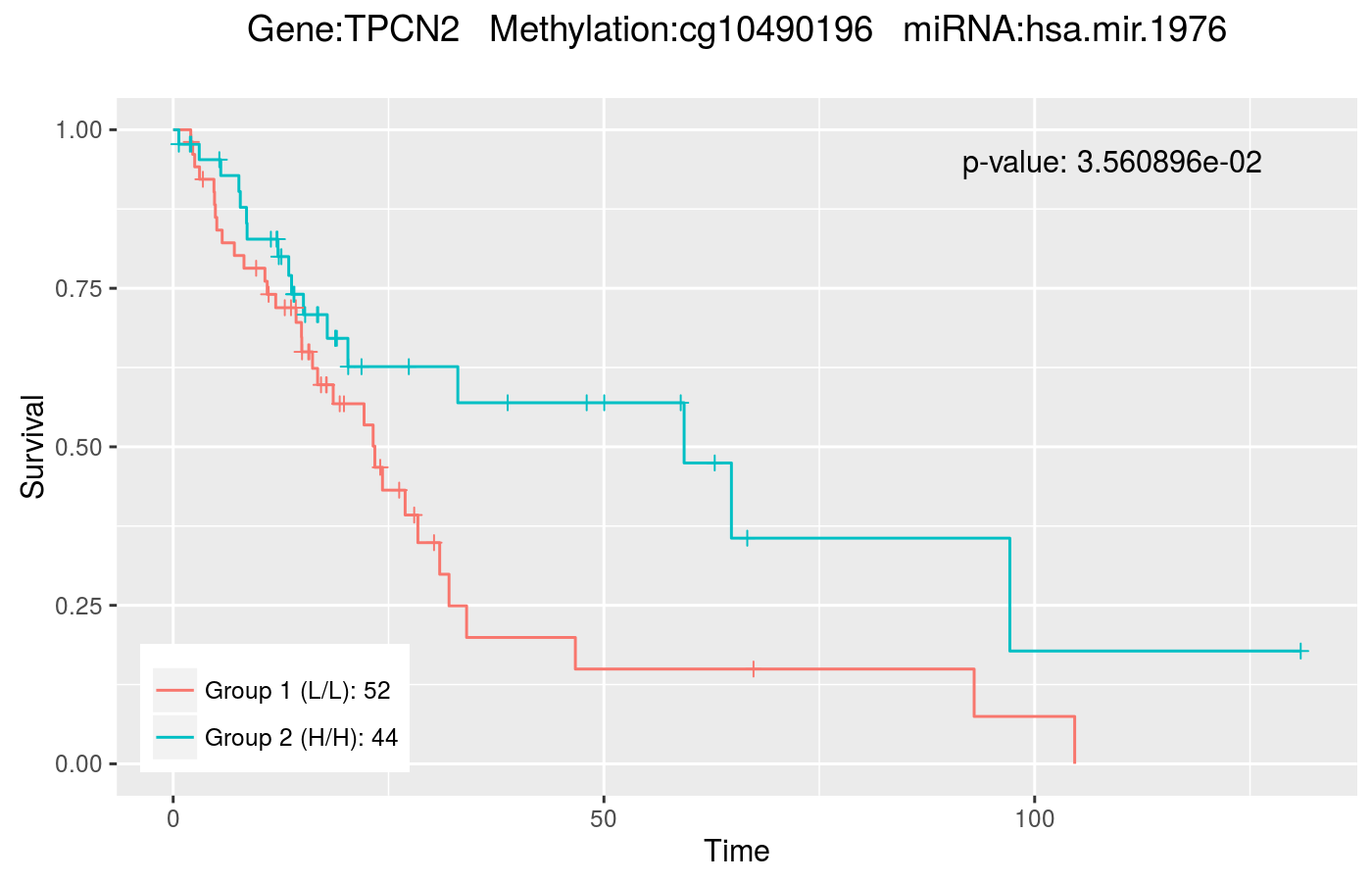


**Figure S3.** Gene expression boxplot for two subgroups (LL and HH)

**
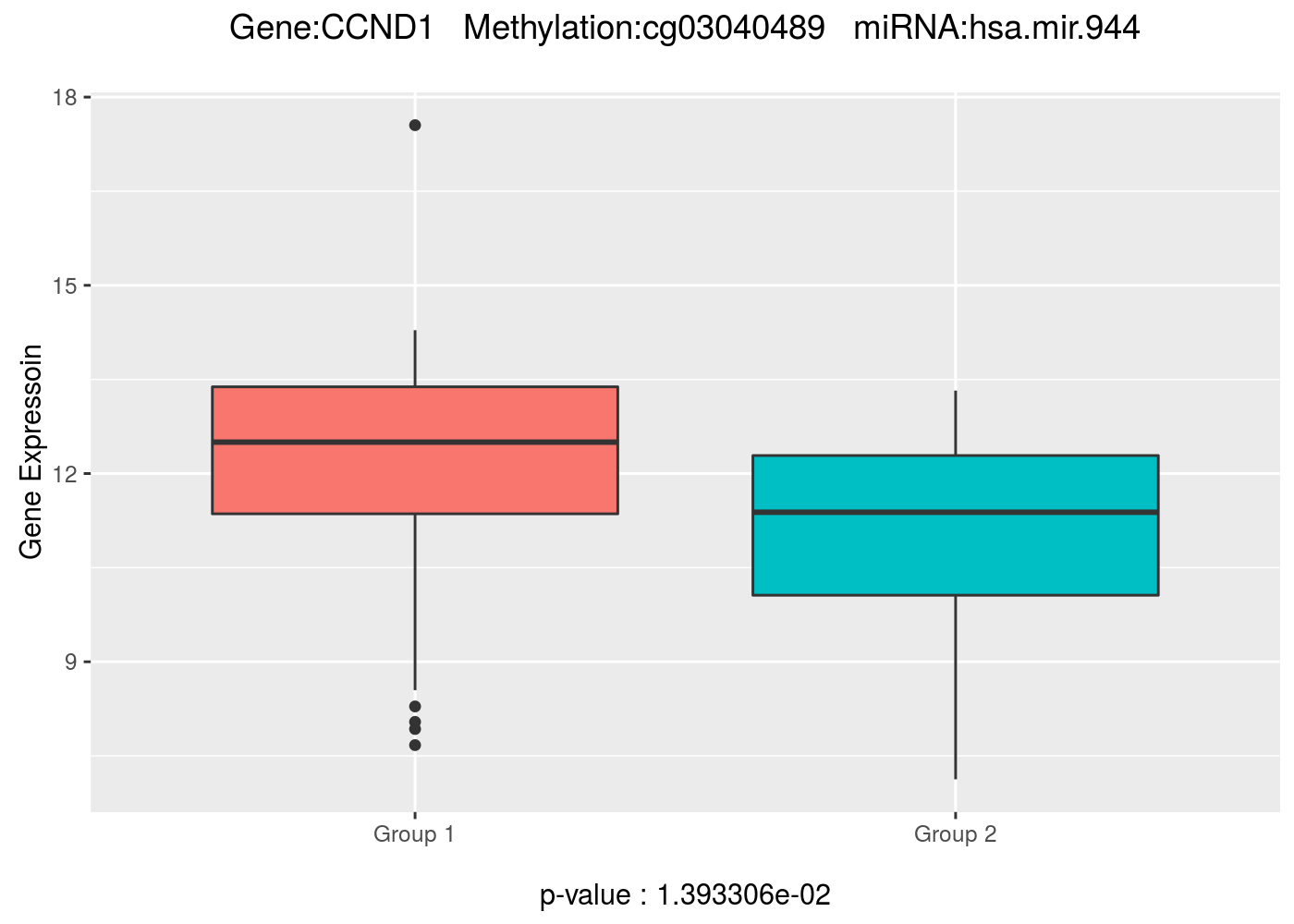

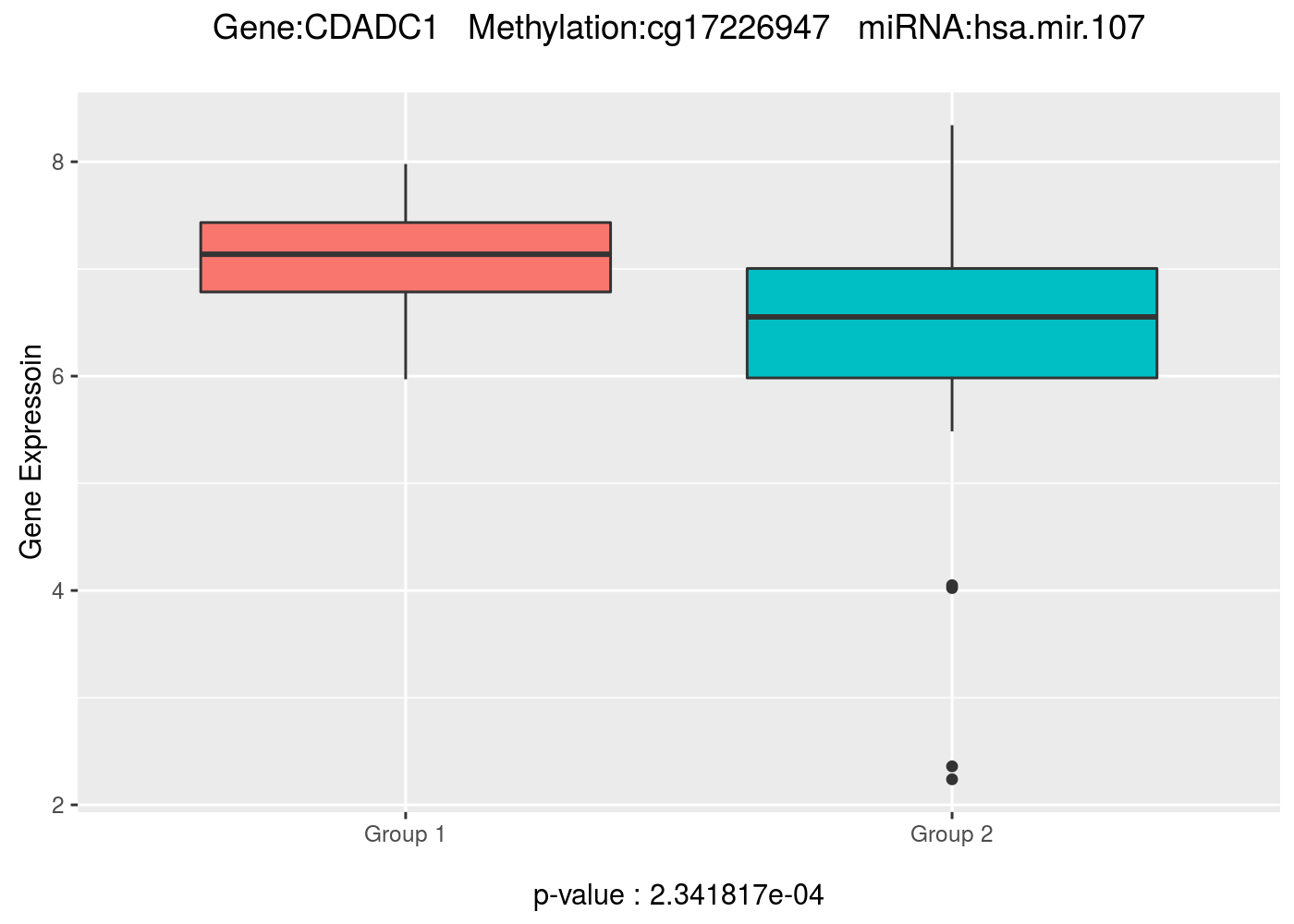

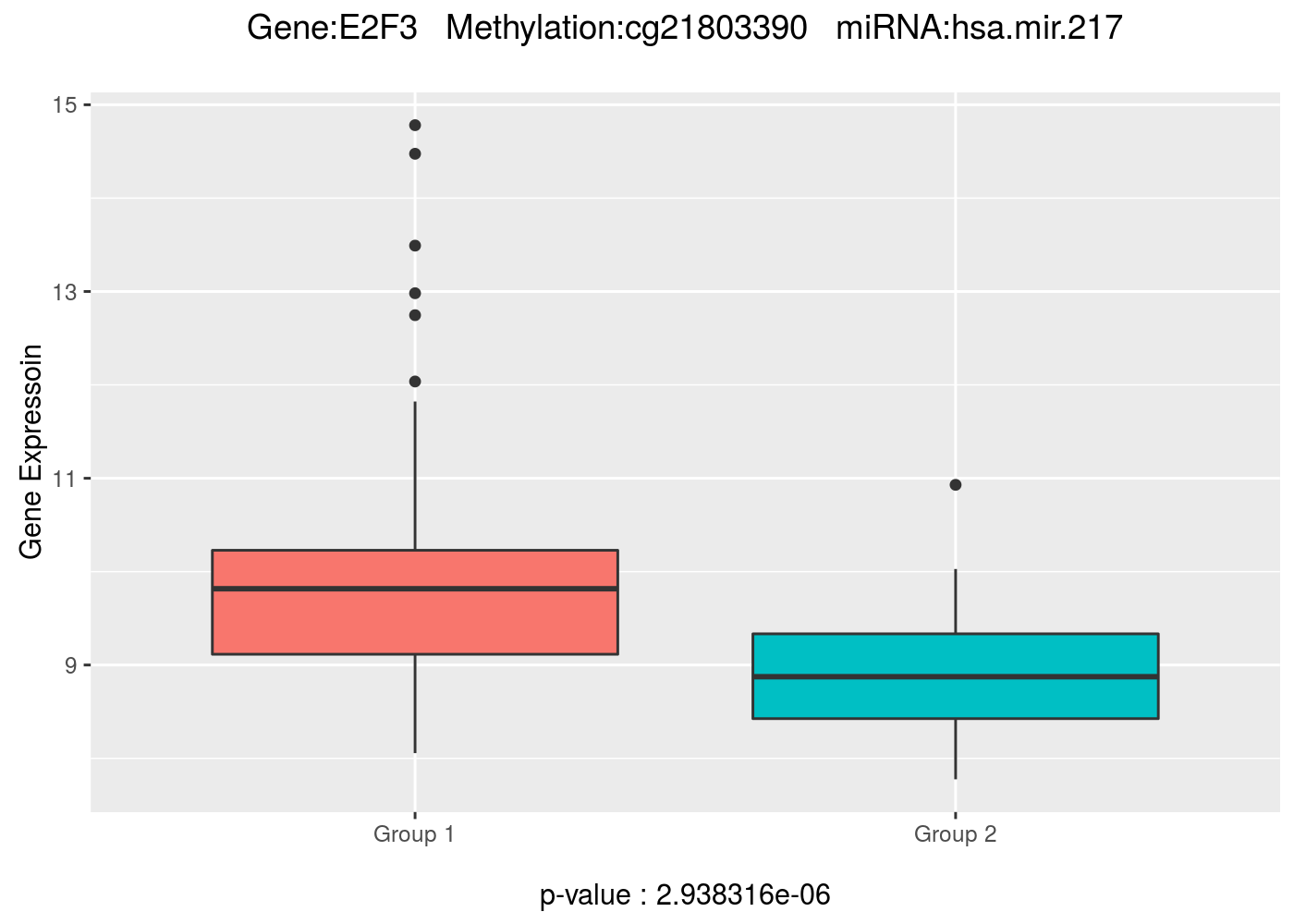

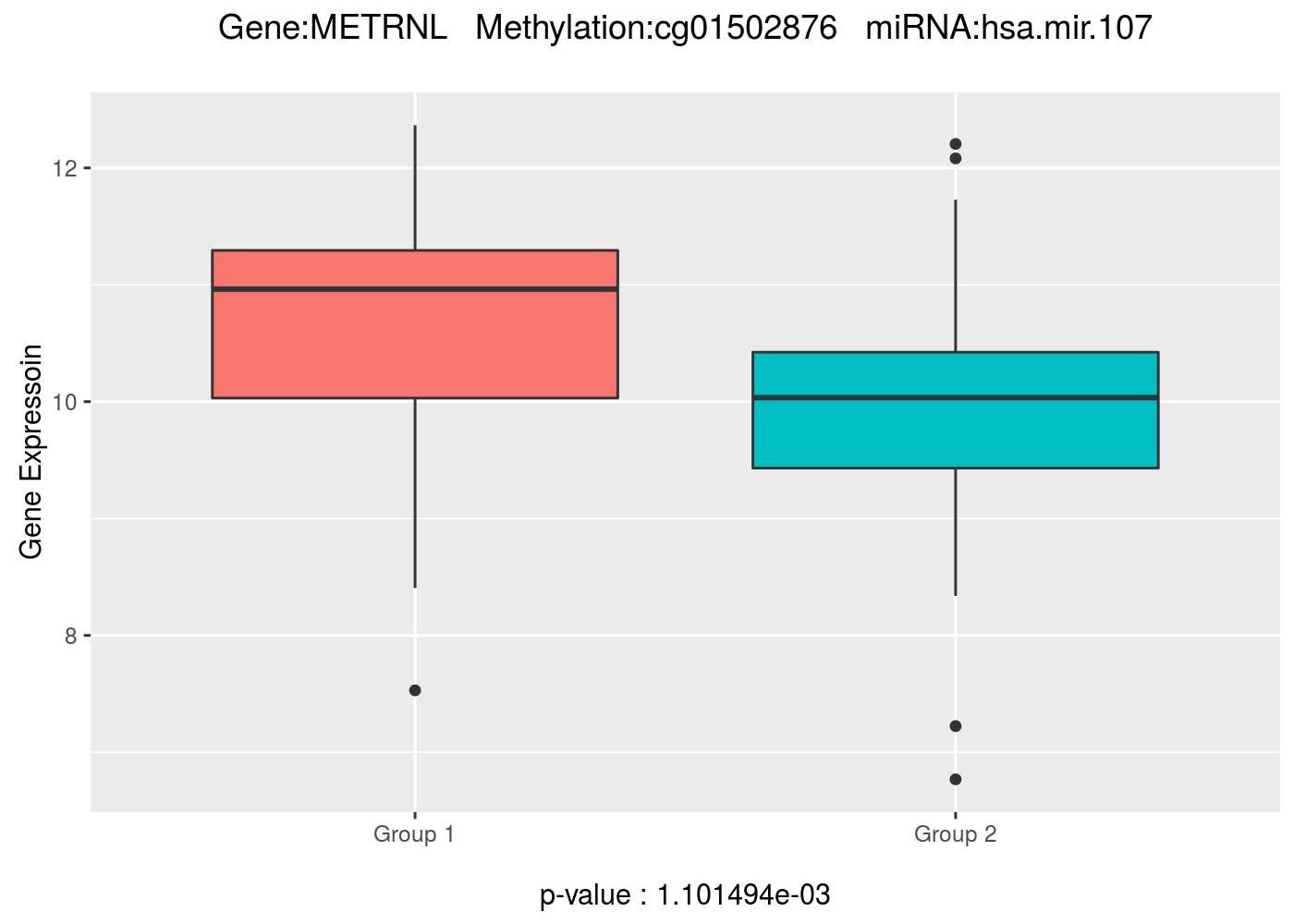

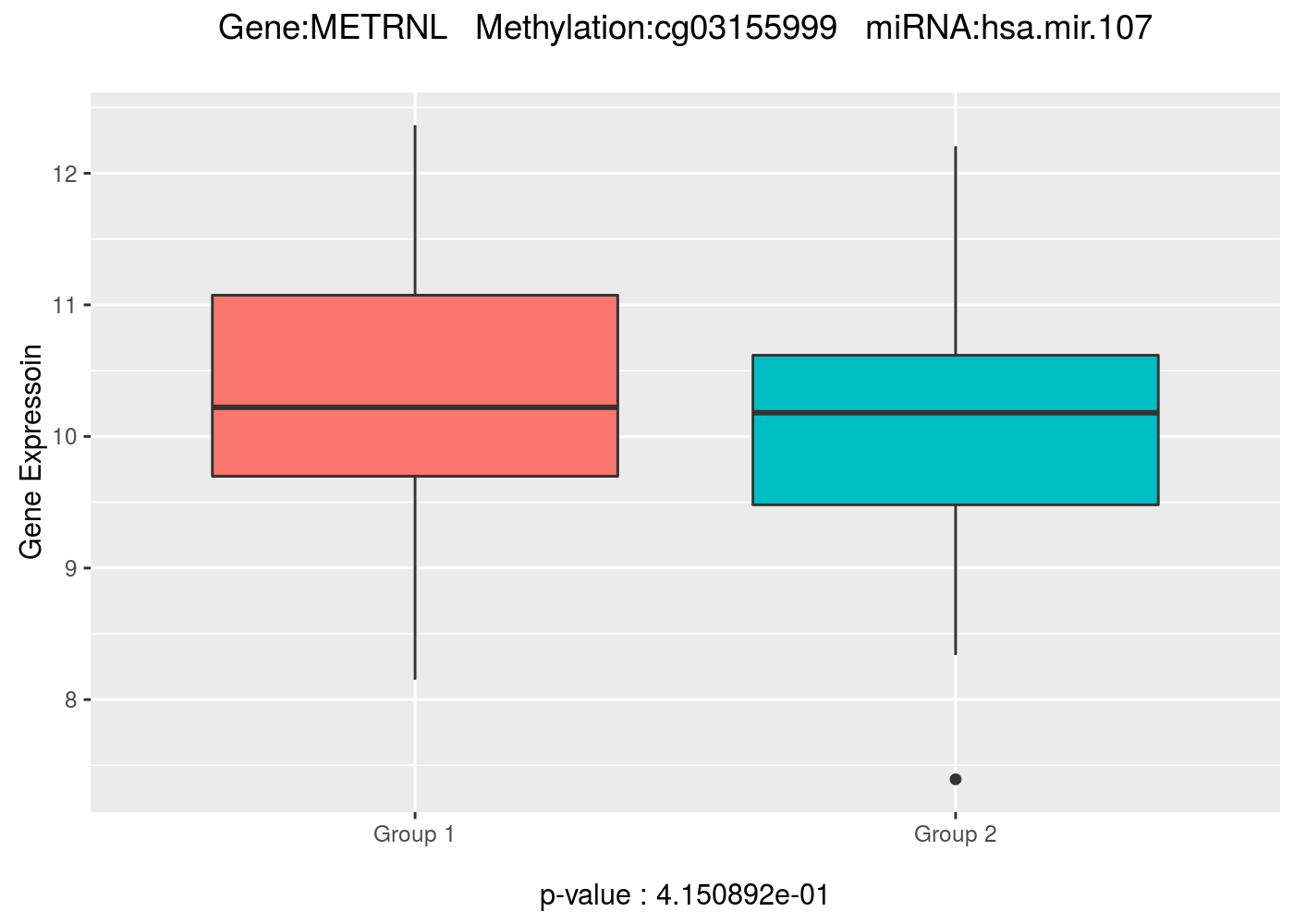

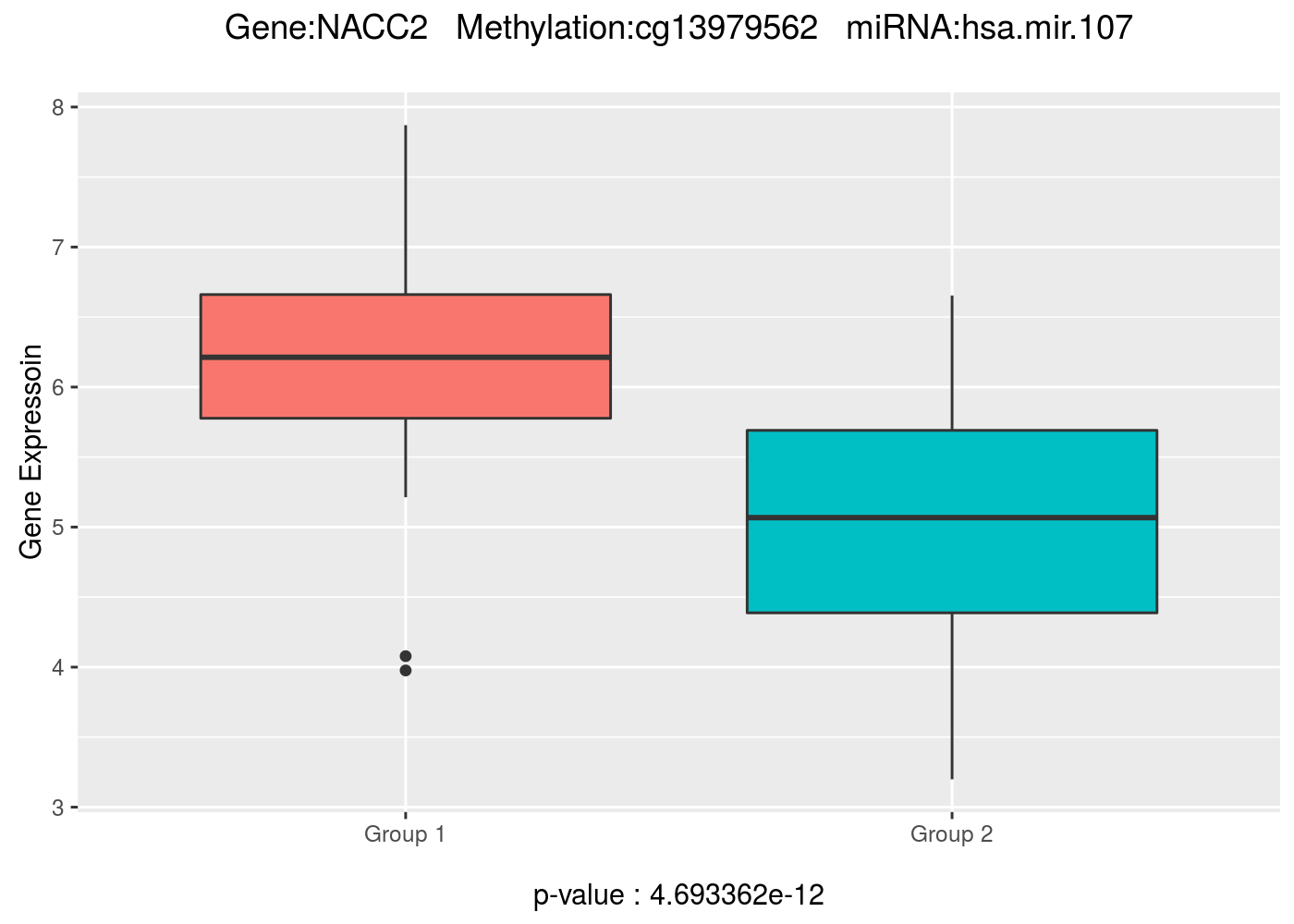

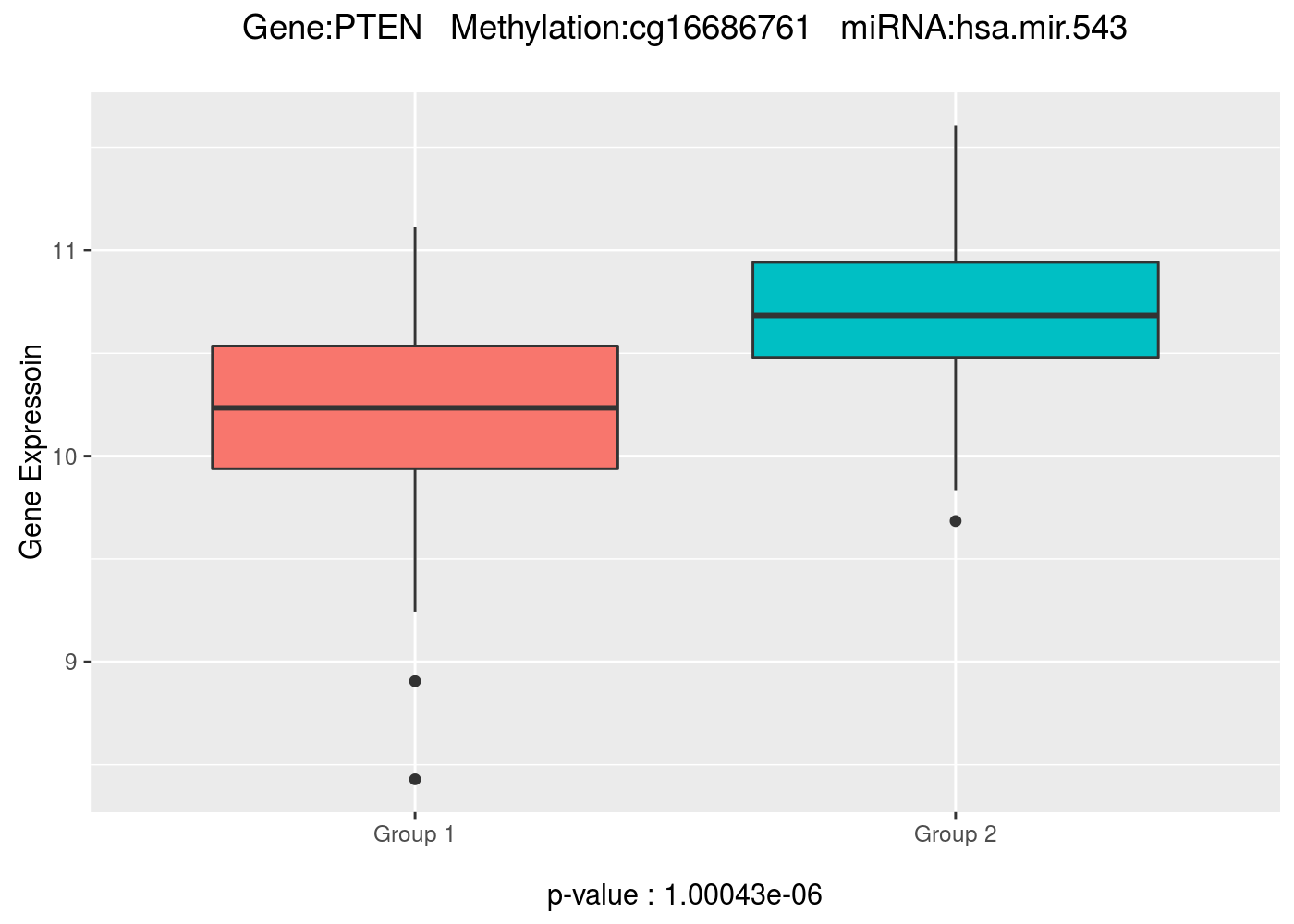

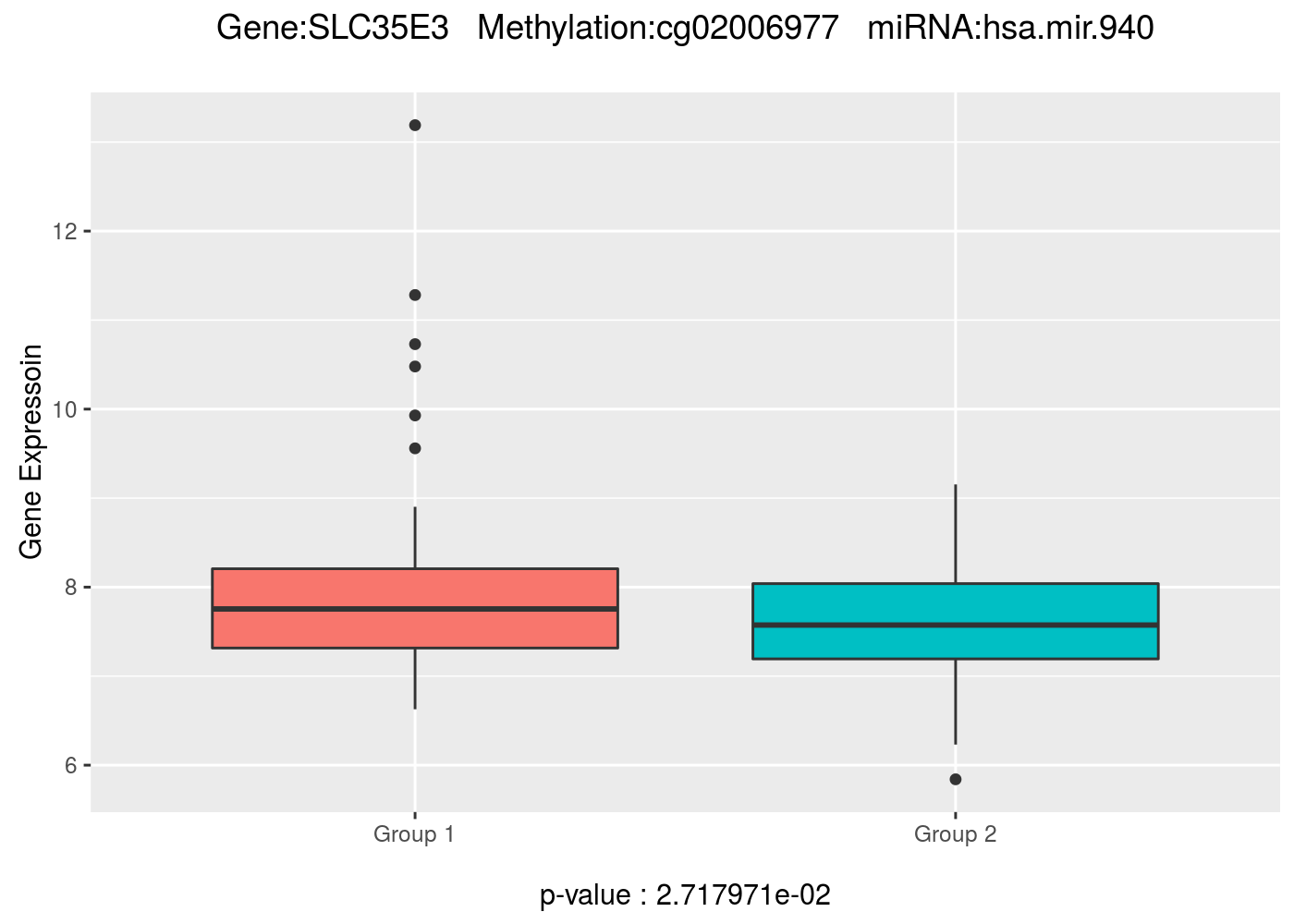

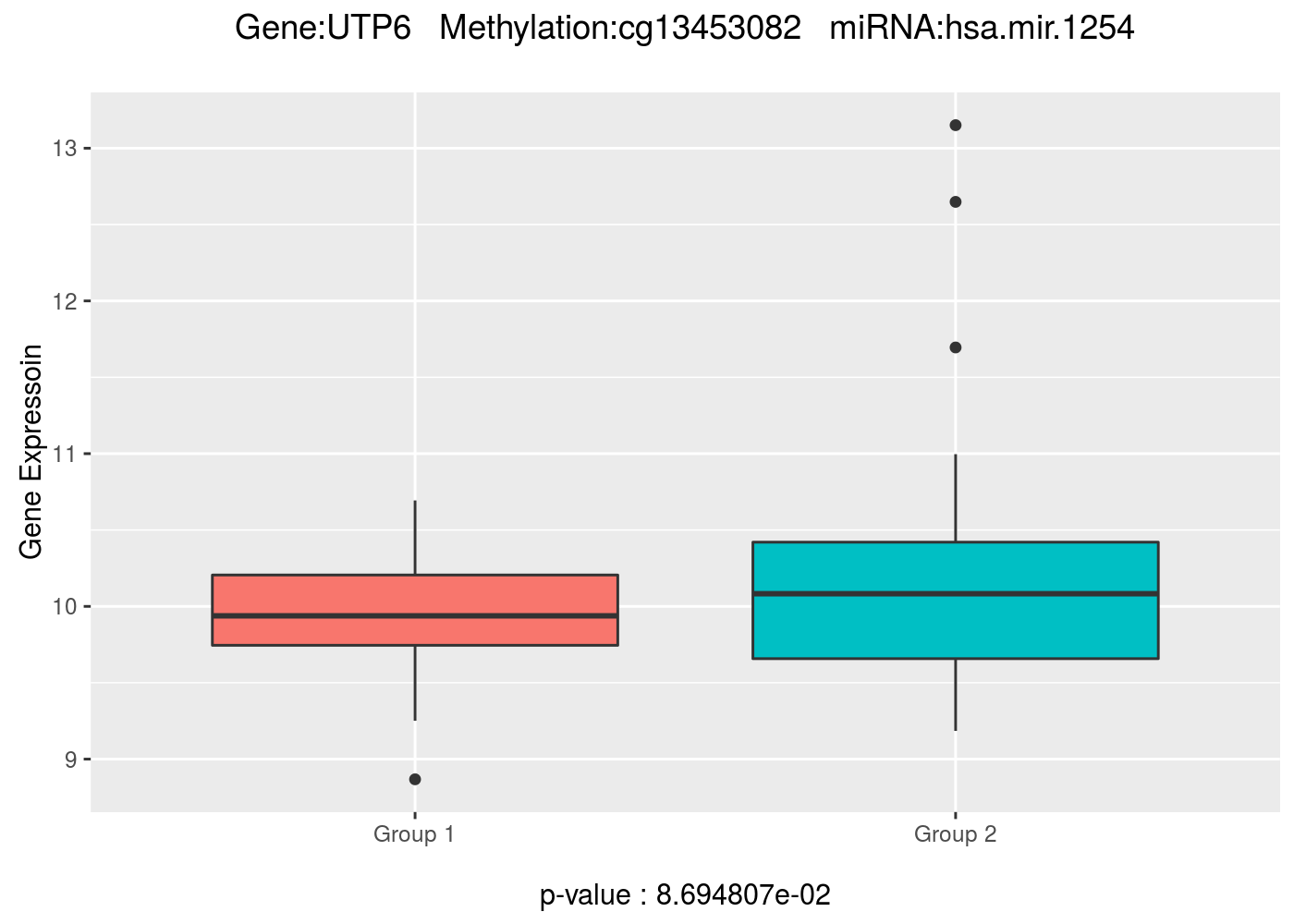

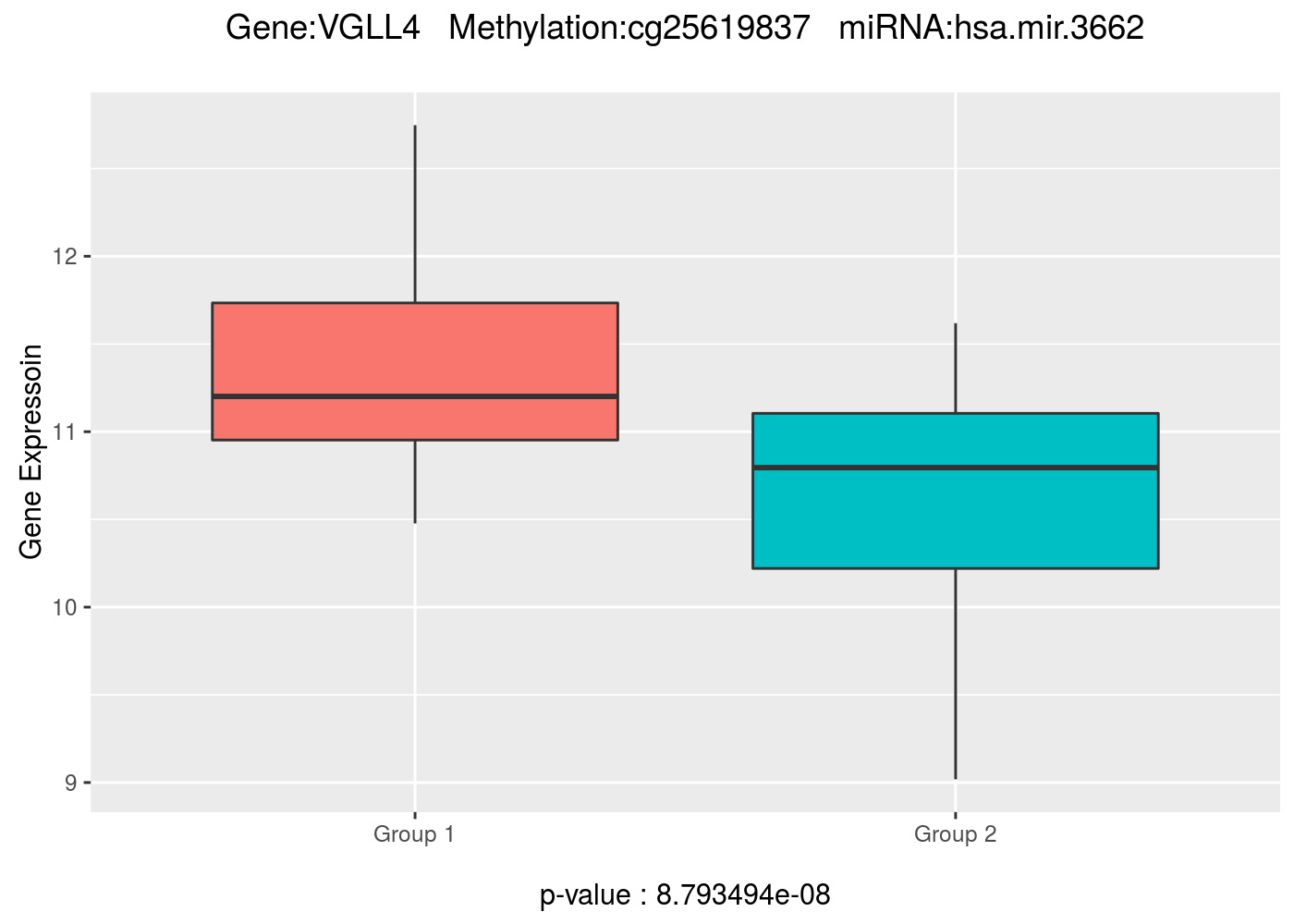
**
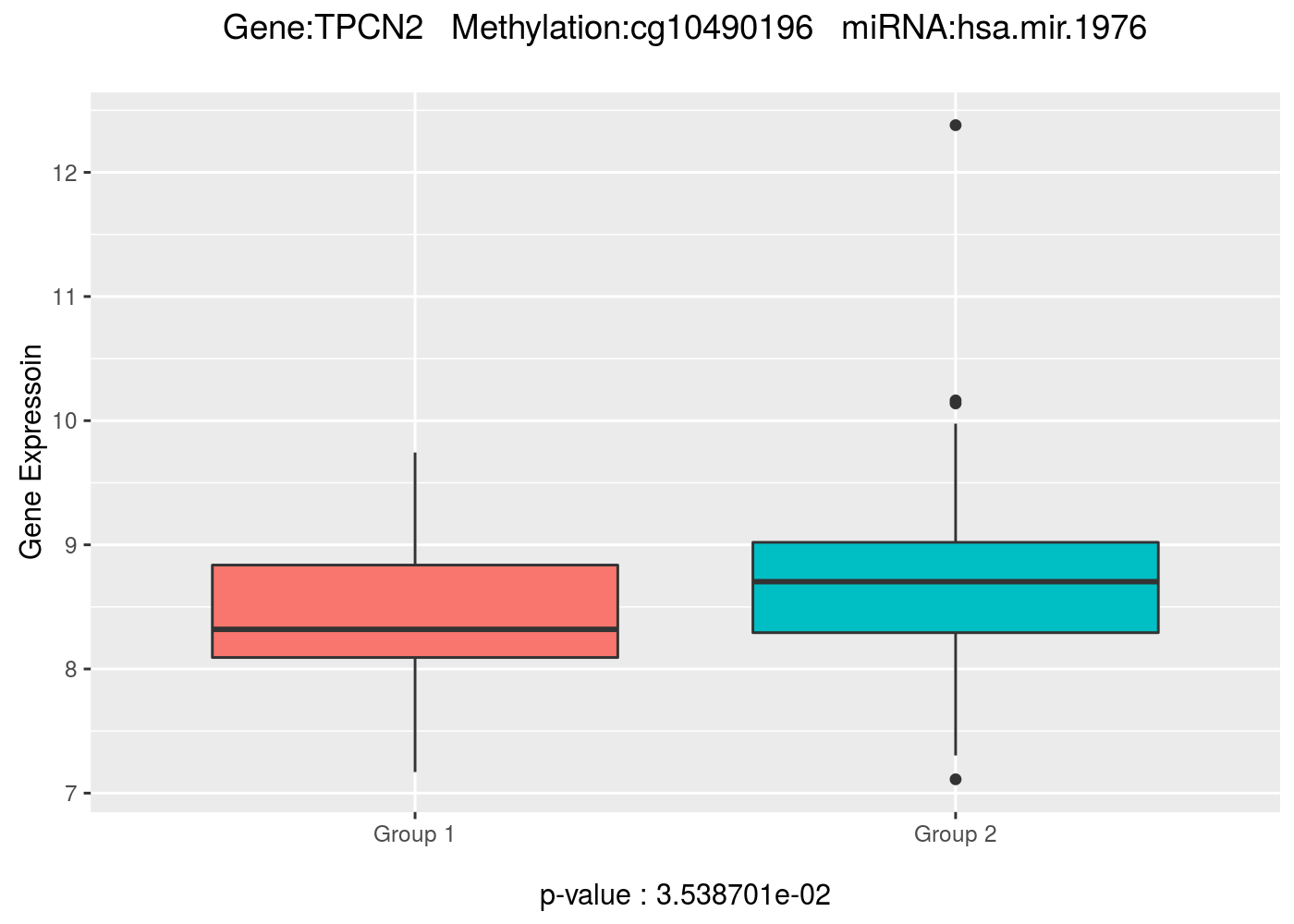


**Figure S4.** Gene expression boxplot for four subgroups (LL, LH, HL, and HH)

**
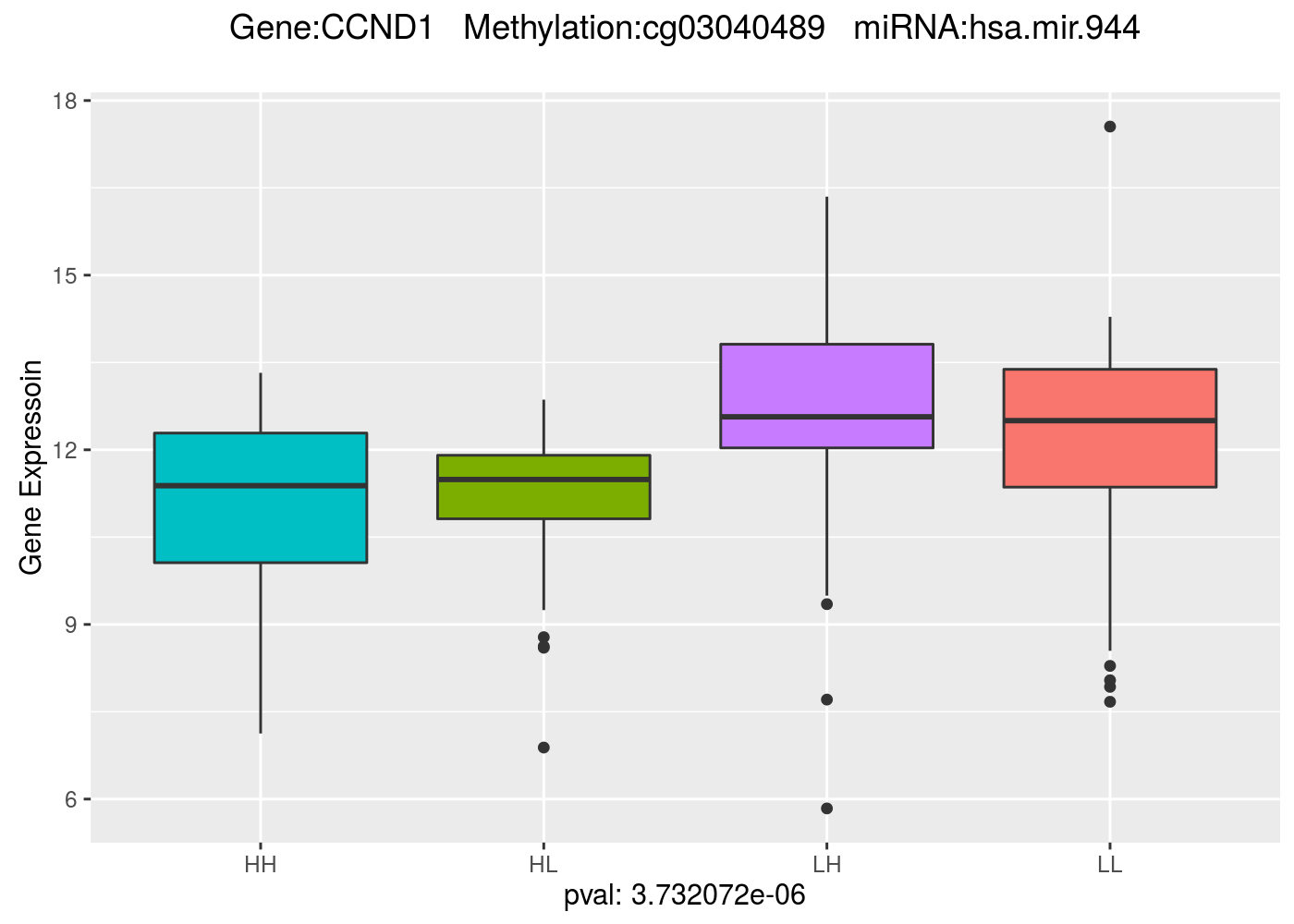

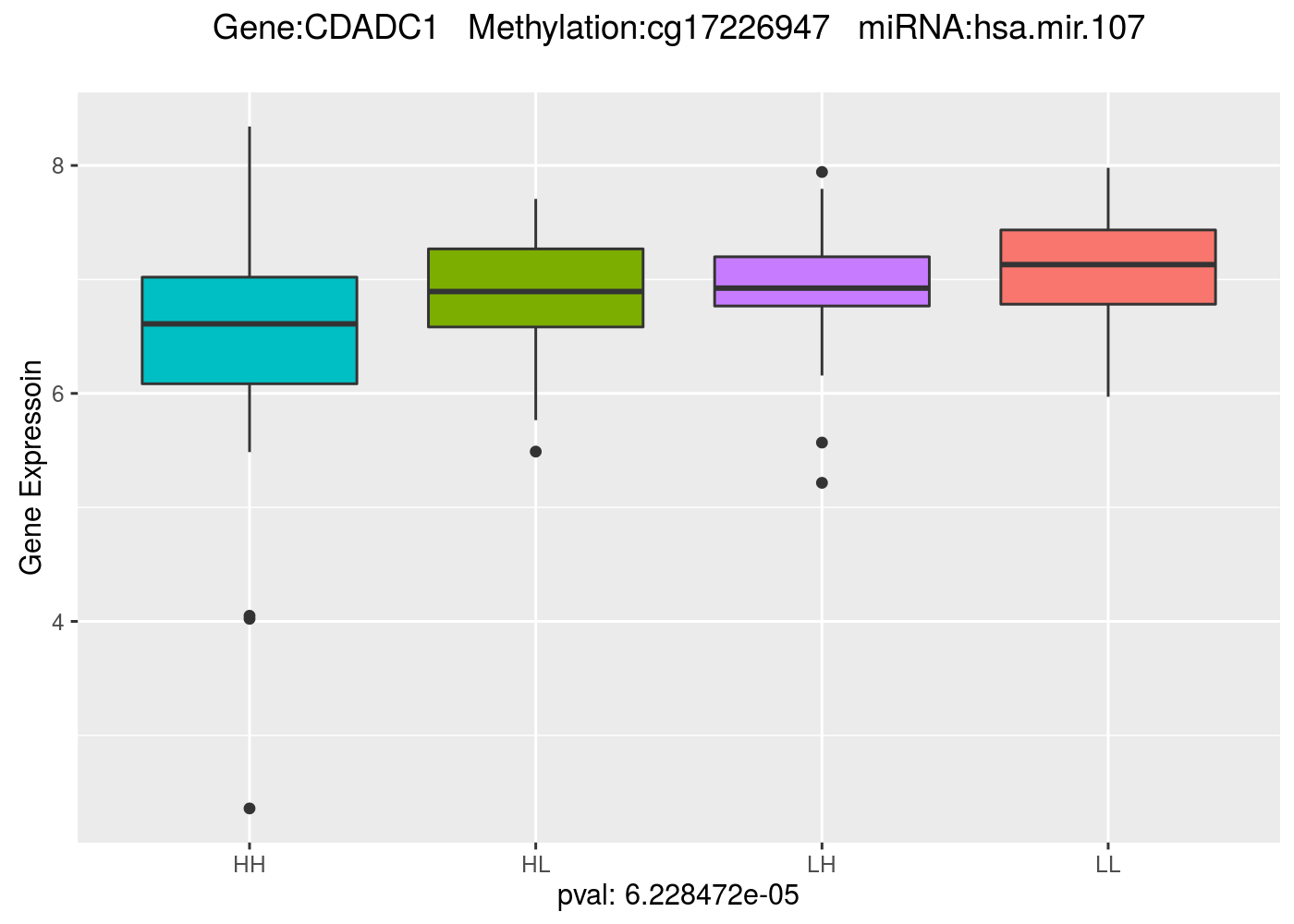

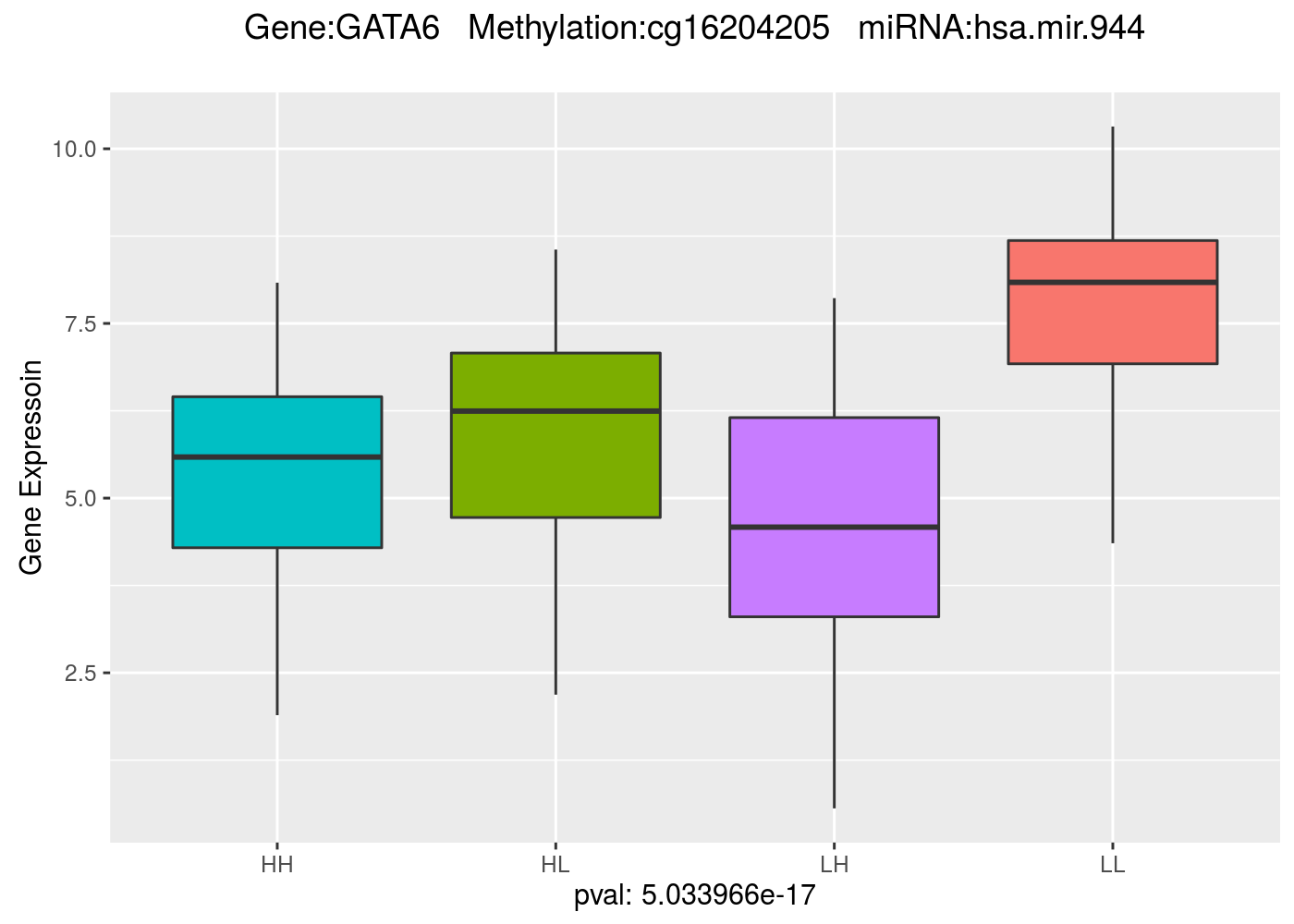

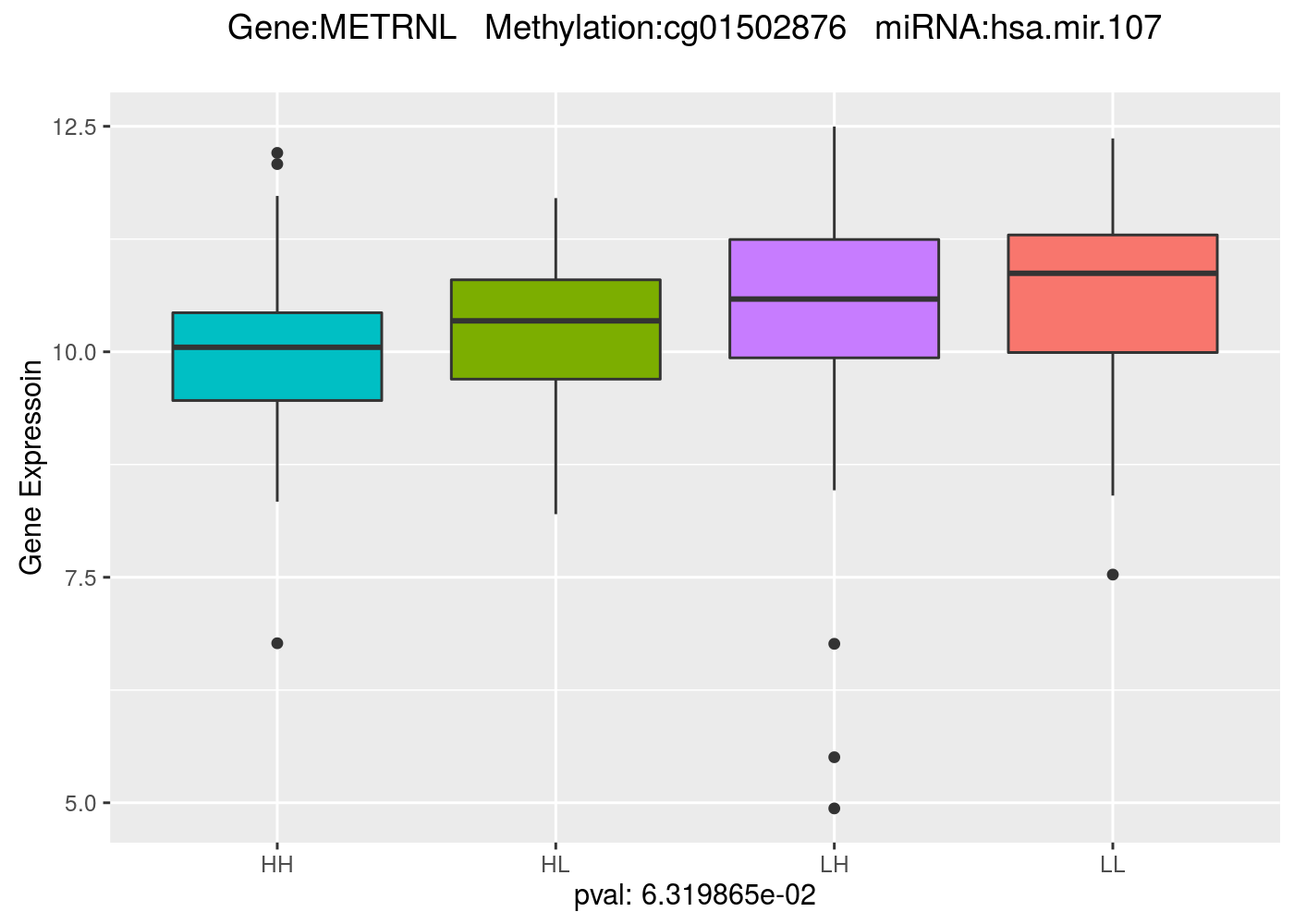

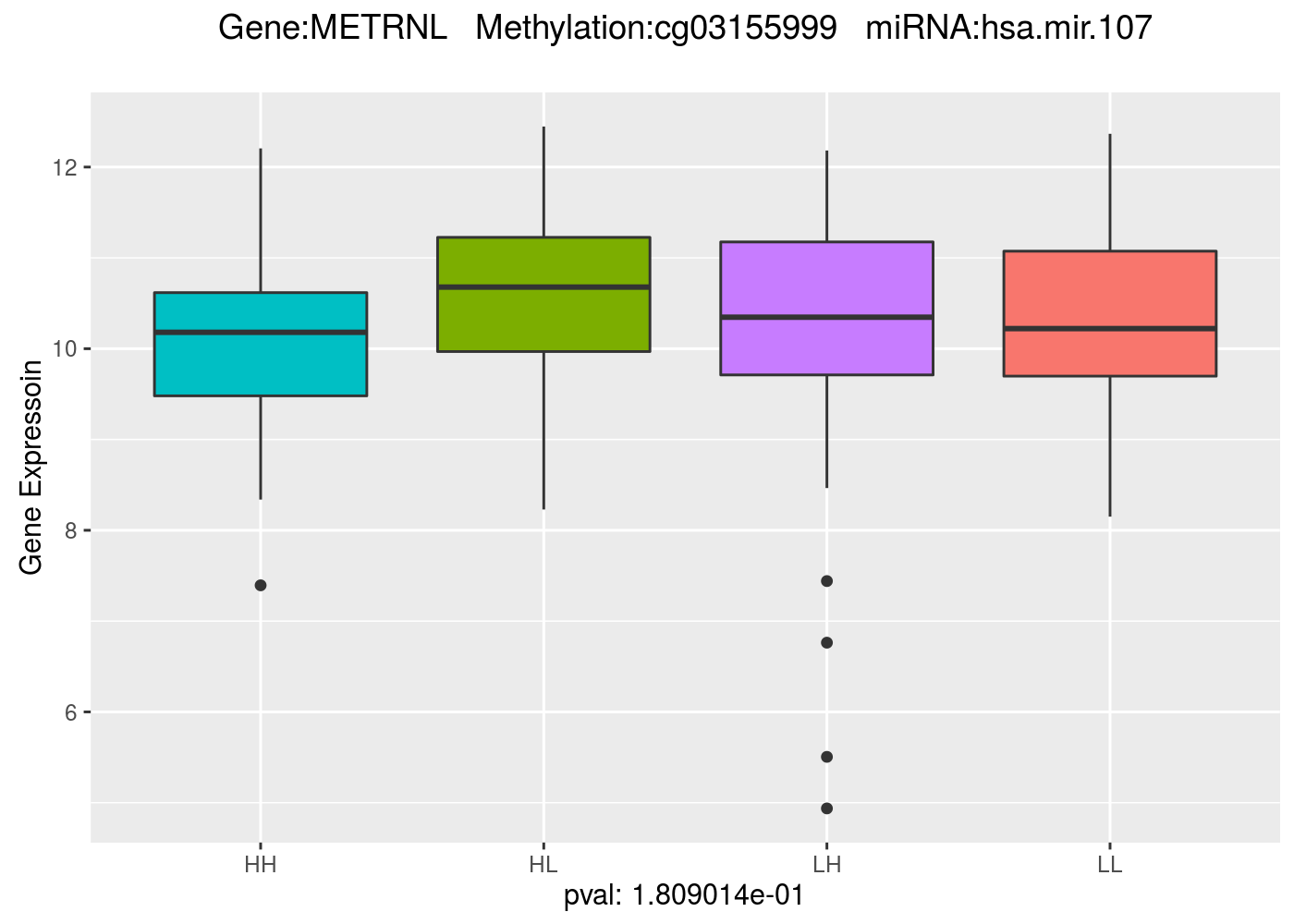

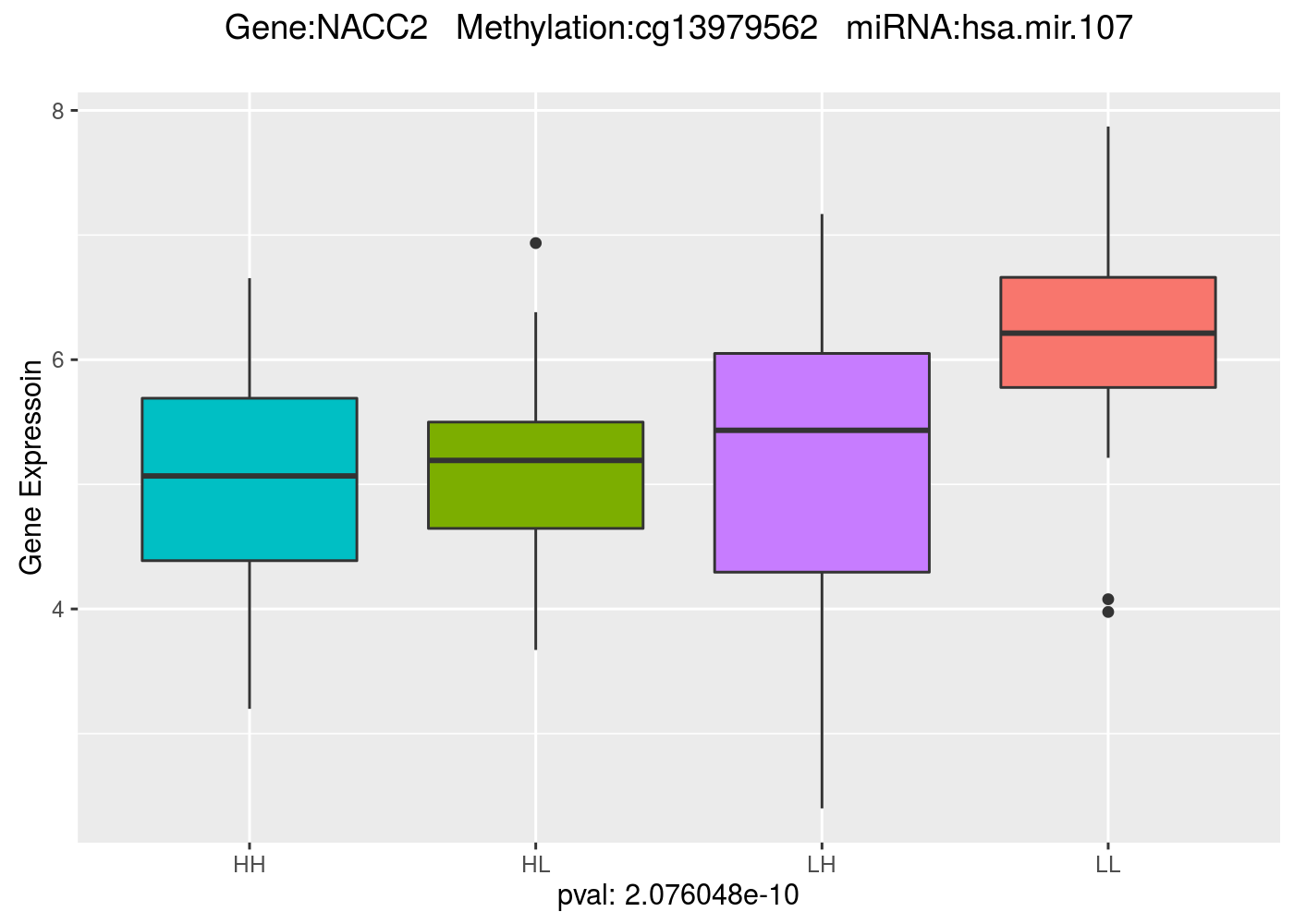

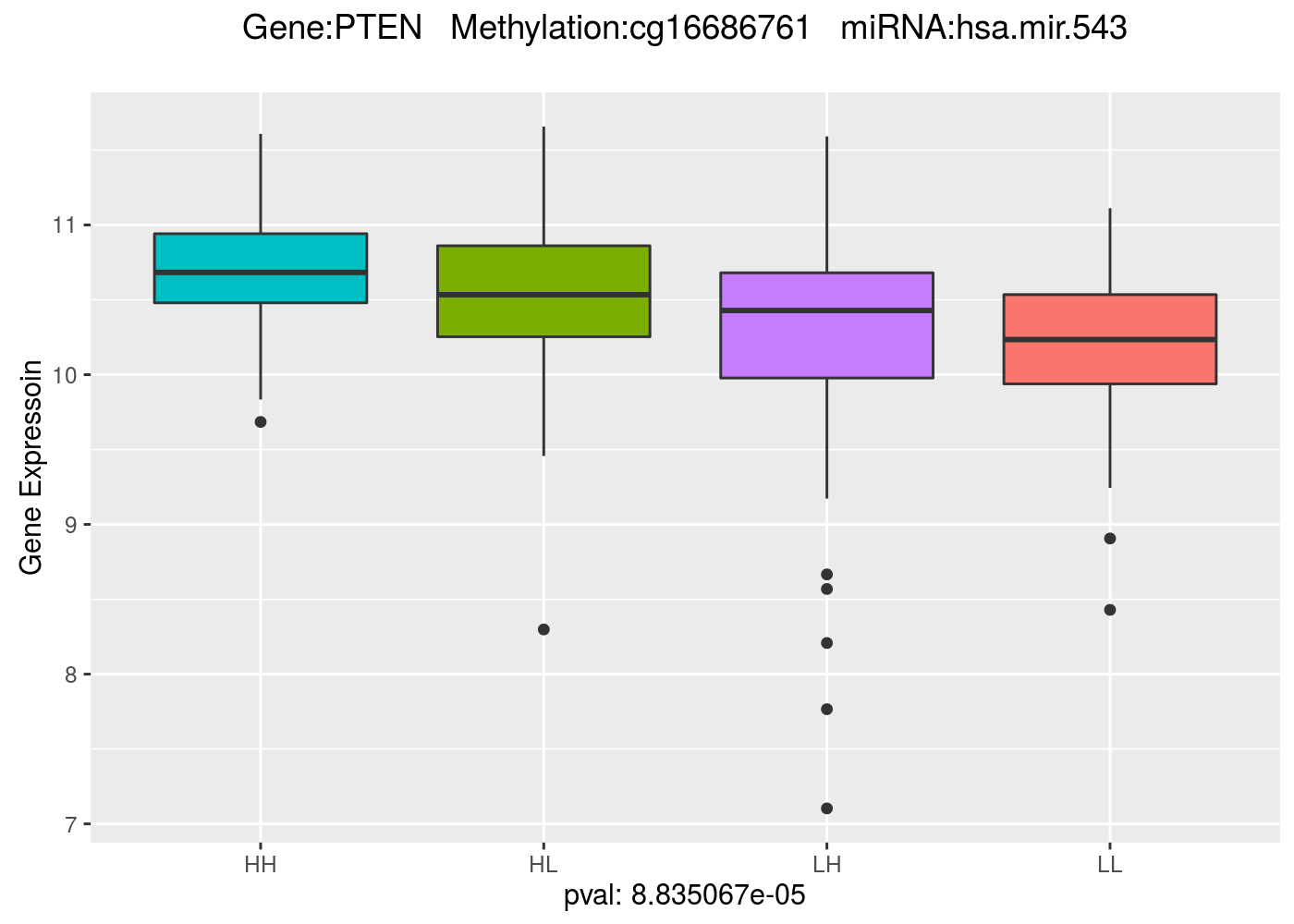

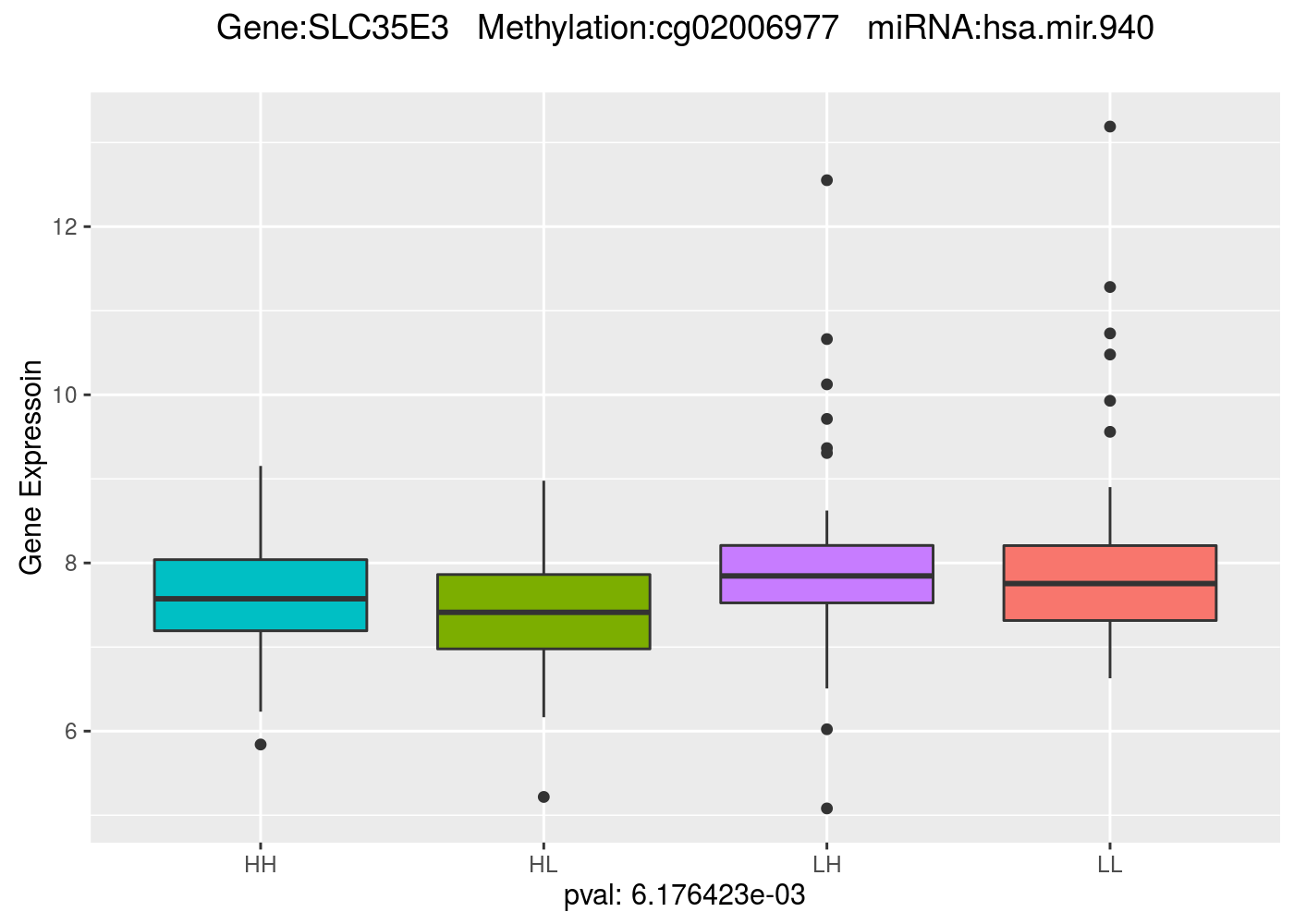
**

**
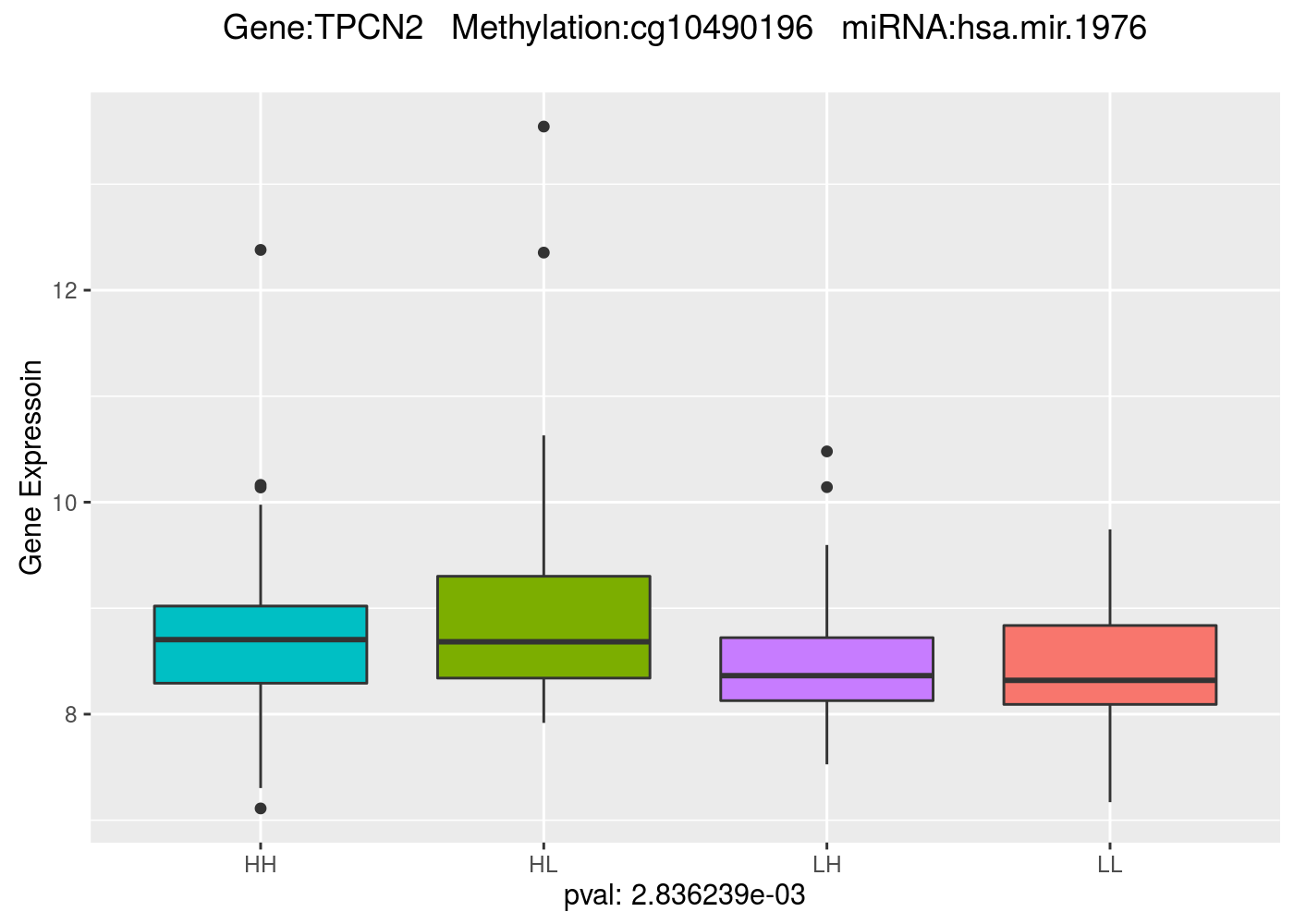
**

**
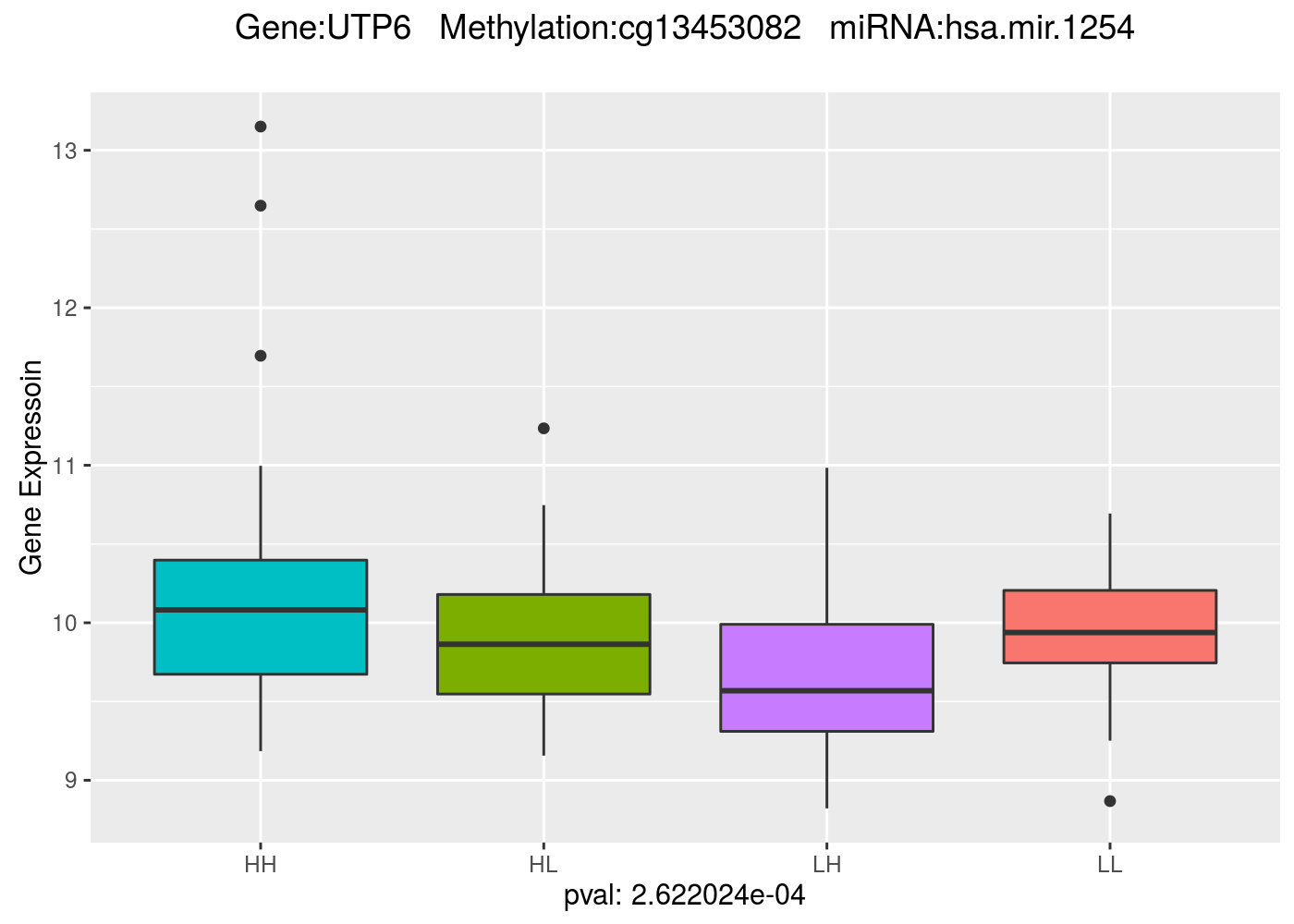
**

**
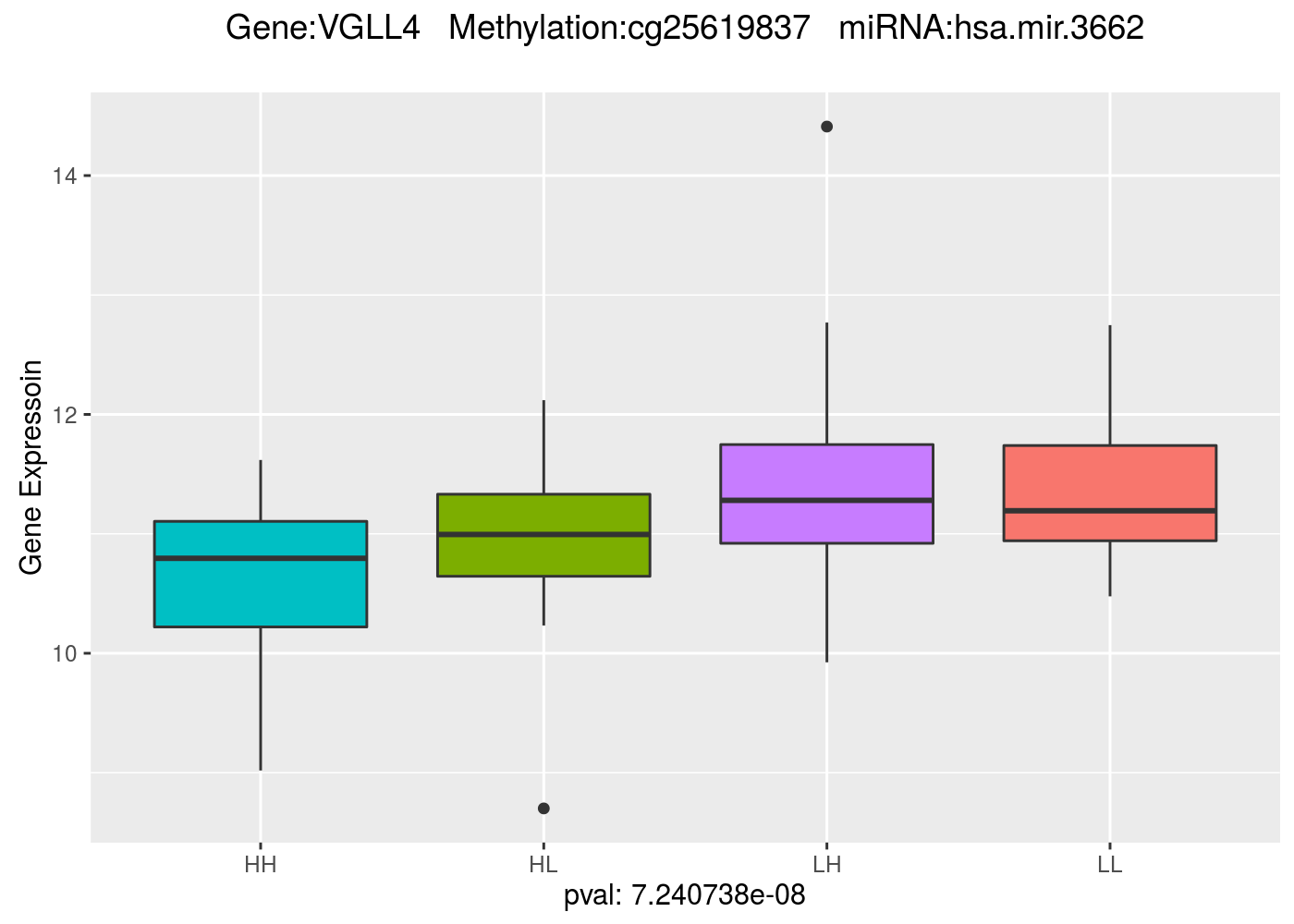
**

**Figure S5.** Survival analysis across four subgroups (LL, LH, HL, and HH)

**
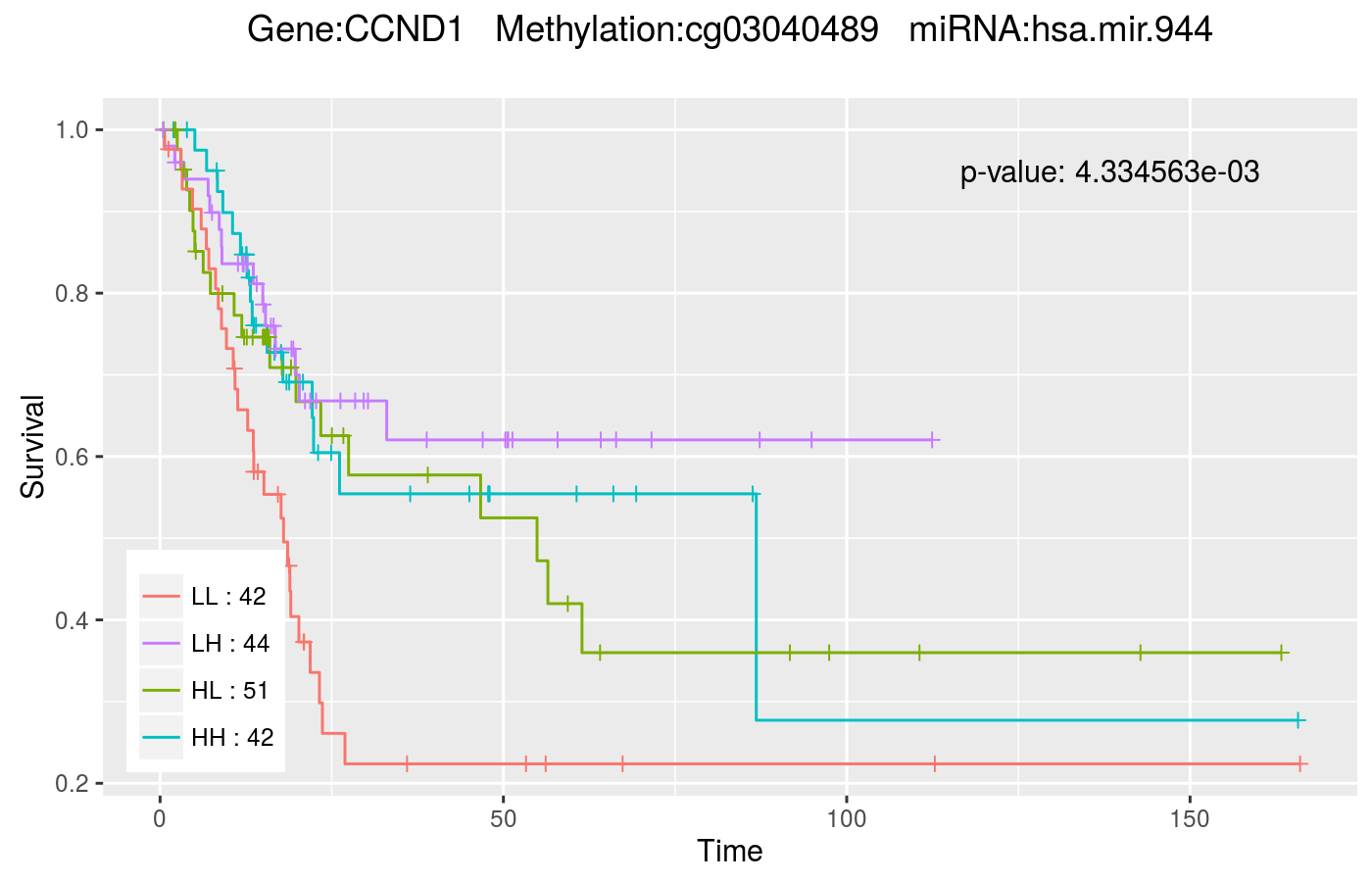

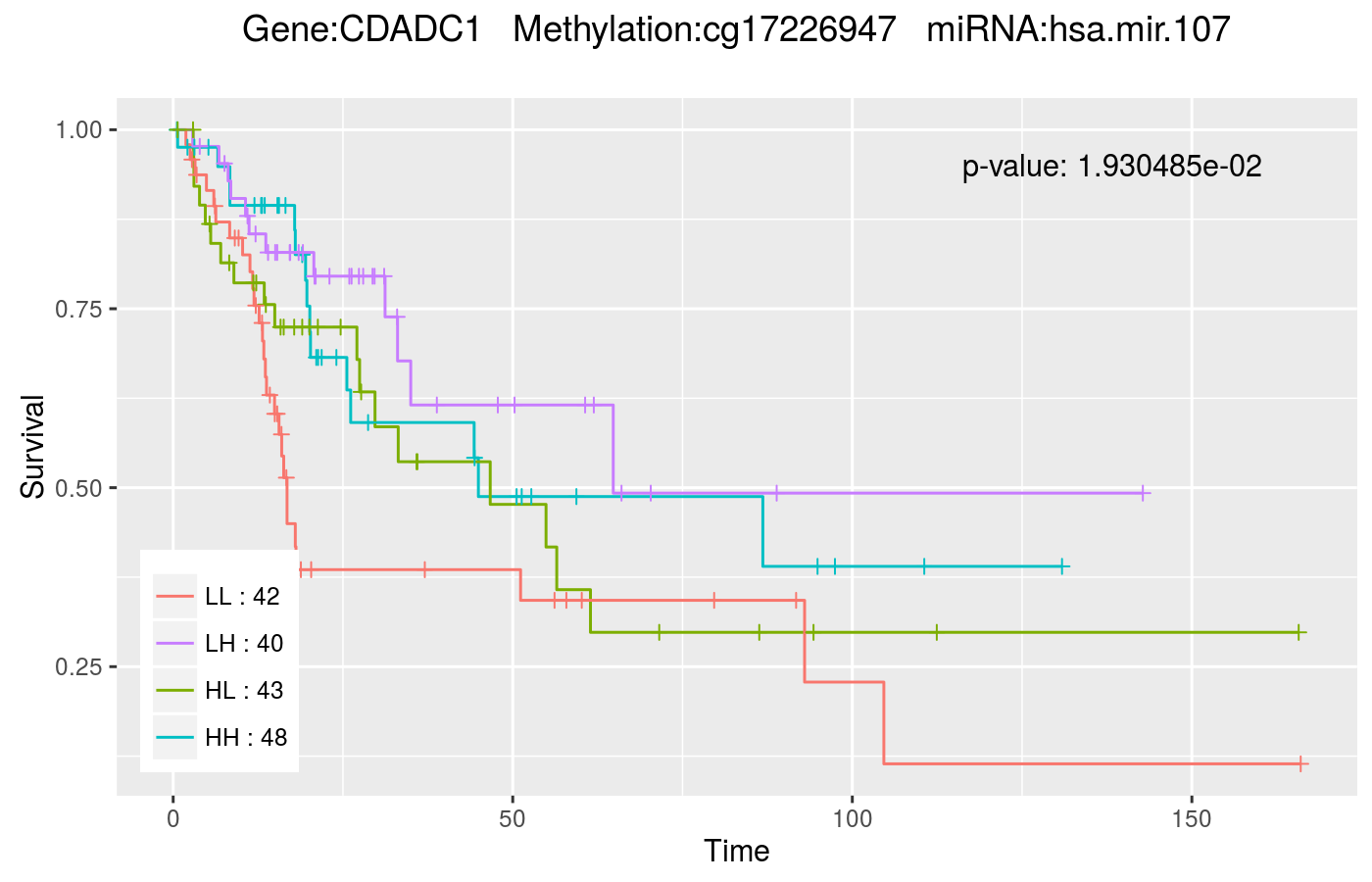

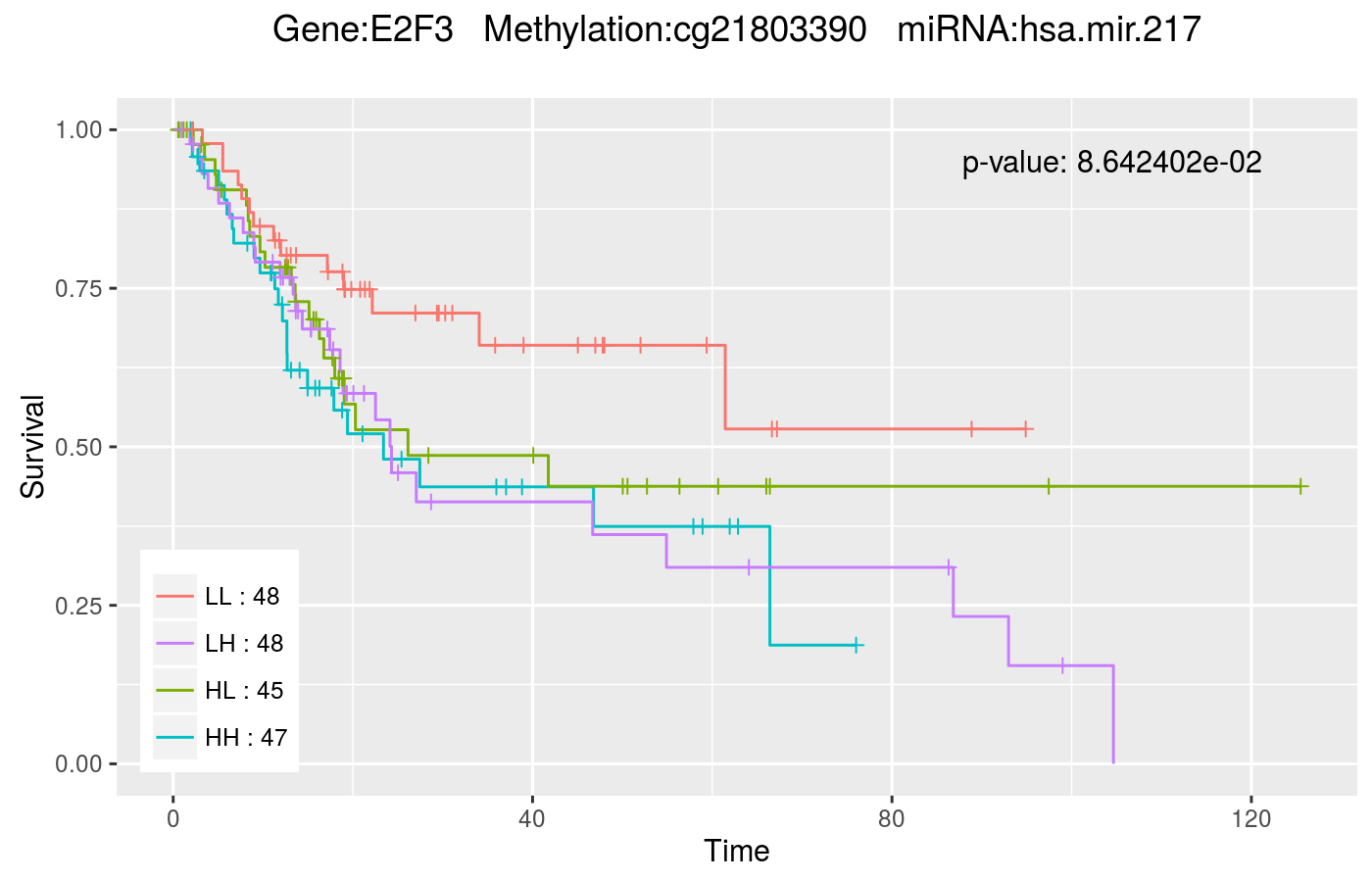

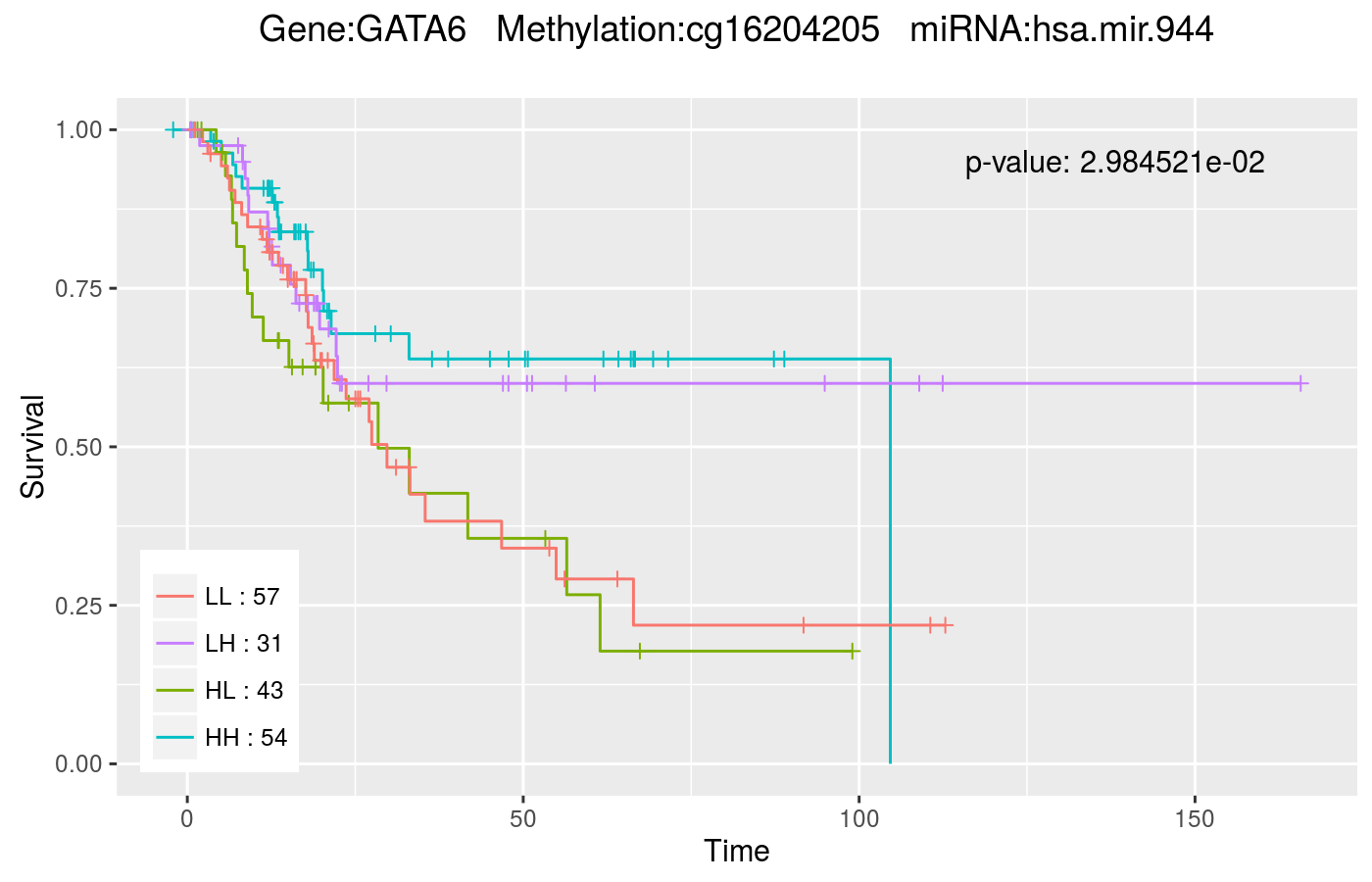

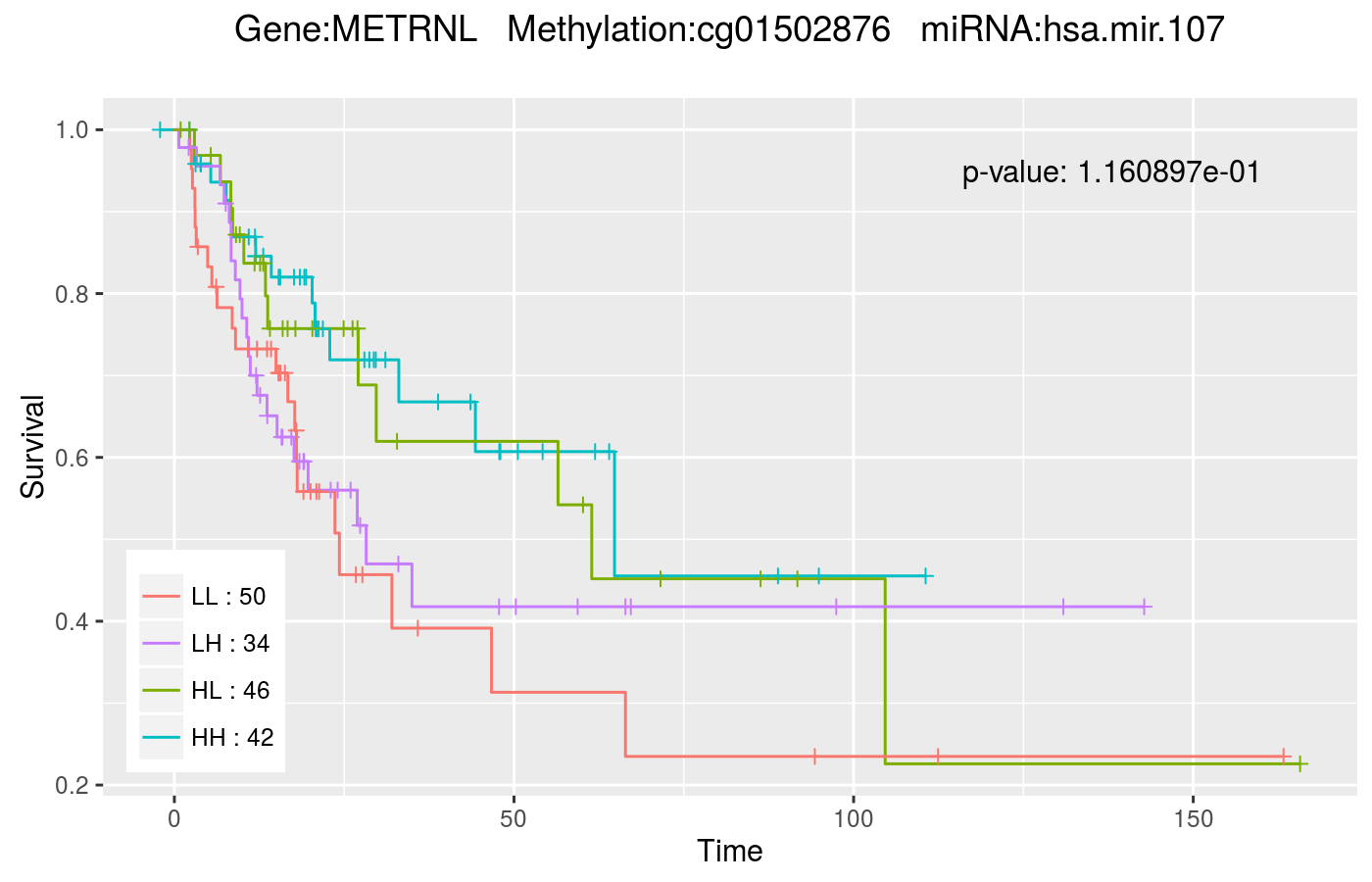

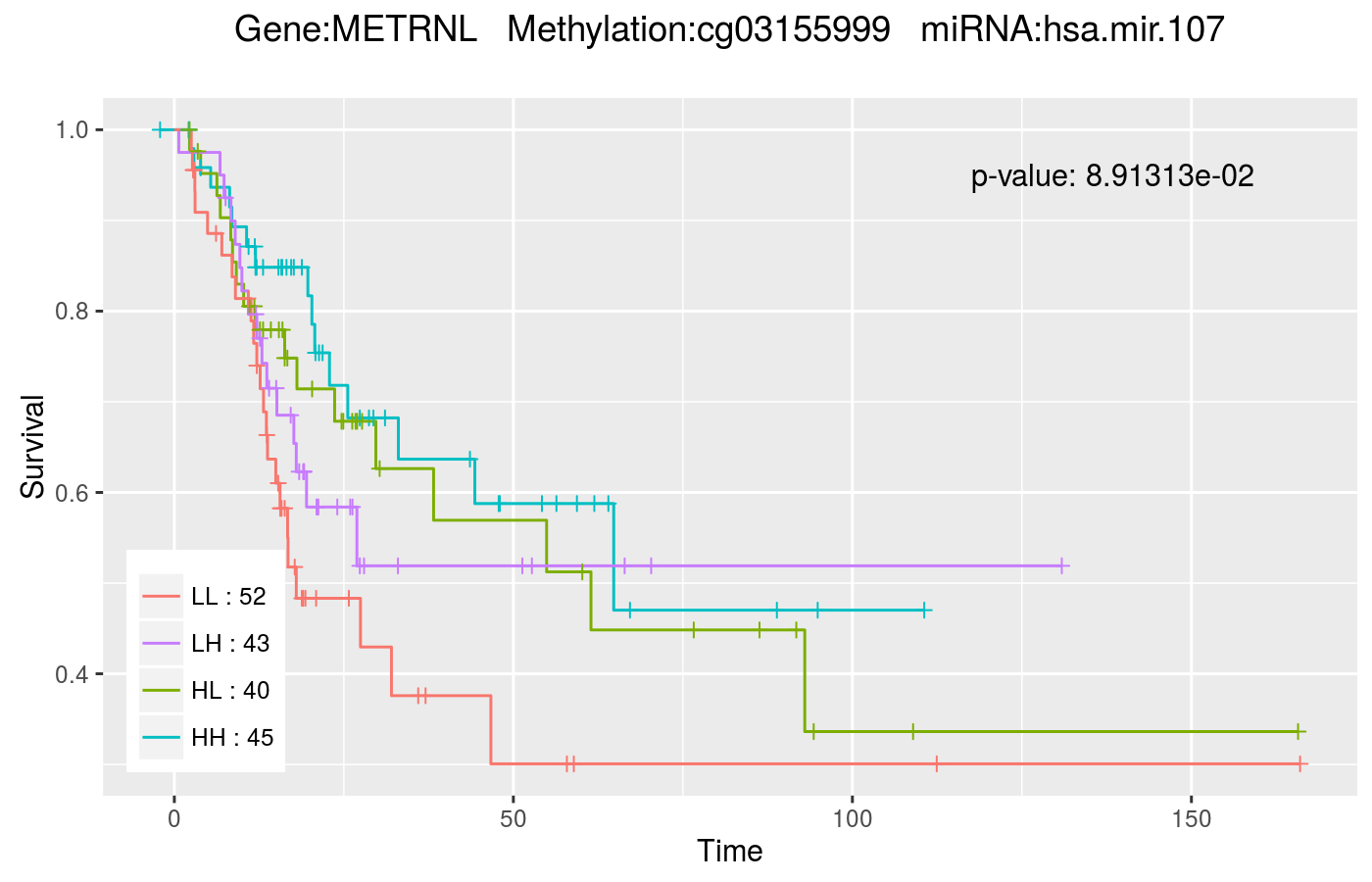

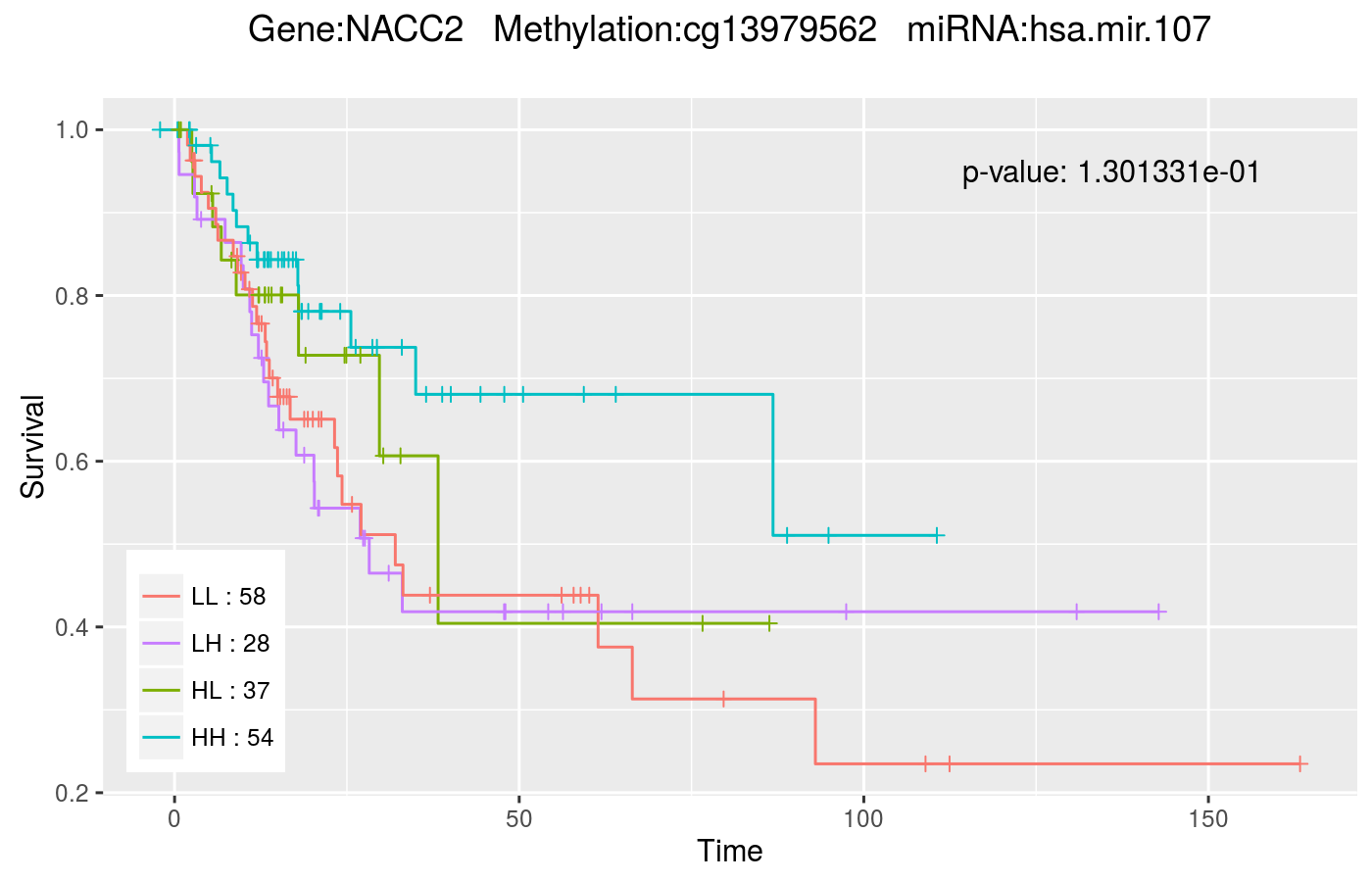

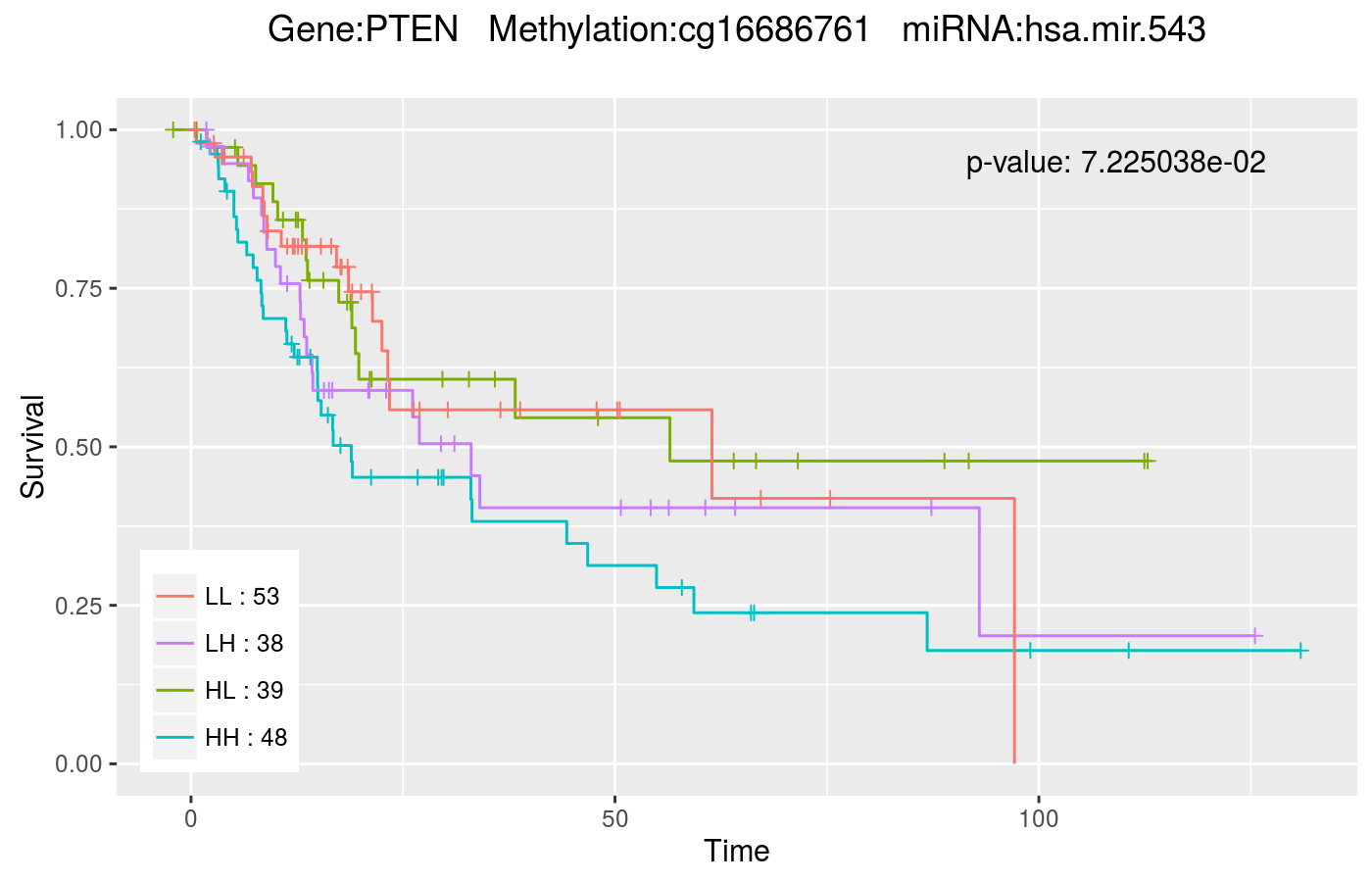

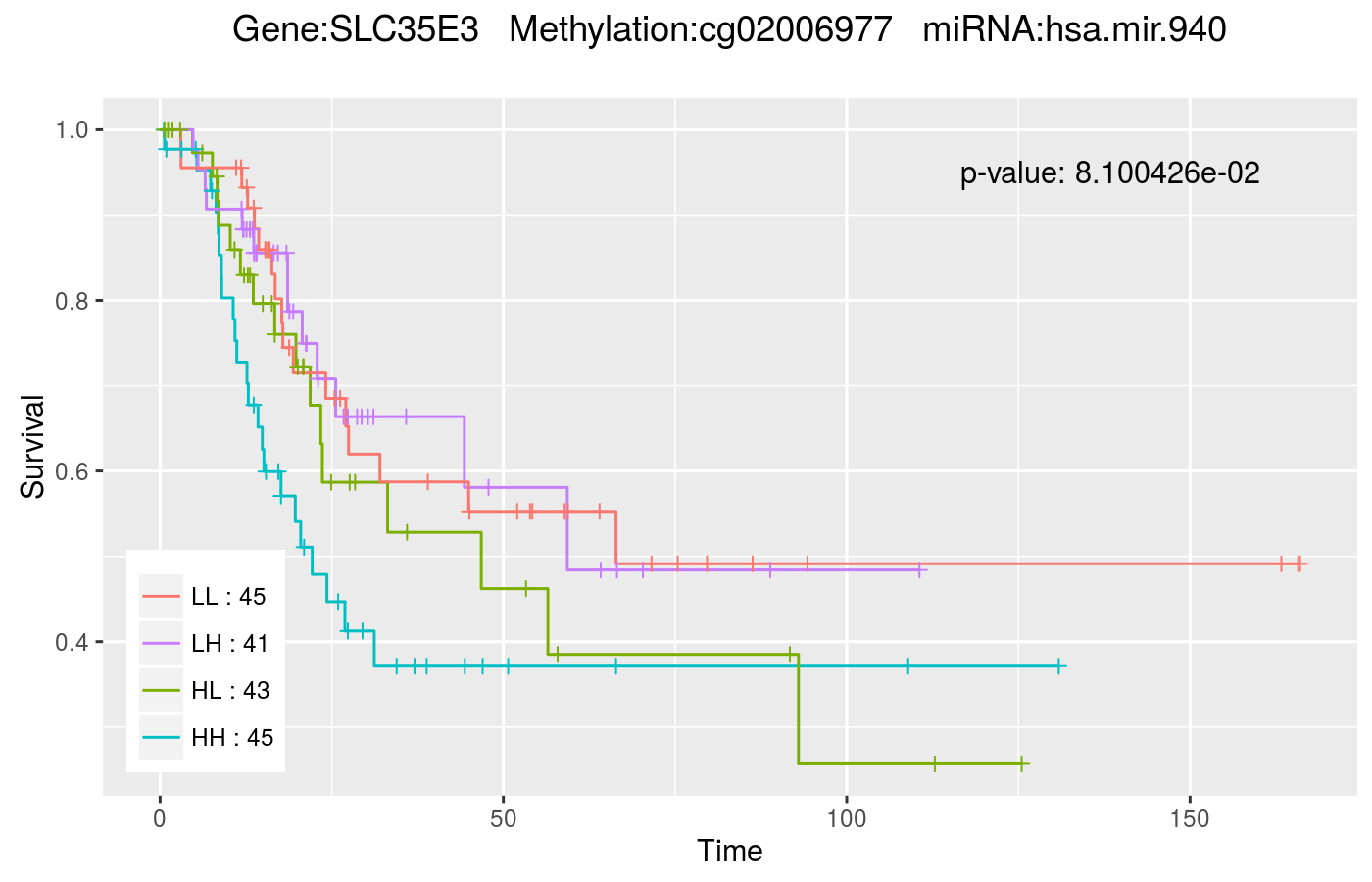

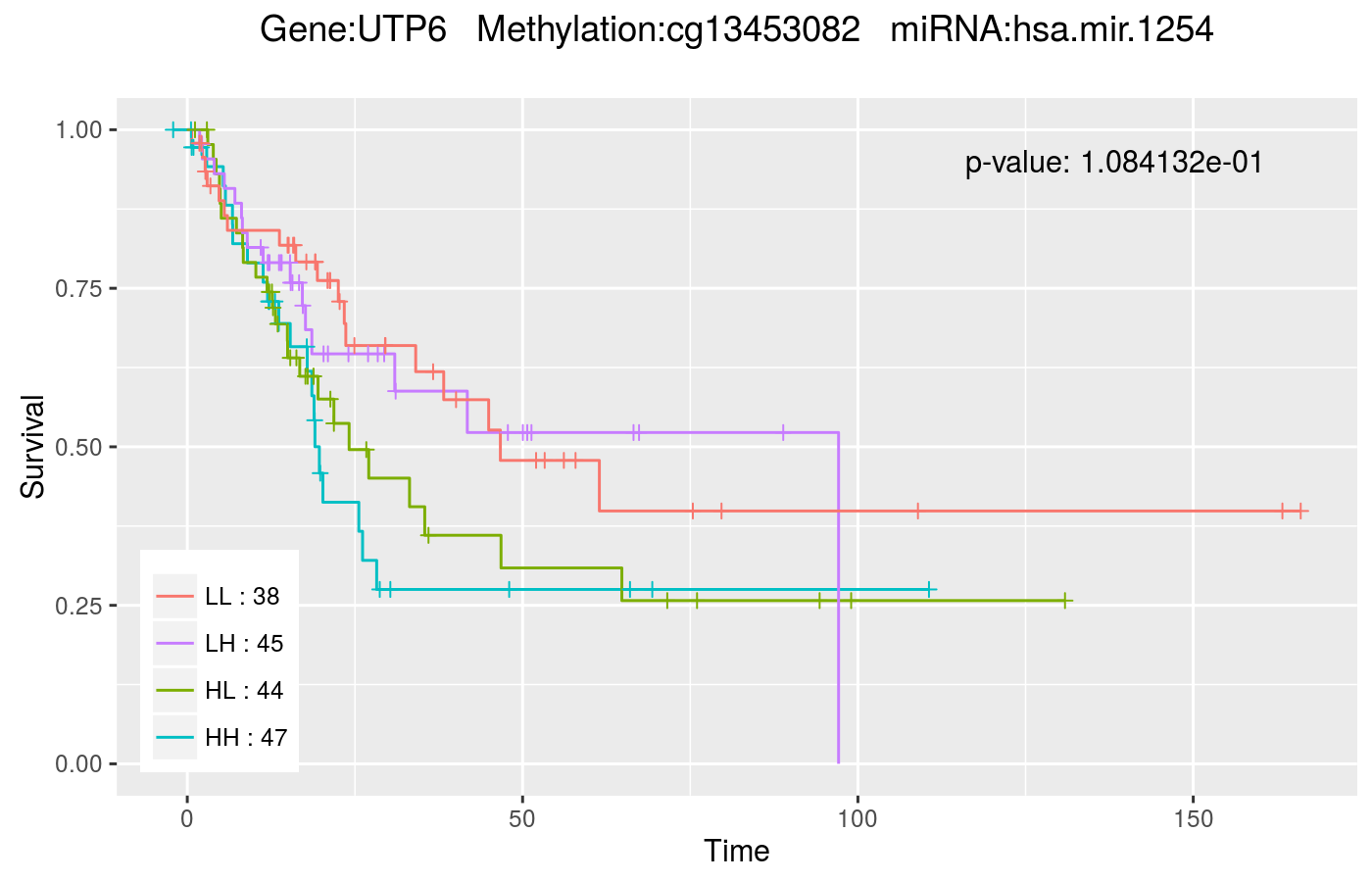

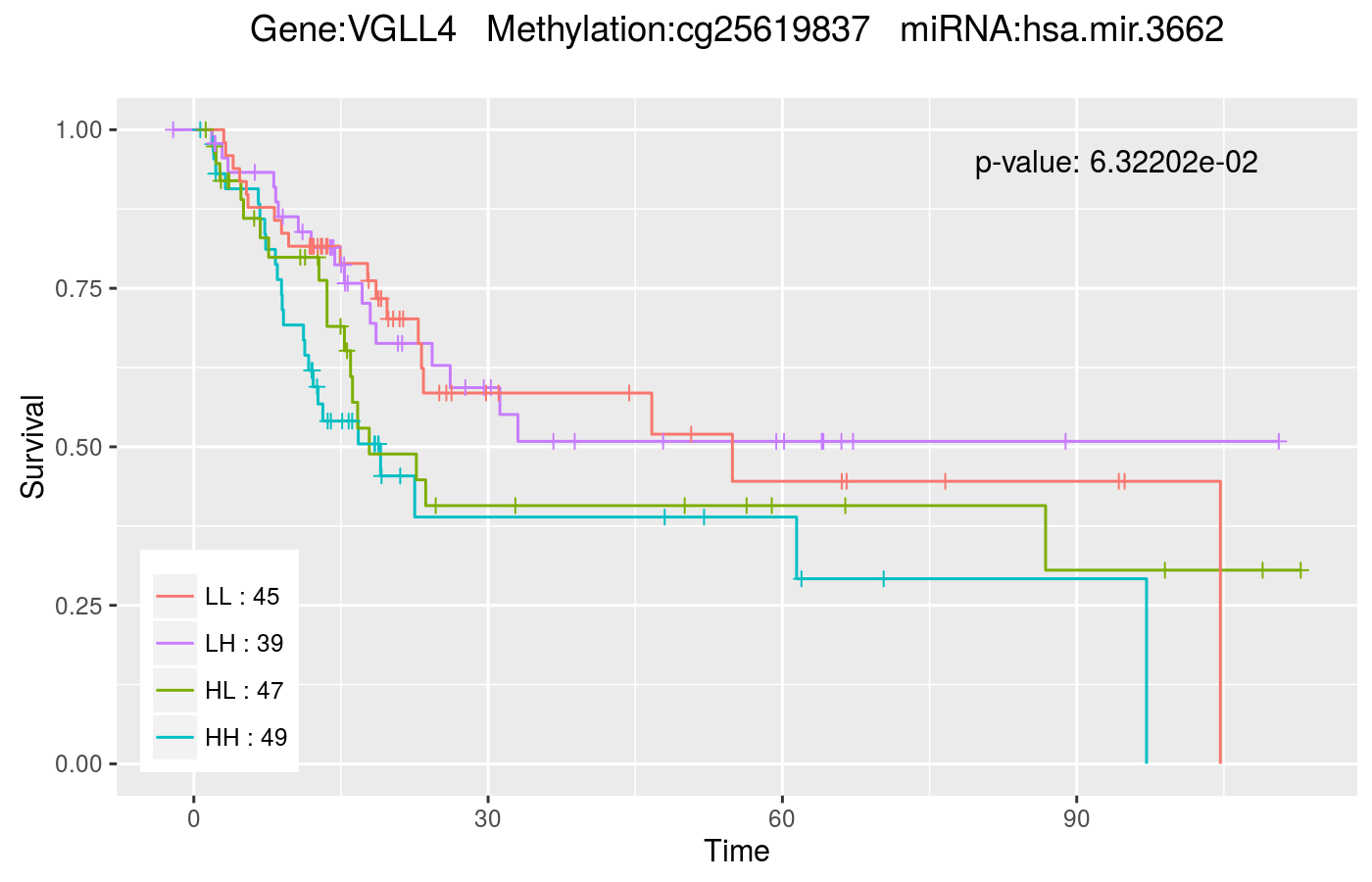
**
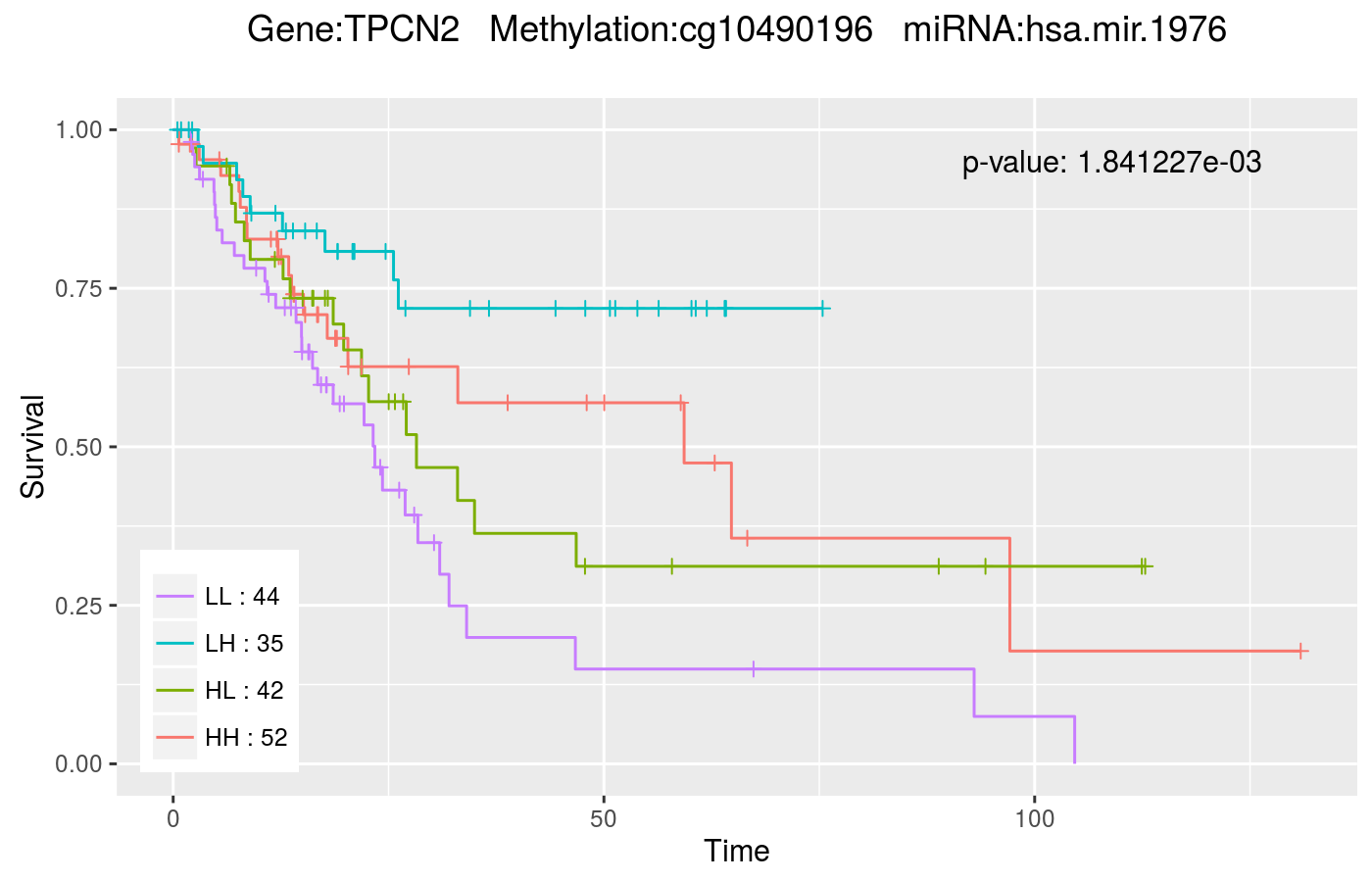

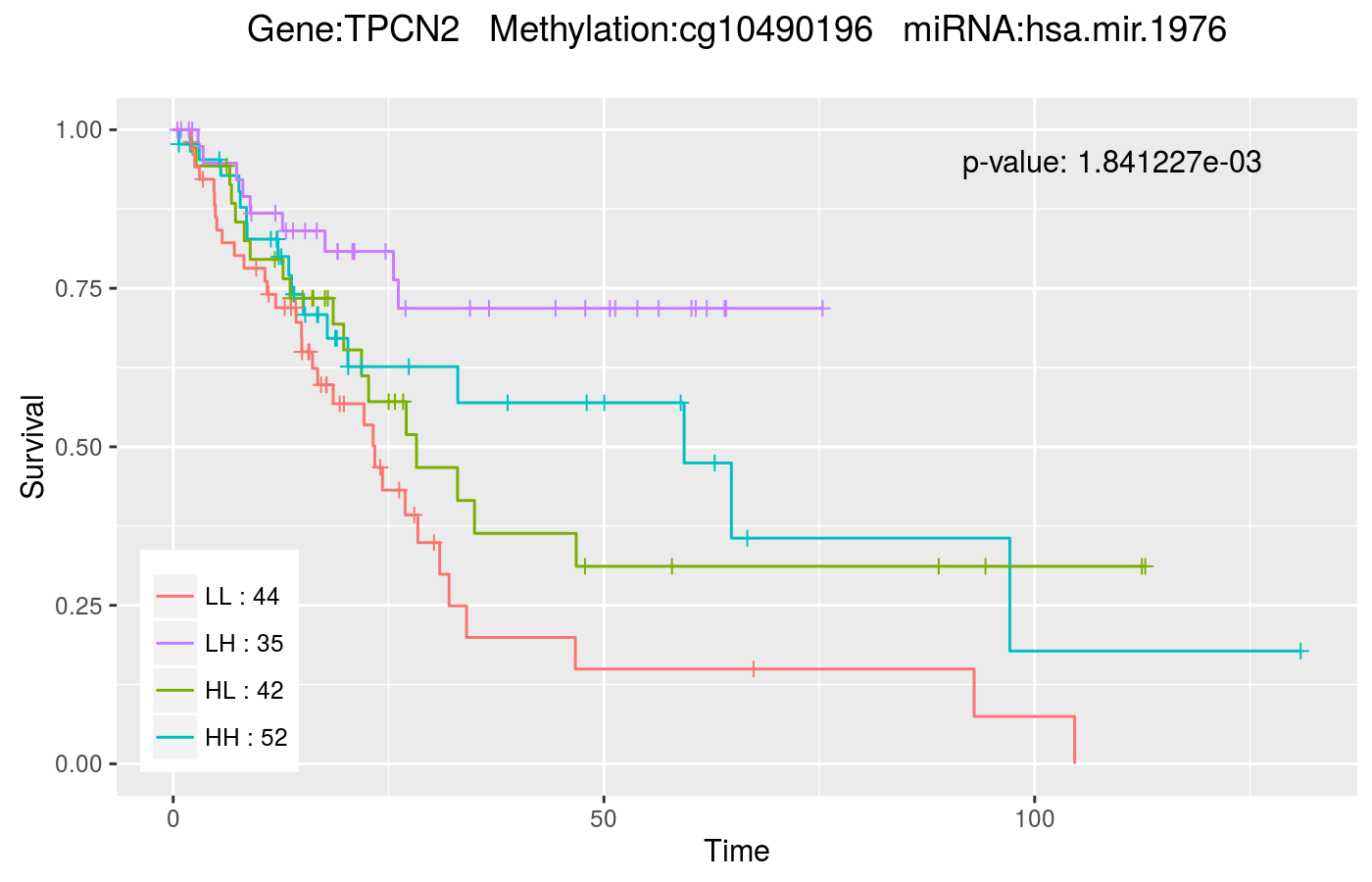

Supplement: Additional file 1: — Supplementary information. Table S1. Significant epigenetic interactions between miRNA and methylation associated with target genes. Table S2. Significant epigenetic interactions between miRNA and methylation associated with target genes for papillary subtype. Table S3. Significant epigenetic interactions between miRNA and methylation associated with target genes for non-papillary subtype. Table S4. Summary of overall survival analysis results. Figure S1. Venn Diagram of Significant target genes for papillary, non-papillary subtypes. Figure S2. Survival analysis between two subgroups (LL and HH). Figure S3. Gene expression boxplot for two subgroups (LL and HH). Figure S4. Gene expression boxplot for four subgroups (LL, LH, HL, and HH). Figure S5. Survival analysis across four subgroups (LL, LH, HL, and HH). (DOCX 2368 kb) [file 12920_2017_269_MOESM1_ESM.docx]
